# Robust optimization of SVM hyperparameters in the classification of bioactive compounds

**DOI:** 10.1186/s13321-015-0088-0

**Published:** 2015-08-14

**Authors:** Wojciech M Czarnecki, Sabina Podlewska, Andrzej J Bojarski

**Affiliations:** Faculty of Mathematics and Computer Science, Jagiellonian University, 6 S. Lojasiewicza Street, 30-348 Krakow, Poland; Department of Medicinal Chemistry, Institute of Pharmacology, Polish Academy of Sciences, 12 Smetna Street, 31-343 Krakow, Poland; Faculty of Chemistry, Jagiellonian University, 3 Ingardena Street, 30-060 Krakow, Poland

**Keywords:** Compounds classification, Virtual screening, Support Vector Machine, Parameters optimization, Bayesian optimization

## Abstract

**Background:**

Support Vector Machine has become one of the most popular machine learning tools used in virtual screening campaigns aimed at finding new drug candidates. Although it can be extremely effective in finding new potentially active compounds, its application requires the optimization of the hyperparameters with which the assessment is being run, particularly the *C* and $\gamma$ values. The optimization requirement in turn, establishes the need to develop fast and effective approaches to the optimization procedure, providing the best predictive power of the constructed model.

**Results:**

In this study, we investigated the Bayesian and random search optimization of Support Vector Machine hyperparameters for classifying bioactive compounds. The effectiveness of these strategies was compared with the most popular optimization procedures—grid search and heuristic choice. We demonstrated that Bayesian optimization not only provides better, more efficient classification but is also much faster—the number of iterations it required for reaching optimal predictive performance was the lowest out of the all tested optimization methods. Moreover, for the Bayesian approach, the choice of parameters in subsequent iterations is directed and justified; therefore, the results obtained by using it are constantly improved and the range of hyperparameters tested provides the best overall performance of Support Vector Machine. Additionally, we showed that a random search optimization of hyperparameters leads to significantly better performance than grid search and heuristic-based approaches.

**Conclusions:**

The Bayesian approach to the optimization of Support Vector Machine parameters was demonstrated to outperform other optimization methods for tasks concerned with the bioactivity assessment of chemical compounds. This strategy not only provides a higher accuracy of classification, but is also much faster and more directed than other approaches for optimization. It appears that, despite its simplicity, random search optimization strategy should be used as a second choice if Bayesian approach application is not feasible.

**Graphical abstract:**
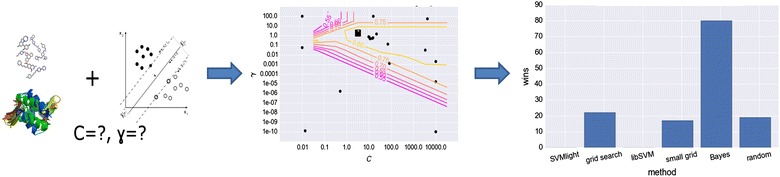
The improvement of classification accuracy obtained after the application of Bayesian approach to the optimization of Support Vector Machines parameters.

**Electronic supplementary material:**

The online version of this article (doi:10.1186/s13321-015-0088-0) contains supplementary material, which is available to authorized users.

## Background

The application of computational methods at various stages of drug design and development has become a vital part of the process. As the methods developed become constantly more effective, despite the aims at optimizing their performance, the focus of the attention shifts away from performance optimization to the minimization of requirements for computational resources. The attainment of both effectiveness and the desired speed has been responsible for the recent extreme popularity of machine learning (ML) methods in computer-aided drug design (CADD) approaches. Machine learning methods are mostly used for virtual screening (VS) tasks, in which they are supposed to identify potentially active compounds in large databases of chemical structures. One of the most widely used ML methods in CADD is the Support Vector Machine (SVM). Although it has a potential of providing very high VS performance, its application requires the optimization of the parameters used during the training process, which was proved to be crucial for obtaining accurate predictions. To date, various approaches have been developed to make SVM faster and more effective. In cheminformatics applications, the most popular optimization strategies are grid search [1, 2] and heuristic choice [3, 4]. Depending on the problem, they are able to provide high classification accuracy—for example Wang et al. obtained 86% of accuracy in the classification of hERG potassium channel inhibitors for the heuristic choice of the SVM parameters  [4]. On the other hand, Hamman et al. [1] were able to evaluate the cytochrome P450 activities with 66–83% of accuracy using grid search method of SVM parameters optimization. The need for optimizing SVM parameters is undeniable, as classification efficiency can change dramatically for various parameters values. A high computational cost of a systematic search over a predefined set of parameters’ values is a trigger for development of new optimization algorithms. In recent years, Bayesian optimization [5, 6] (including gaussian processes [7]) and random search-based selection [8] have become more popular [9, 10]. As those approaches were not explored so far in the field of cheminformatics, we analyze their impact on classification accuracy and, more importantly, the speed and ease of use, that these approaches have lent to the optimization of SVM hyperparameters in the search for bioactive compounds.

### Hyperparameters optimization

In the classical ML approach to a classification problem, we are given a training set $\left\{ (x_i,y_i) \right\} _{i=1}^N$ (with $x_i$ representing samples’ features, in our case—fingerprint, and $y_i$ being the class assignment) and we try to build a predictive model based on these data using a training algorithm that sets the parameters *w* (for example the weight of each fingerprint element) for fixed hyperparameters $\varvec{\lambda }$ (for example a type of SVM kernel, the regularization strength *C* or the width of the RBF kernel $\gamma$). In other words, given an objective $\mathcal {S}$ that must be maximized, we are supposed to solve the following problem:

$$\begin{aligned} \underset{w}{\text {maximize }} \mathcal {S}\left( w | \left\{ (x_i,y_i) \right\} _{i=1}^N, \varvec{\lambda }\right) . \end{aligned}$$

While this problem is often easily solvable (for example, in SVM, $\mathcal {S}$ is a concave function, and thus, we can find the maximum by using a simple steepest ascent algorithm), in general, it is very hard to find an optimal $\varvec{\lambda }$. This difficulty stems from the very complex shape of the $\mathcal {S}$ function once we treat $\varvec{\lambda }$ as its arguments, which results in the joint optimization of the model parameters (*w*) and the set of hyperparameters ($\varvec{\lambda }$):

$$\begin{aligned} \underset{w, \varvec{\lambda }}{\text {maximize }} \mathcal {S}\left( w, \varvec{\lambda }| \left\{ (x_i,y_i) \right\} _{i=1}^N\right) . \end{aligned}$$

A basic method for solving this problem is a grid search-based approach, which simply samples the set of possible $\varvec{\lambda }$ values in a regular manner. For example, we choose the parameter *C* for a SVM in a geometrical progression, obtaining the values $\varvec{\lambda }_1, \ldots, \varvec{\lambda }_k$ and returning the best solution among each of the subproblems:

$$\begin{aligned} w_i = \arg \max _w \mathcal {S}\left( w| \left\{ (x_i,y_i) \right\} _{i=1}^N, \varvec{\lambda }_i\right) . \end{aligned}$$

While such an approach guarantees finding the global optimum for $k \rightarrow \infty$, it might be extremely computationally expensive, as we need to train *k* classifiers, each of which can take hours. Instead, we can actually try to solve the optimization problem directly by performing an adaptive process that on one hand tries to maximize the objective function and on the other hand samples the possible $\varvec{\lambda }$ space intelligently in order to minimize the number of classifier trainings. The main idea behind Bayesian optimization for such a problem is to use *all of the information* gathered in previous iterations for performing the next step. It is apparent that grid search-based methods violate this assumption as we do not use any knowledge coming out from the results of models trained with other $\varvec{\lambda }$ values.

We can consider this problem as the process of finding the maximum for $f(\varvec{\lambda })$, defined as

$$\begin{aligned} f(\varvec{\lambda }) = \max _w \mathcal {S}\left( w| \left\{ (x_i,y_i) \right\} _{i=1}^N, \varvec{\lambda }\right) . \end{aligned}$$

Unfortunately, *f* is an unknown function and we cannot compute its gradient, Hessian, or any other characteristics that could guide the optimization process. The only action we can perform is to obtain a value for *f* at a given point. However, doing so is very expensive (because it requires training a classifier); thus, we need a fast (with respect to evaluating the function), derivative-free optimization technique to solve this problem.

For the task under consideration, $\mathcal {S}$ is the accuracy of the resulting SVM model with the RBF kernel, and $\varvec{\lambda }=\{ C, \gamma \}$ is the set of two hyperparameters that we must fit to optimize the SVM performance to predict the bioactivity of compounds, which (loosely speaking) is measured by *f*.

**Table 1:** Details of the classification experiments performed

Targets	Fingerprints	Optimization method	${C \mathrm\;{\text{and}}\; \gamma \mathrm\;{\text{range}}}$
			No of iterations
5-HT$_\text {2A}$	EstateFP	Bayes	$\log _{10}(C) \in [-2, 5]$
5-HT$_\text {2C}$	ExtFP	Random	${\log _{10}(\gamma ) \in [-10, 3]}$
5-HT$_\text {6}$	KlekFP	Grid search	20, 30, 50, 75, 100, 150
5-HT$_\text {7}$	MACCSFP	Small grid	
CDK2	PubchemFP	SVMlight	
M$_\text {1}$	SubFP	libSVM	
ERK2			
AChE			
A$_\text {1}$			
alpha2AR			
beta1AR			
beta3AR			
CB1			
DOR			
D$_\text {4}$			
H$_\text {1}$			
H$_\text {3}$			
HIVi			
IR			
ABL			
HLE			

## Results and discussion

Six SVM optimization approaches were evaluated in the classification experiments of compounds possessing activity towards 21 protein targets, represented by six different fingerprints (Table 1).

**Fig. 1:**
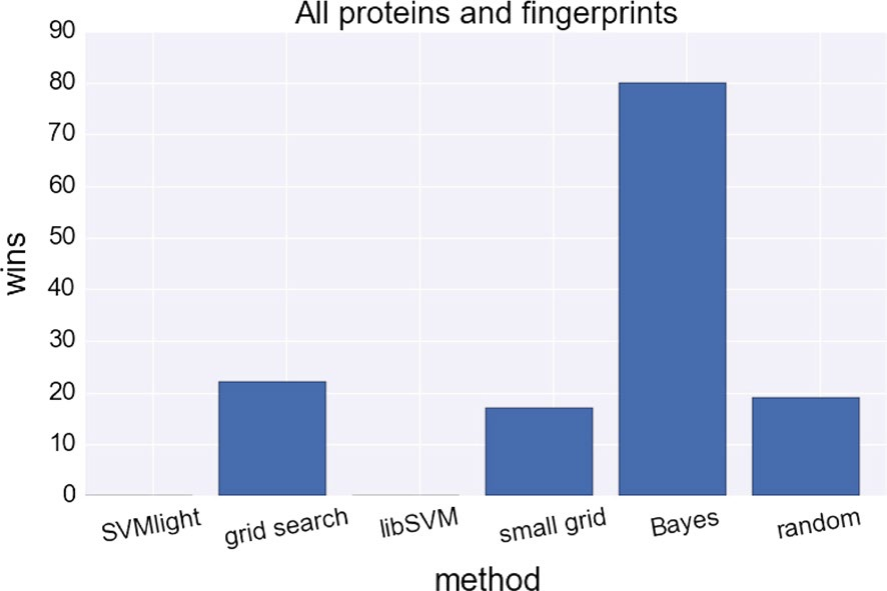
Global analysis of classification accuracy obtained for different methods for SVM parameters optimization expressed as the number of experiments in which a particular strategy provided the highest accuracy values.

### Classification effectiveness analysis

A global analysis of the classification efficiency revealed that Bayesian optimization definitely outperformed the other methods of SVM parameters’ optimization (Fig. 1). For a particular target and fingerprint, Bayesian approach provided a higher classification accuracy in 80 experiments, a significantly greater number than the other strategies (22 for the runner-up: grid search). On the other hand, the SVMlight and libSVM were definitely the least effective methods of SVM usage; they did not provide the highest accuracy values for any of the target/fingerprint combinations. This result is an obvious consequence of the fact that SVMlight and libSVM are just basic heuristics and their results cannot be comparable with any hyperparameters optimization technique. Interestingly, libSVM achieved much better results than SVMlight even though its heuristic is much simpler.

**Fig. 2:**
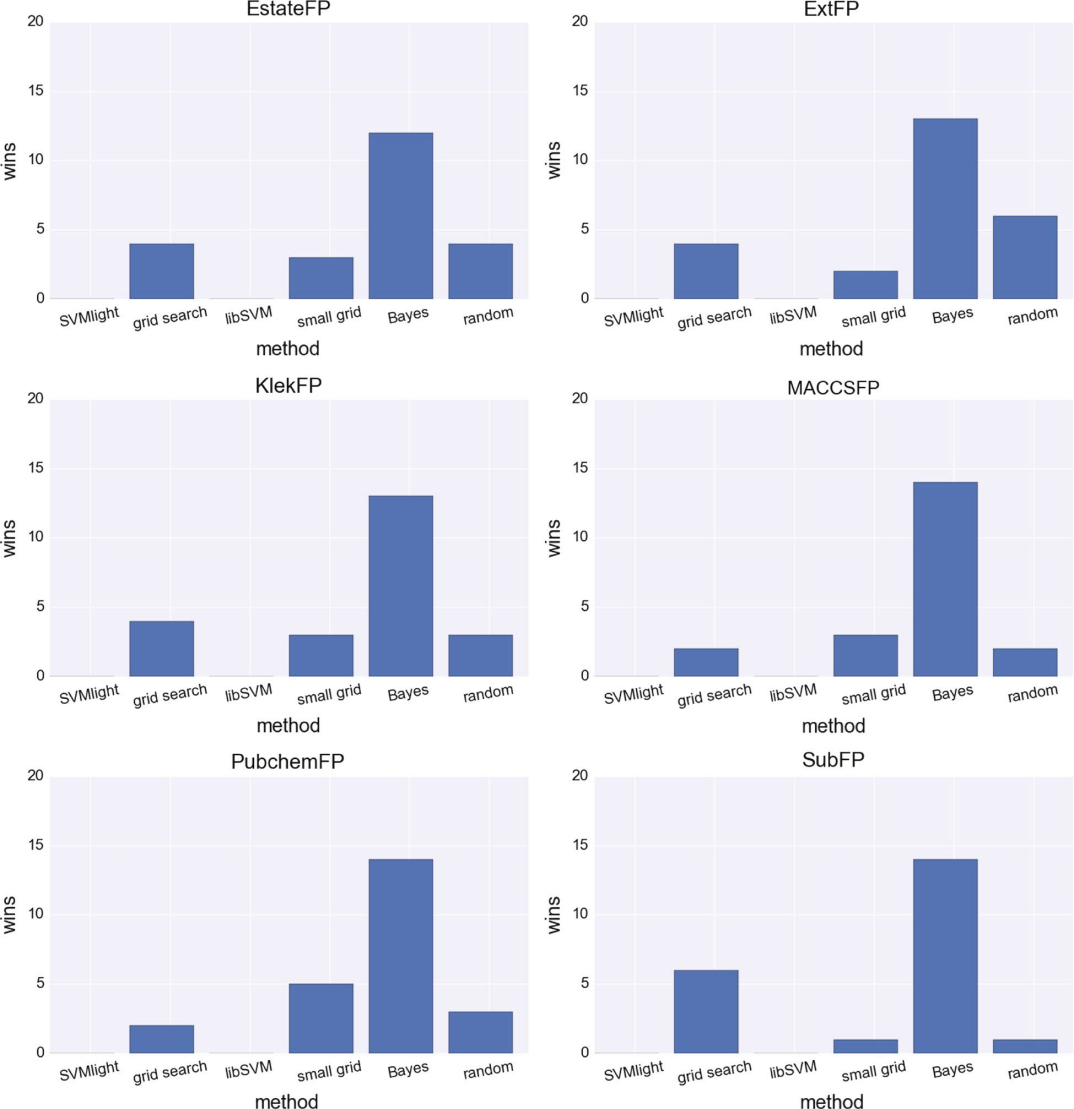
Analysis of the effectiveness of different SVM optimization strategies with respect to various fingerprints expressed as the number of experiments in which a particular strategy provided the highest accuracy values for a given compounds representation.

The relationships between various methods tested were preserved when the results were analyzed with respect to the various fingerprints (Fig. 2)—the Bayesian optimization always provided the highest classification accuracy (for 13–14 targets for each of the fingerprints analyzed), whereas the ‘global’ second-place method—grid search—was outperformed by ‘small grid’-cv for two fingerprints: MACCSFP and PubchemFP. The runners-up (grid search or ‘small grid’-cv, depending on fingerprint) provided the best predictive power of the model for 3 proteins on average. The ineffectiveness of the SVMlight and libSVM strategies has been already indicated in the 'global' analysis, and with respect to various fingerprints, there was no protein for which those SVM optimization methods provided the highest classification accuracy.

**Fig. 3:**
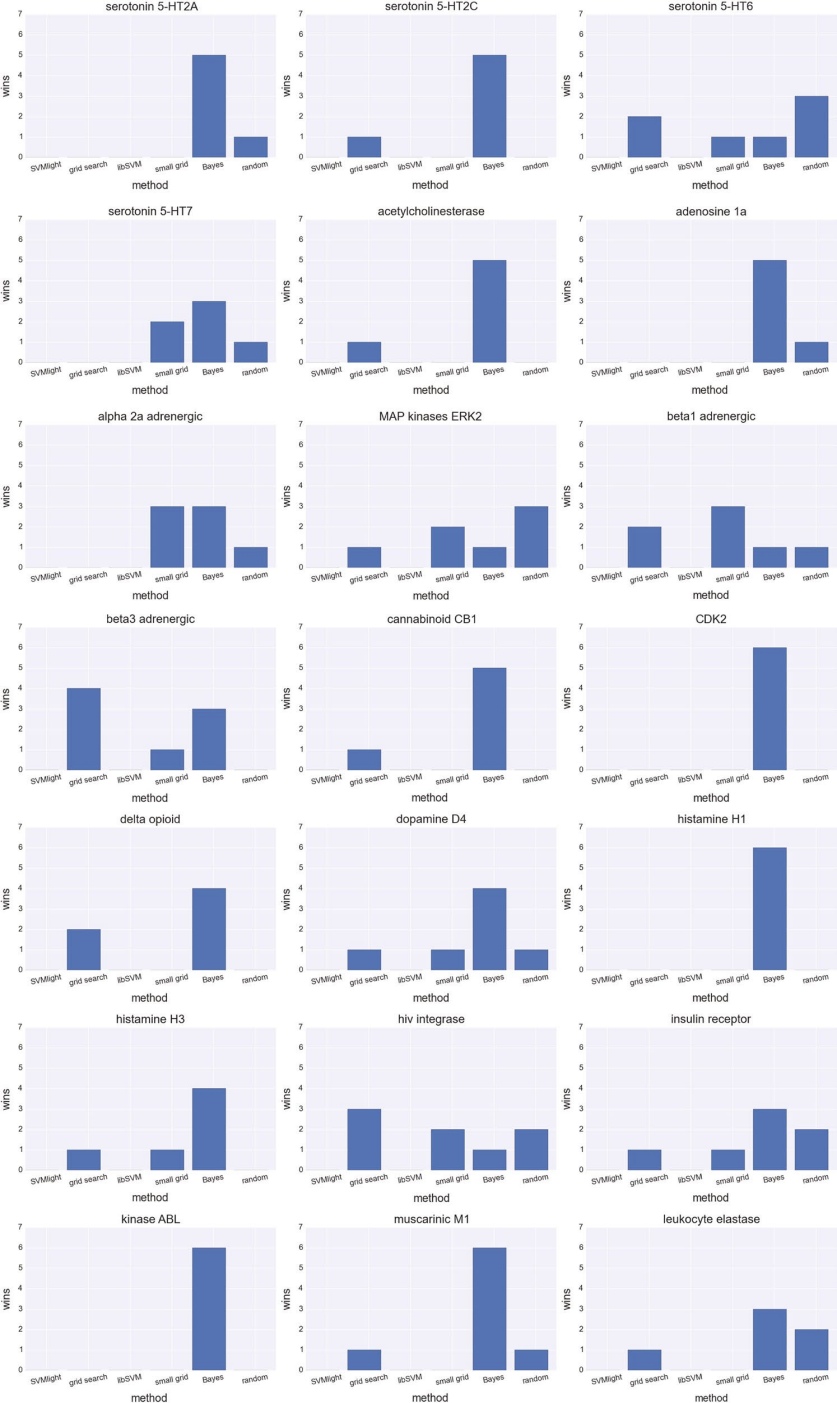
Analysis of effectiveness of different SVM optimization strategies with respect to various targets expressed as the number of experiments in which a particular strategy provided the highest accuracy values for a given protein target.

The situation becomes more complex when separate targets are taken into account (Fig. 3). The Bayesian optimization provided the best results for all considered representations for some proteins (CDK2, H_1_, ABL); however, in few cases, other optimization approaches for tuning SVM parameters outperformed the Bayesian method (5-HT_6_—random and grid search, beta1AR—‘small grid’-cv and grid search, beta3AR—grid search, HIVi—grid search, ‘small grid’-cv, random, MAP kinases ERK2—‘small grid’-cv and random search). These results show that a more careful model accuracy approximation is required for some proteins. Because we are interested in maximizing the accuracy on a naive test set, we approximate this set by performing internal cross-validation for each method. This is a well-known technique in ML; however, it might be *not reliable* for small datasets. Beta1AR, beta3AR, and HIVi are very small datasets in our comparison; thus, it seems probable that the poor results of the Bayesian approach (a poor approximation of the $\mathcal {S}$ value) were caused by the high internal variance in the dataset rather than because the Bayesian approach was actually worse than the grid search method.

**Table 2:** A comparison of the number of highest accuracies obtained with the Bayesian optimization and grid search

Comparison	Bayes	Grid search
Global	96	34
EstateFP	15	7
ExtFP	16	6
KlekFP	16	5
MACCSFP	18	4
PubchemFP	16	6
SubFP	15	6
5-HT$_\text {2A}$	5	1
5-HT$_\text {2C}$	5	1
5-HT$_\text {6}$	4	3
5-HT$_\text {7}$	3	3
CDK2	6	0
M$_\text {1}$	6	1
ERK2	5	1
AChE	5	1
A$_\text {1}$	5	1
alpha2AR	5	1
beta1AR	3	3
beta3AR	3	4
CB1	5	1
DOR	4	2
D$_\text {4}$	5	1
H$_\text {1}$	6	0
H$_\text {3}$	5	1
HIVi	1	5
IR	5	1
ABL	6	0
HLE	4	3

Because grid search was the second-place method in the majority of the analyses, both for global analysis, and fingerprint- and target-based comparisons, a direct comparison of the number of the highest accuracies obtained for Bayesian optimization and the grid search approach was performed (Table 2). The sum of the number of wins is not equal for the given fingerprint-based or target-based comparison as the draws were also considered.

The comparison of the number of 'wins' for Bayesian optimization over the grid search indicates the superiority of the former approach. In the ‘global’ analysis, the Bayesian optimization strategy gave a higher accuracy for approximately a 3-fold higher number of experiments than the grid search. For the fingerprint-based analysis, the ratio of Bayesian/grid search wins was similar to the best ratio (in favor of Bayesian optimization) obtained for MACCSFP (18 : 4) and the worst (15 : 7 and 15 : 6) for EstateFP and SubFP, respectively. When target-based comparisons were considered, Bayesian optimization outperformed the grid search approach for some targets in all cases (i.e., CDK2, H$_\text {1}$, ABL); for others, there was only 1 case when the grid search strategy won (i.e., 5-HT$_\text {2A}$, 5-HT$_\text {2C}$, M$_\text {1}$, ERK2, AChE, A$_\text {1}$, alpha2AR, CB1, D$_\text {4}$, H$_\text {3}$, IR), still others were draws (i.e., 5-HT$_\text {7}$, beta1AR), and in two cases the grid search provided top accuracies (beta3AR, HIVi).

**Fig. 4:**
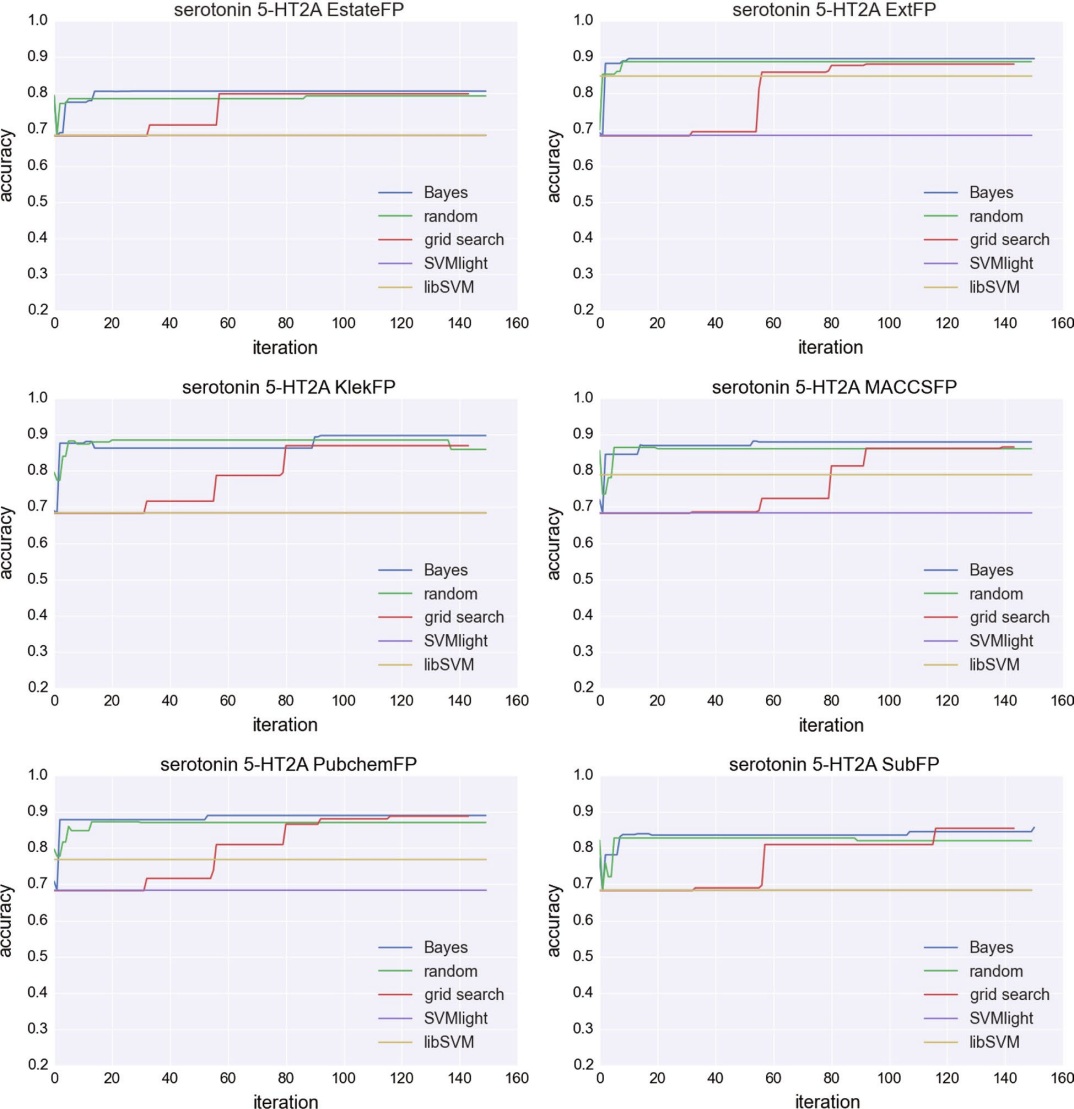
Analysis of the changes in accuracy during the SVM optimization procedure for the subsequent optimization steps.

### Examination of optimization steps in time

A time course study of the accuracy values was also conducted. Figure 4 shows analyses for 5-HT$_\text {2A}$ as an example; the results for the remaining targets are in the Additional files section (Additional file 1). We demonstrated not only that Bayesian optimization required the smallest number of iterations to achieve optimal performance (for all representations the number of iterations was less than 20), but also that in the majority of cases, SVM optimized using a Bayesian approach achieved better performance than all of the other optimization methods. SVMlight and libSVM were not iteratively optimized; therefore, the accuracy/number of iterations function is constant for these approaches. In general, the Bayesian and random search approach were optimized very quickly (in less than 20 iterations), whereas the grid search method required many more iterations before the SVM reached optimal performance: 57 iterations (the lowest number) were required for EstateFP, and 138 (the highest number) for MACCSFP). Figure 4 also shows the rate of the improvement of the accuracy after the application of particular optimization approach, which depended on the representation of the compounds—for EstateFP, it was improvement from 0.8 (grid search) up to 0.82 (Bayesian), but for libSVM and SVMlight the improvement was significantly higher; for these two strategies, the classification accuracy was equal to 0.68. A similar result was obtained for ExtFP—the rate of improvement for the Bayesian optimization strategy compared with the random search approach was approximately 0.02 (from 0.88 to 0.90), but it was higher for the other optimization methods: 0.03 for grid search, 0.05 for libSVM and 0.22 for SVMlight. The pattern was similar for KlekFP and MACCSFP, with differences occurring only in the performance of libSVM. However, for PubchemFP and SubFP, grid search optimization provided the same predictive power for SVM as Bayesian optimization; for the SubFP, there was a selected range of iterations (117–142) when grid search provided slightly better SVM performance (by about 2%) in comparison to Bayesian approach.

**Table 3:** The AUC values obtained in 5-HT$_\text {2A}$, ExtFP for curves illustrating changes in the accuracy in time and final optimal accuracy values obtained

optimization method	AUC	Final accuracy
Bayes	0.892*	0.896*
Random	0.885	0.887
Grid search	0.802	0.881
SVMlight	0.683	0.683
libSVM	0.847	0.847

The highest values obtained among all strategies tested are marked with an asterisk sign

In order to provide the comprehensive and global analysis of the changes in accuracy with an increasing number of iterations, the areas under curves (AUC) presented in Fig. 4 (and other curves that are placed in the Additional files section) were calculated. Example analysis for selected target/fingerprint pair (5-HT$_\text {2A}$, ExtFP) is presented in Table 3; the remaining analyses are in the Additional files section (Additional file 2). The global average AUC, the average AUC for particular fingerprints and targets are presented in Tables  4 and  5. The Tables also include final (for the selected target/fingerprint) and averaged (for the rest of the cases) final accuracy values obtained for a given strategy; the highest AUC/accuracy values for the particular case considered are marked with an asterisk sign. In general, the AUC of a curve indicates the strength of the trained model at any randomly chosen iteration. In other words, the AUC measures how quickly a given strategy converges to a strong model.

**Fig. 5:**
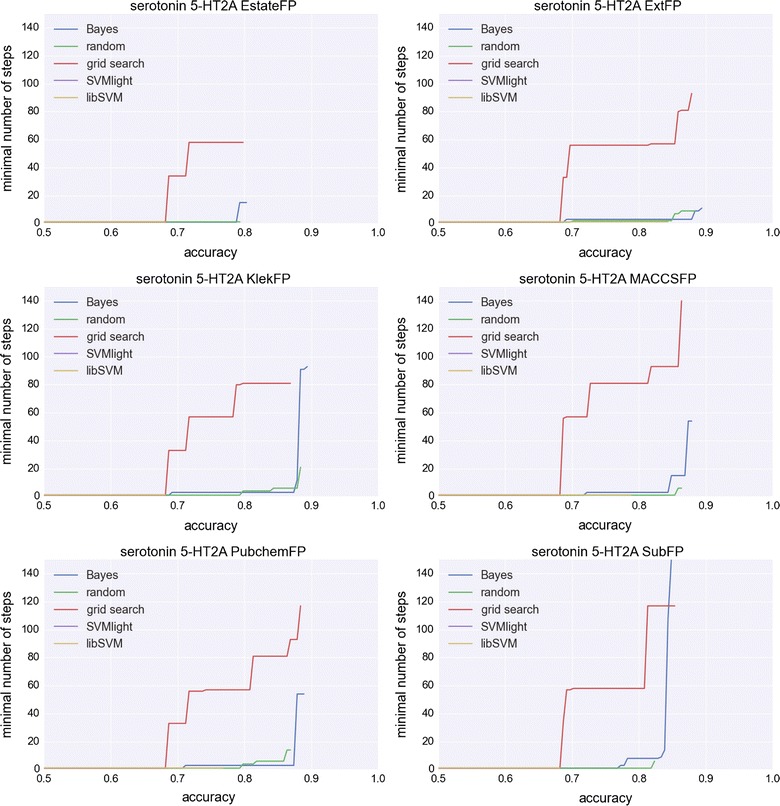
Analysis of the number of iterations of the optimization procedure required to achieve the highest accuracy. The figure presents the number of iterations required for a particular optimization strategy to achieve optimal performance for the predictive model.

**Table 4:** The average AUC values–global, obtained for a particular fingerprint and particular target

Fingerprint/target	Bayes	Random	Grid search	SVMlight	libSVM
global	0.883*	0.870	0.799	0.676	0.792
EstateFP	0.847*	0.829	0.774	0.690	0.763
ExtFP	0.902*	0.891	0.806	0.669	0.874
KlekFP	0.899*	0.889	0.812	0.669	0.730
MACCSFP	0.890*	0.876	0.798	0.683	0.828
PubchemFP	0.898*	0.885	0.816	0.669	0.808
SubFP	0.864*	0.854	0.787	0.677	0.749
5-HT$_\text {2A}$	0.860*	0.850	0.780	0.683	0.743
5-HT$_\text {2C}$	0.848*	0.821	0.702	0.568	0.717
5-HT$_\text {6}$	0.913*	0.910	0.886	0.814	0.862
5-HT$_\text {7}$	0.830*	0.816	0.748	0.675	0.714
CDK2	0.876*	0.875	0.796	0.664	0.768
M$_\text {1}$	0.850*	0.843	0.778	0.557	0.748
ERK2	0.958	0.961*	0.949	0.931	0.942
AChE	0.884*	0.854	0.788	0.611	0.764
A$_\text {1}$	0.843*	0.835	0.764	0.564	0.720
alpha2AR	0.875*	0.874	0.773	0.563	0.725
beta1AR	0.910*	0.864	0.798	0.710	0.828
beta3AR	0.874*	0.823	0.826	0.545	0.722
CB1	0.874*	0.854	0.782	0.622	0.793
DOR	0.888*	0.880	0.734	0.599	0.814
D$_\text {4}$	0.841*	0.837	0.759	0.698	0.745
H$_\text {1}$	0.898*	0.880	0.638	0.548	0.801
H$_\text {3}$	0.937*	0.926	0.906	0.897	0.905
HIVi	0.939	0.945*	0.934	0.901	0.911
IR	0.936*	0.936*	0.925	0.886	0.897
ABL	0.850*	0.831	0.748	0.587	0.733
HLE	0.867*	0.865	0.763	0.578	0.779

The highest values obtained among all strategies tested are marked with an asterisk sign

**Table 5:** The average final accuracy values—global, obtained for a particular fingerprint and particular target

fingerprint/target	Bayes	Random	Grid search	SVMlight	libSVM
Global	0.889*	0.873	0.876	0.676	0.792
EstateFP	0.852*	0.832	0.833	0.690	0.763
ExtFP	0.907*	0.896	0.892	0.669	0.874
KlekFP	0.907*	0.890	0.891	0.669	0.730
MACCSFP	0.898*	0.878	0.880	0.683	0.828
PubchemFP	0.901*	0.886	0.894	0.669	0.808
SubFP	0.869*	0.856	0.864	0.677	0.749
5-HT$_\text {2A}$	0.871*	0.848	0.860	0.683	0.743
5-HT$_\text {2C}$	0.855*	0.825	0.772	0.568	0.717
5-HT$_\text {6}$	0.916*	0.915	0.933	0.814	0.862
5-HT$_\text {7}$	0.833*	0.819	0.819	0.675	0.714
CDK2	0.885*	0.881	0.870	0.664	0.768
M$_\text{1}$	0.858	0.846	0.897*	0.557	0.748
ERK2	0.959	0.961*	0.961*	0.931	0.942
AChE	0.889*	0.857	0.872	0.611	0.764
A$_\text {1}$	0.856	0.838	0.882*	0.564	0.720
alpha2AR	0.880*	0.873	0.872	0.563	0.725
beta1AR	0.914*	0.870	0.864	0.710	0.828
beta3AR	0.879	0.825	0.972*	0.545	0.722
CB1	0.881*	0.857	0.868	0.622	0.793
DOR	0.897*	0.884	0.872	0.599	0.814
D$_\text {4}$	0.849*	0.838	0.837	0.698	0.745
H$_\text {1}$	0.904*	0.879	0.691	0.548	0.801
H$_\text {3}$	0.938*	0.926	0.919	0.897	0.905
HIVi	0.938	0.946	0.967*	0.901	0.911
IR	0.939	0.937	0.956*	0.886	0.897
ABL	0.857*	0.836	0.840	0.587	0.733
HLE	0.867*	0.871	0.864	0.578	0.779

The highest values obtained among all strategies tested are marked with an asterisk sign

The analysis of the results obtained for the example target/fingerprint pair (5-HT$_\text {2A}$, ExtFP; Table 3) shows that both the highest AUC and final optimal accuracy values were obtained with the Bayesian strategy for SVM optimization. A similar observation was made for the global and fingerprint-based analysis; Bayesian optimization provided the best average AUC and average optimal accuracy for all fingerprints, as well as the global average value of this parameter. Interestingly, although grid search was the second-place method for optimal accuracy, it was actually the random search that outperformed this method in terms of AUC, which could be explained from an analysis of the respective curves. Although the grid search method provided higher final accuracy values, these occurred relatively 'late' (after a series of iterations), high accuracies were obtained almost immediately for random search (Figs. 4, 5). Similarly, the average AUC and optimal accuracy values calculated for various targets were highest for Bayesian optimization in the great majority of cases. HIVi and ERK2 were the only targets for which the averaged AUC obtained with the Bayesian optimization strategy was outperformed by other optimization methods. On the other hand, the group of targets for which the average optimal accuracy values were the highest for methods other than Bayesian optimization was a bit more extensive (i.e., M$_\text {1}$, ERK2, A$_\text {1}$, beta3AR, HIVi, IR). However, for most of these targets, the difference between the best average accuracy and that obtained with Bayesian optimization was approximately 3% (however, for example for beta3AR this difference approached to 10%, from 0.879 to 0.972). On the other hand, an improvement of several percentage points was also observed when the average AUC and optimal accuracy obtained with the Bayesian strategy were compared with the strategy that provided the ‘second-best’ accuracy value in the ranking.

The number of iterations required to achieve optimal SVM performance was also analyzed in detail (Fig. 5; Additional file 3). The most striking observation was that all curves corresponding to the Bayesian optimization results were both shifted towards higher accuracy values and were much ‘shorter’, meaning that a significantly lower number of iterations was necessary in total to reach optimal SVM performance. Two relevant points arise from a comparison of Bayesian optimization with the grid search method (which sometimes outperformed Bayesian optimization): obtaining optimal accuracy with the grid search method required many more calculations, and even when grid search yielded higher accuracy values than Bayesian optimization, the difference between the two was approximately 1–2%. This result indicates that even when Bayesian optimization ‘lost’, the results provided by this strategy were still very good and taking into account the calculation speed, it can be successfully applied also in experiments for which it was not indicated to be the best approach. A very interesting observation arising from Fig. 5 is that random search reached the optimal classification effectiveness (as measured by accuracy) in the least number of iterations, below 10 in the majority of cases. EstateFP, ExtFP, MACCSFP and PubchemFP, showed similar tendency with respect to the comparison of Bayesian optimization and the grid search strategy; for an initial number of iterations (40), the accuracy values obtained with the grid search were approximately 20% lower than those obtained with the Bayesian approach. However, as the number of iterations for grid search increased, the accuracy values were also higher, and when the number of iterations reached approximately 100, the grid search results were similar to those obtained with Bayesian optimization. On the other hand, for both KlekFP and SubFP, the initial observations were the same; for a lower number of iterations, Bayesian optimization led to significantly higher accuracy values than the grid search approach, and for a higher number of iterations (over 80 for KlekFP and over 115 for SubFP), grid search provided accuracy values at a similar level to the values obtained with the Bayesian strategy. However, increasing the number of iterations for Bayesian optimization from approximately 10 to 90 for KlekFP and 150 for SubFP did not lead to a significant increase in the accuracy (an almost vertical line corresponding to these numbers of iterations), which was already very high (over 0.85 for KlekFP and over 0.8 for SubFP). Further optimization led to further improvement in accuracy of approximately 2–3%.

**Fig. 6:**
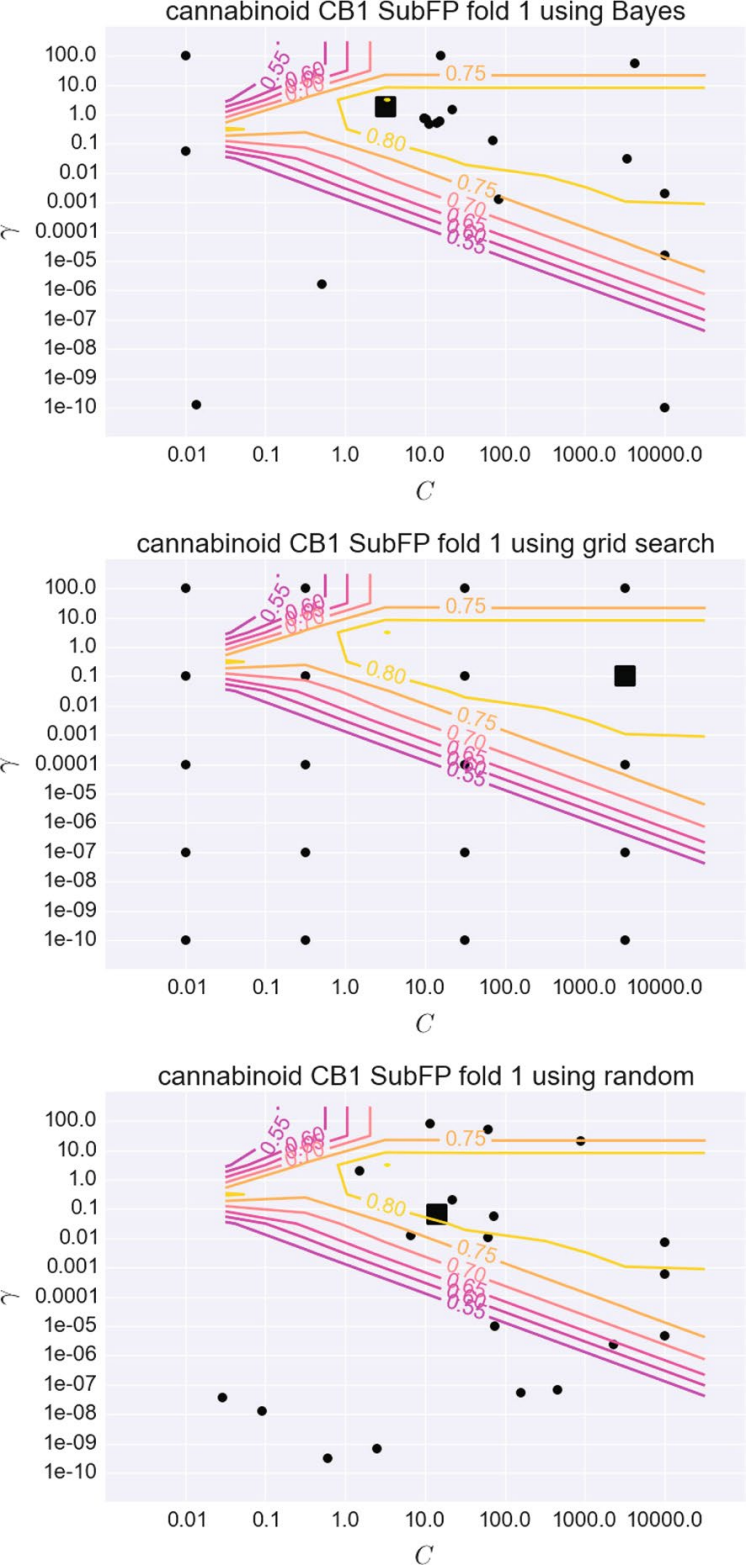
Analysis of the changes in accuracy for different steps during the SVM optimization procedure.

The results were also analyzed regarding the changes in the accuracy when additional steps were applied. A panel of example results is shown in Fig. 6 for the cannabinoid CB1/SubFP combination (the remaining targets are in Additional file 4). The black dots show the set of parameters tested in the particular approach, and the black squares represent the set of parameters selected as optimal. This chart shows the advantage of Bayesian optimization in terms of the way of work, and the sequence of selected parameters. The set of tested parameters is fixed for grid search optimization, whereas in case of random search, it is based on the random selection. On the other hand, the selection of parameters for Bayesian optimization is more directed, which also affects the effectiveness of the classification. For grid search, only a small fraction of the parameters tested provided satisfactory predictive power of the model (only approximately 35% of the predictions resulted in an accuracy exceeding 0.7). Surprisingly, a relatively high classification efficiency was obtained with the use of the random search approach—60% of the sets of parameters tested provided predictions with an accuracy over 0.7. However, investigation of the Bayesian optimization approach to parameter selection revealed that the choice of parameters tested was *justified*, and hence, the results obtained with their use were significantly better than those obtained with the other approaches—75% predictions with accuracy over 0.7.

We conclude that there are three SVM hyperparameters selection approaches worth using for activity prediction for compounds:

- libSVM heuristic (when only one set of hyperparameters is needed),
- random search (when we need a strong model quickly, using less than a few dozen iterations),
- a Bayesian approach (when we want the strongest model and can wait a bit longer).

The SVMlight heuristic as well as the traditional grid search approach have definitely been shown to be significantly worse in terms of the resulting model accuracy as well as time needed to construct such model.

**Table 6:** The number of active and inactive compounds in the dataset

Protein	Actives	Inactives
5-HT$_\text {2A}$	1836	852
5-HT$_\text {2C}$	1211	927
5-HT$_\text {6}$	1491	342
5-HT$_\text {7}$	705	340
CDK2	741	1462
M$_\text {1}$	760	939
ERK2	72	958
AChE	1147	1804
A$_\text {1}$	1789	2286
alpha2AR	364	283
beta1AR	195	477
beta3AR	111	133
CB1	1964	1714
DOR	2535	1992
D$_\text {4}$	1034	449
H$_\text {1}$	636	546
H$_\text {3}$	2706	313
HIVi	102	915
IR	147	1139
ABL	409	582
HLE	820	610

## Experimental

Several compounds datasets were prepared and their proper description using various fingerprints was provided for the planned experiments. The ChEMBL database constituted a source of active and inactive compounds with experimentally verified activity towards selected targets. The following proteins were considered in the study: serotonin receptors 5-HT$_\text {2A}$ [11], 5-HT$_\text {2C}$ [12], 5-HT$_\text {6}$ [13], and 5-HT$_\text {7}$ [14], cyclin dependent kinase 2 (CDK2) [15], muscarinic receptor M$_\text {1}$ [16], MAP kinase ERK2 [17], acetylcholinesterase (AChE) [18], adenosine receptor A$_\text {1}$ [19], alpha-2A adrenergic receptor [20], beta-1 adrenergic receptor (beta1AR) [21], beta-3 adrenergic receptor (beta3AR) [21], cannabinoid CB1 receptor [22], delta opioid receptor (DOR) [23], dopamine receptor D$_\text {4}$ [24], histamine receptor H$_\text {1}$ [25], histamine receptor H$_\text {3}$ [26], HIV integrase (HIVi) [27], insulin receptor (IR) [28], tyrosine kinase ABL [29], and human leukocyte elastase (HLE) [30]. Only molecules whose activities were quantified in $K_{i}$ or $IC_{50}$ and that were tested in assays on human, rat-cloned or native receptors were taken into account. The compounds were considered active when the median value of all $K_{i}$ values provided for a particular instance was lower than 100 nM, and inactive when the median $K_{i}$ value was greater than 1000 nM. The number of compounds from each group for the selected targets is shown in Table 6. The following fingerprints were used for compounds representation: E-state Fingerprint (EstateFP) [31], Extended Fingerprint (ExtFP) [32], Klekota and Roth Fingerprint (KlekFP) [33], MACCS Fingerprints (MACCSFP) [34], Pubchem Fingerprint (PubchemFP), and Substructure Fingerprint (SubFP), generated with the use of the PaDEL-Descriptor [35]. A brief characterization of the fingerprints is provided in Table 7).

**Table 7:** Fingerprints used for compounds representation

Fingerprint	Abbreviation	Length	Short description
E-State fingerprint	EStateFP	79	Computes electrotopological state (E-state) index for each atom, describing its electronic state with consideration of the influence of other atoms in particular structure
Extended fingerprint	ExtFP	1024	A hashed fingerprint with each atom in the given structure being a starting point of a string of a length not exceeding six atoms. A hash code is produced for every path of such type and in turn it constitutes the basis of a bit string representing the whole structure
Klekota and Roth fingerprint	KlekFP	4860	Fingerprint analyzing the occurrence of particular chemical substructures in the given compound. Developed by Klekota and Roth
MACCS fingerprint	MACCSFP	166	Fingerprint using the MACCS keys in its bits definition
Pubchem fingerprint	PubchemFP	881	Substructure fingerprint with bits divided into several sections: hierarchic element counts, rings, simple atom pairs, simple atom nearest neighbours, detailed atom neighbourhoods, simple SMART patterns, complex SMART patterns
Substructure fingerprint	SubFP	308	Substructure fingerprint based on the SMART patterns developed by Christian Laggner

The following SVM strategies were used:

- default SVM parameters used in the WEKA package ($C = 1, \gamma = \tfrac{1}{d}$)—libSVM.
- default SVM parameters from the SVMlight library ($C = \frac{1}{\mathrm {\underset{i}{mean}} \Vert x_{i}\Vert ^2},\; \gamma = \tfrac{1}{d}$).
- grid search optimization of SVM parameters—$\log _{10}(C) \in [-2, 5]$, $\log _{10}(\gamma ) \in [-10, 3]$.
- SVM parameters optimization in the truncated cross-validation mode (‘small grid’-cv).
- SVM parameters optimization in the random cross-validation mode—number of iterations: up to 150.
- Bayesian optimization using BayesOpt [36]—number of iterations: up to 150.

The range of *C* and $\gamma$ values tested was as follows: $\log _{10}(C) \in [-2, 5]$, $\log _{10}(\gamma ) \in [-10, 3]$ (the result of preliminary grid search experiments). The number of iterations in which random search, 'small grid'-cv and Bayesian optimization experiments were performed fell within the following set: 20, 30, 50, 75, 100, 150.

The predictive power of SVM for different optimization strategies applied was measured by the accuracy:

$$\begin{aligned} \mathrm {Accuracy} (TP, FP, TN, FN)= \frac{\mathrm {TP + TN}}{\mathrm {TP + TN + FP + FN}}, \end{aligned}$$

with TP being the number of true positives (correctly classified actives), TN—the number of true negatives (correctly classified inactives), FP—the number of false positives (inactives wrongly classified as active), and FN—the number of false negatives (actives wrongly classified as inactive).

## Conclusions

The paper presents strengths of Bayesian optimization applied for fitting SVM hyperparameters in cheminformatics tasks. Because the importance and necessity of the SVM optimization procedure is undeniable, various approaches to this task have neen developed so far. However, the most popular approaches to SVM optimization are not always very effective, in terms of both the predictive power of the models obtained and the computational requirements. This study demonstrated that Bayesian optimization not only provides better classification accuracy than the other optimization approaches tested but is also much faster and directed—in the majority of cases, the number of iterations required to achieve optimal performance was the lowest out of the all methods tested, and the set of parameters tested provided the best predictions on average. Interestingly, if good classification results are desired to be obtained quickly (using a low number of iterations and without complex algorithms), the random search method in which hyperparameters are randomly selected from a predefined range) leads to very good performance of the SVM for predicting the activity of compounds and can thus be used when Bayesian optimization approach is not feasible.

Consequently, we can formulate the following rule of thumb for tuning SVM’s hyperparameters for the classification of bioactive compounds:

1. If you have no resources for performing hyperparameters optimization, use $C=1, \gamma = \frac{1}{d}$ (as defined in libSVM).
2. If you have limited resources (up to 20 learning procedures) or limited access to complex optimization software, use a random search for *C* and $\gamma$ with distribution defined in the “Methods” section.
3. If you have resources for 20 or more training runs and access to Bayesian optimization software^a^, use a Bayesian optimization of $C, \gamma$.

In general, there is no scenario in which one should use a grid search approach (it is always preferable to use random search or a Bayesian method) or SVMlight heuristics (it is always better to use libSVM) in the tasks connected with the assessment of compounds bioactivity.

## Methods

The objective of the iterative global optimization of a function $f : \mathcal {L} \rightarrow \mathbb {R}$ is to find the sequence of points

$$\begin{aligned} \varvec{\lambda }_1, \dots , \varvec{\lambda }_n \in \mathcal {L}, \end{aligned}$$

that converges to the optimal $\hat{\varvec{\lambda }}$, $f(\hat{\varvec{\lambda }}) = \sup _{\varvec{\lambda }\in \mathcal {L}} f(\varvec{\lambda })$. A good algorithm should find a solution at least over some family of functions $\mathcal {F}$, not necessarily containing *f*.

The above-mentioned issue can be viewed as a sequential decision making problem [37] in which at time step *i* a decision based on all previous points $\alpha _i(\varvec{\lambda }_{1:i-1}, \bar{f}_{1:i-1})$, where $\bar{f}_i = f(x_i) + \varepsilon _i$ is made. In other words, we have access to approximations of *f* values from previous steps. For simplicity, assume that $\varepsilon _i = 0$ (*f* is deterministic); however, in general, all methods considered can be used in a stochastic scenario (for example, when randomized cross-validation is used as underlying method for *f* evaluation).

The goal is to find $\alpha$ which minimizes $\delta _n(\alpha ) = f(\hat{\varvec{\lambda }}) - f(\varvec{\lambda }_n)$, meaning that we are interested in

1 $$\begin{aligned} \arg \min _\alpha \delta _n(\alpha ) = \arg \min _\alpha f(\hat{\varvec{\lambda }}) - f(\varvec{\lambda }_n), \end{aligned}$$

which could be efficiently solved if *f* is known.

### Approximation of generalization capabilities

In general, we are interested in how well our predictive model behaves on a naive test set. In other words, we are assuming that our data are a finite iid (independent and identically distributed) sample from some underlying joint distribution over samples (compounds) and their binary labels (biological activity) $\mu$:

$$\begin{aligned} \mathrm {T}=\{(x_i, y_i)_{i=1}^N\} \sim \mu ( \mathcal {X} \times \{-1, +1\}), \end{aligned}$$

where $\mathcal {X}$ represents a feature space of compounds under investigation. We want to maximize the *expected accuracy* over all possible compounds from $\mu$, in other words

$$\begin{aligned} f(\varvec{\lambda }) &= \mathbb {E}_{(x_i,y_i) \sim \mu }[\mathrm {svm}(x_i|\mathrm {T},\varvec{\lambda })=y_i]\\ &= \int [\mathrm {svm}(x_i|\mathrm {T},\varvec{\lambda })=y_i] d\mu , \end{aligned}$$

where [ *p* ] is a characteristic function returning 1 if and only if *p* is true, and $\mathrm {svm}(x_i|\mathrm {T}, \varvec{\lambda })$ is a prediction of $x_i$’s label by SVM trained with hyperparameters $\varvec{\lambda }$ on training set $\mathrm {T}$.

Clearly, we cannot integrate over an unknown probability distribution, but we can approximate this value using internal cross-validation. In other words, we are using a stochastic approximation

$$\begin{aligned} \bar{f}(\varvec{\lambda }) = \text {CV}_\mathrm {T}(\mathrm {svm}(X_\mathrm {test}|\mathrm {T}_\mathrm {train},\varvec{\lambda }), Y_\mathrm {test}), \end{aligned}$$

where $\mathrm {CV}_\mathrm {T}(p, y)$ is the mean accuracy of the model of predictions *p* as compared to the true labels *y* over splits of set $\mathrm {T}$ into $\mathrm {T}_\mathrm {train}$ and $\mathrm {T}_\mathrm {test}$ (composed of $X_\mathrm {test}$ data and corresponding labels $Y_\mathrm {test}$). Thus we can assume [38] that

$$\begin{aligned} \bar{f}(\varvec{\lambda }) = f(\varvec{\lambda }) + \varepsilon , \end{aligned}$$

where $\varepsilon$ is a random noise variable (resulting from the approximating error and stochastic nature of cross validation).

### Random optimization

First, let us define a random optimization technique as a strategy $\alpha ^\mathcal {R}(\varvec{\lambda }_{1:i-1}, \bar{f}_{1:i-1}) = \alpha ^\mathcal {R}_i = \varvec{\lambda }_i \sim \varvec{P}(\mathcal {L})$, for some probability distribution over the hyperparameters $\varvec{P}(\mathcal {L})$. In other words, in each iteration, we sample from $\varvec{P}(\mathcal {L})$, ignoring all previous samples and their results. Finally, we return the maximum of the values obtained.

It is easily seen that a random search, under the assumption that $\forall _{\varvec{\lambda }\in \mathcal {L}} \varvec{P}(\alpha ^\mathcal {R}_i = \varvec{\lambda }) > 0$, has a property described in (1). A random search will converge to the optimum [39], if only each set of parameters is possible to generate when taking new sample from our decision making process. In practise, it is only necessary that $\varvec{P}(f(\alpha ^\mathcal {R}_i) = f(\hat{\varvec{\lambda }})) > 0$. Similarly, if one uses a grid search approach that discretizes $\mathcal {L}$, then given enough iterations and the assumption that *f* is continuous, one will converge to the optimal solution. It is important to note that the speed of such a convergence can be extremely low.

The only thing missing is the selection of $\varvec{P}(\mathcal {L})$. According to many empirical studies showing that meaningful changes in the SVM results as the function of its hyperparameters can be expressed in log-scale of these parameters we use

$$\begin{aligned} \varvec{P}(\varvec{\lambda }= (C, \gamma )) = \tfrac{\log _{10} C - \log _{10} C_\mathrm {min}}{\log _{10} C_\mathrm {max} - \log _{10} C_\mathrm {min}} \cdot \tfrac{\log _{10}\gamma - \log _{10}\gamma _\mathrm {min}}{\log _{10}\gamma _\mathrm {max} - \log _{10}\gamma _\mathrm {min}} \end{aligned}$$

where we are interested in $\mathcal {L} = [C_\mathrm {min}, C_\mathrm {max}] \times [\gamma _\mathrm {min}, \gamma _\mathrm {max}]$. In other words, we are using a log-uniform distributions independently over *C* and $\gamma$.

### Grid search

For grid search optimization we select $\varvec{\lambda }$ in a similar manner to the random search approach, uniformly in the log-scale of the parameters, and given $M_p$ choices of parameter *p*

$$\begin{aligned} \varvec{\lambda }_{ij}&= (C_i, \gamma _j) \\&= \left( 10^{\log _{10} C_\mathrm {min} + (i-1) \tfrac{\log _{10} C_\mathrm {max} - \log _{10} C_\mathrm {min}}{M_C-1}}, 10^{\log _{10} \gamma _\mathrm {min} + (j-1) \tfrac{\log _{10} \gamma _\mathrm {max} - \log _{10} \gamma _\mathrm {min}}{M_\gamma -1}} \right) . \end{aligned}$$

We put the linear order of $\varvec{\lambda }_{ij}$ by raveling the resulting matrix by column, which is the most common practice in most ML libraries. It is worth noting that one could achieve better scores by alternating this ordering to any random permutation; however, in practice, such alternation is rarely performed.

### Bayesian optimization

If the exact form of *f* is known (for example, if *f* is convex and its derivative is known), then the optimization procedure would be much simpler. Unfortunately, *f* is a black-box function wih a very complex structure, expensive even to evaluate. However, some simplifying assumptions for *f* might make a problem solvable. Assume that *f* can be represented as a sample from a probability distribution over a family of functions $f \sim \varvec{P}(f), f \in \mathcal {F}$.

We can now express the expectation over the loss function $\delta _n$:

$$\begin{aligned} \arg \min _\alpha \mathbb {E}_{\varvec{P}(f)} [\delta _n(\alpha )] = \arg \min _\alpha \int _{\mathcal {F}} \delta _n(\alpha ) d \varvec{P}(f). \end{aligned}$$

Given that in step *n* the values of $\varvec{\lambda }_i, \bar{f}_i$ for $i=1, \dots , n-1$ are already known and using the Bayes rule, we can write:

$$\begin{aligned} \varvec{P}(f | x_{1:i}, \bar{f}_{1:i}) &= \frac{\varvec{P}(x_i, \bar{f}_i | f) \varvec{P}(f|x_{1:i-1}, \bar{f}_{1:i-1})}{\varvec{P}(x_i, \bar{f}_i)},\\ &\quad\quad\forall i = 1, \dots , n-1, \end{aligned}$$

thus

$$\begin{aligned}& \arg \min _\alpha \mathbb {E}_{\varvec{P}(f|x_{1:n-1}, \bar{f}_{1:n-1})} [\delta _n(\alpha )] \\ &\quad = \arg \min _\alpha \int _{\mathcal {F}} \delta _n(\alpha ) d \varvec{P}(f|x_{1:n-1}, \bar{f}_{1:n-1}). \end{aligned}$$

This is a very basic equation for general Bayesian optimization techniques. Given additional assumptions about the prior distribution of $\varvec{P}(f)$, very efficient solutions for the entire process can be provided. In the case considered here, a very common approach exploiting features of the Gaussian processes is employed; thus, we assume that our target function (the generalization capabilities of the SVM with RBF kernel applied to the prediction of the activity of chemical compounds) *f* is a sample of the stochastic process. A crucial advantage of such a simplification is that we can easily manipulate the distribution over such functions: in particular, the posterior distribution is also a Gaussian process. Consequently, in each iteration a calculated posterior can be used as an informative prior for the next iteration, creating a relatively simple iterative procedure.

The only thing missing is the selection of the loss function, because $\delta _n(\alpha )$ defined above requires knowledge of the optimal solution. There are many surrogate functions (also called proxies) that might be of use. In our investigations we used one of the most well-known [7] and studied *expected improvement* [40, 41], which has the following closed form solution under the above assumptions:

$$\begin{aligned} \alpha _\mathrm {EI}(\varvec{\lambda }| \varvec{\lambda }_{1:n-1}, \bar{f}_{1:n-1})&= \sigma (\varvec{\lambda }| \varvec{\lambda }_{1:n-1}, \bar{f}_{1:n-1})(\gamma (\varvec{\lambda }) \phi (\gamma (\varvec{\lambda })) + \mathcal {N}(\gamma (\varvec{\lambda }))), \\ \gamma (\varvec{\lambda })&= \frac{\mu (\varvec{\lambda }| \varvec{\lambda }_{1:n-1}, \bar{f}_{1:n-1}) - f(\varvec{\lambda }_\text {best})}{\sigma (\varvec{\lambda }| \varvec{\lambda }_{1:n-1}, \bar{f}_{1:n-1})} \end{aligned}$$

where $\mu , \sigma ^2$ are Gaussian process mean and variance predictions, $\varvec{\lambda }_\text {best}$ is a current best solution $f(\varvec{\lambda }_\text {best}) = \max _{i=1,\dots ,n-1} f(\varvec{\lambda }_i)$, $\phi$ is a cumulative density function of the standard normal distribution and $\mathcal {N}$ is a probability density function of the standard normal distribution. Thus, in each iteration we simply select the point that maximizes the above equation

$$\begin{aligned} \alpha _n ( \varvec{\lambda }_{1:n-1}, \bar{f}_{1:n-1} ) = {\arg \max }_{\varvec{\lambda }} \alpha _\mathrm {EI}(\varvec{\lambda }| \varvec{\lambda }_{1:n-1}, \bar{f}_{1:n-1}). \end{aligned}$$

## Endnotes

^a^For example BayesOpt http://rmcantin.bitbucket.org/html/

## Additional files

Additional file 1: Analysis of the time course of accuracy values during execution of the SVM optimization procedure. The file contains the analysis of the changes in accuracy values with different time for SVM optimizations strategies for all targets tested. Additional file 2: AUC values obtained for all target/fingerprint pairs for curves illustrating changes in the accuracy with time and the optimal accuracy values obtained. The file contains the AUC and optimal accuracy values obtained in the experiments. The AUC values were calculated on the basis of curves illustrating changes in the accuracy values in time for different SVM optimization strategies. Additional file 3: Analysis of the number of iterations of the optimization procedure required for reaching the highest accuracy for all tested targets. The file presents the number of iterations after which the optimal accuracy values were reached for all targets tested. Additional file 4: Analysis of the changes of accuracy for different steps for all tested targets. The file presents the set of parameters tested in the subsequent iterations of the optimization procedure with the analysis of classification accuracy values that they were providing.
