# Supplementary material for: Robust optimization of SVM hyperparameters in the classification of bioactive compounds
Source: J Cheminform. 2015 Aug 14;7:38. doi: 10.1186/s13321-015-0088-0 (PMC4534515; doi:10.1186/s13321-015-0088-0)
Supplement: Additional file 1: — Analysis of the time course of accuracy values during execution of the SVM optimization procedure. The file contains the analysis of the changes in accuracy values with different time for SVM optimizations strategies for all targets tested. [file 13321_2015_88_MOESM1_ESM.pdf]

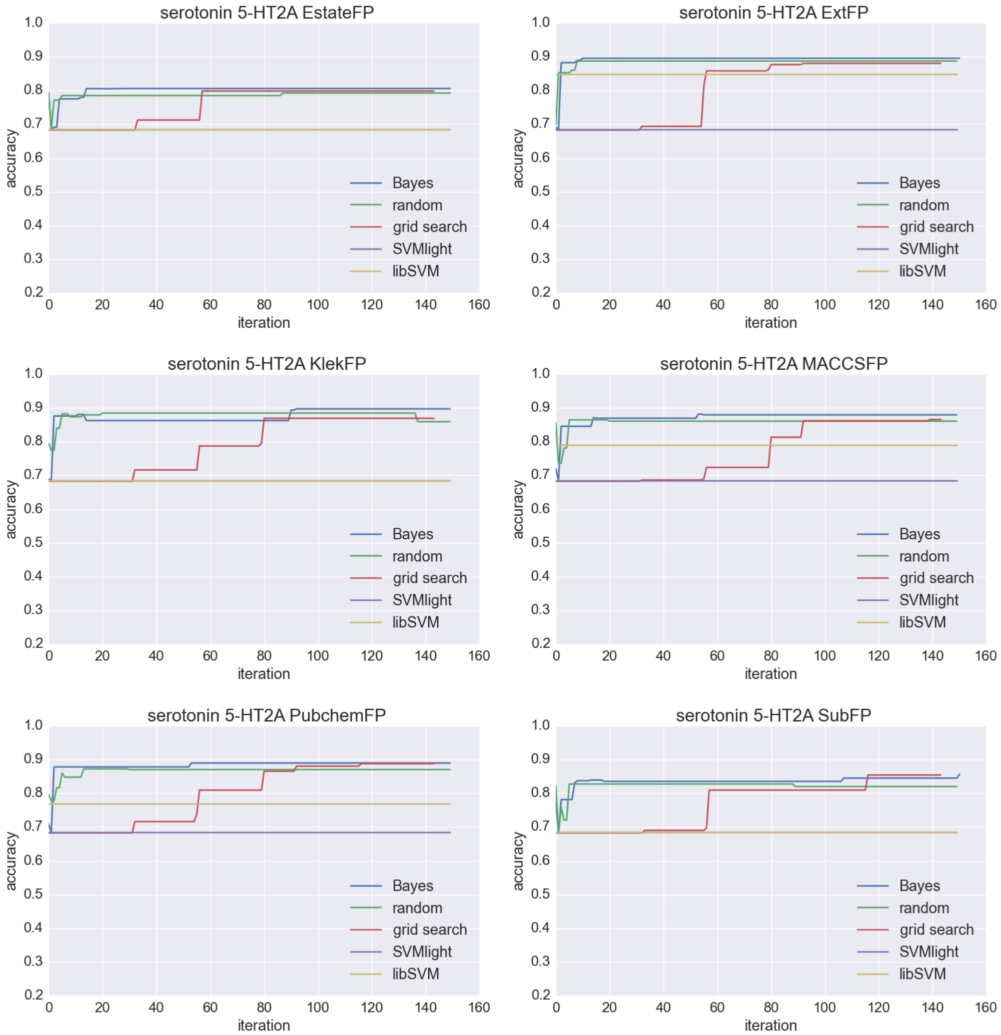

Figure 1: Analysis of the time course of accuracy values during execution of the SVM optimization procedure for serotonin 5-HT2A receptor.

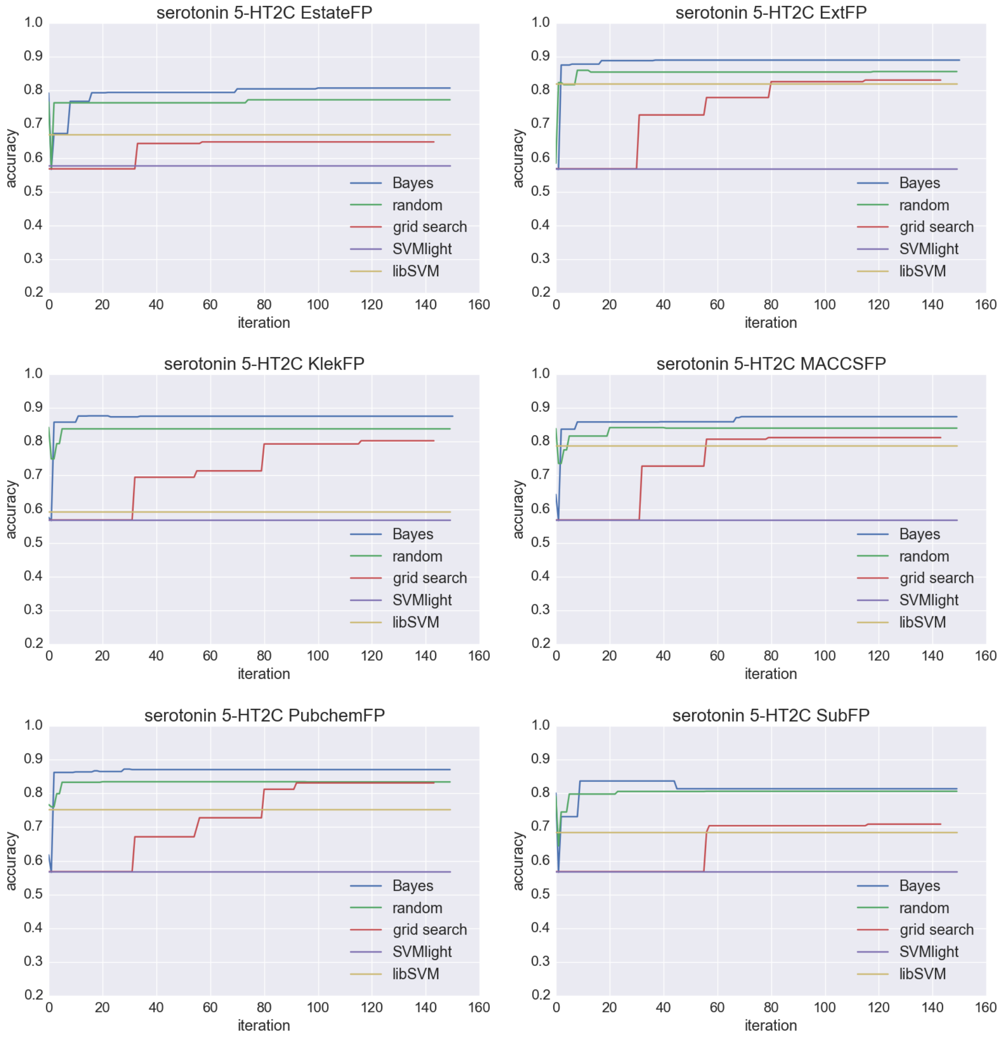

Figure 2: Analysis of the time course of accuracy values during execution of the SVM optimization procedure for serotonin 5-HT2C receptor.

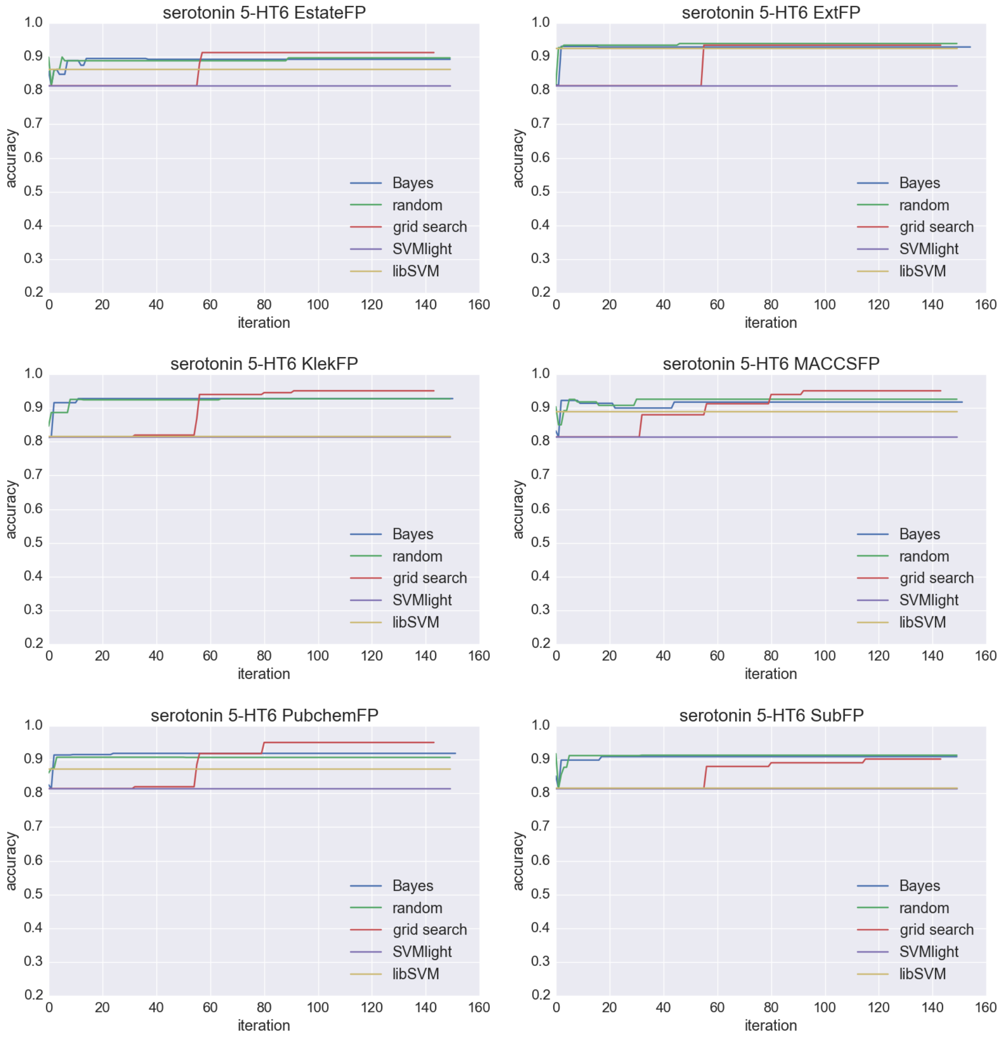

Figure 3: Analysis of the time course of accuracy values during execution of the SVM optimization procedure for serotonin 5-HT6 receptor.

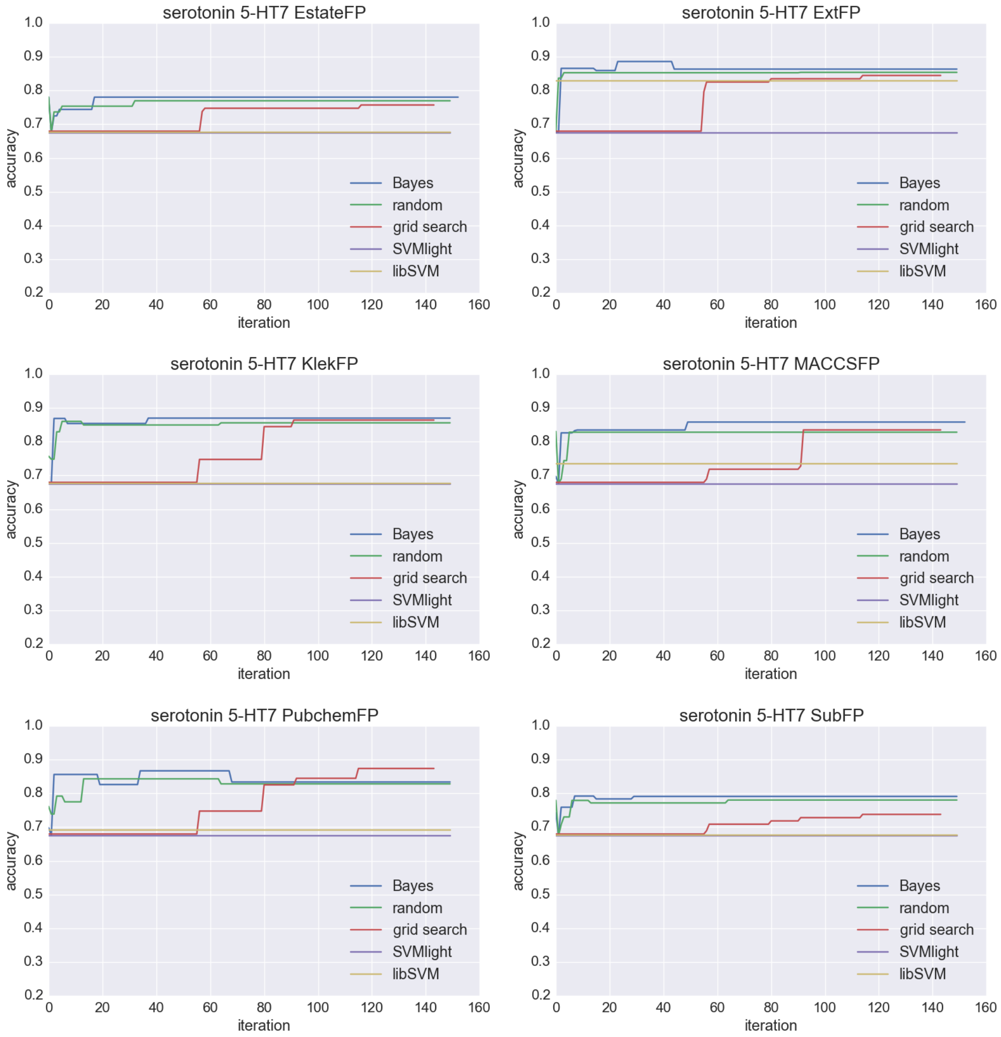

Figure 4: Analysis of the time course of accuracy values during execution of the SVM optimization procedure for serotonin 5-HT7 receptor.

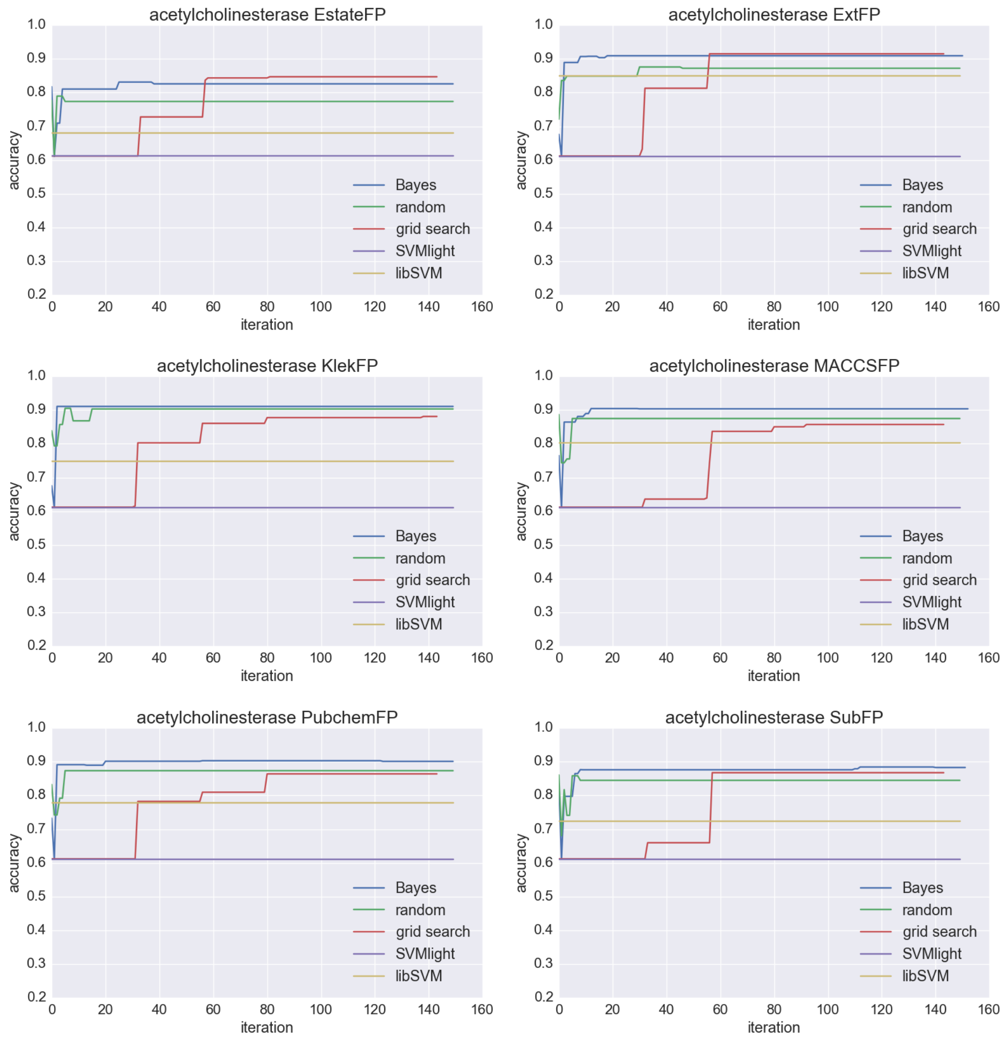

Figure 5: Analysis of the time course of accuracy values during execution of the SVM optimization procedure for acetylcholinesterase.

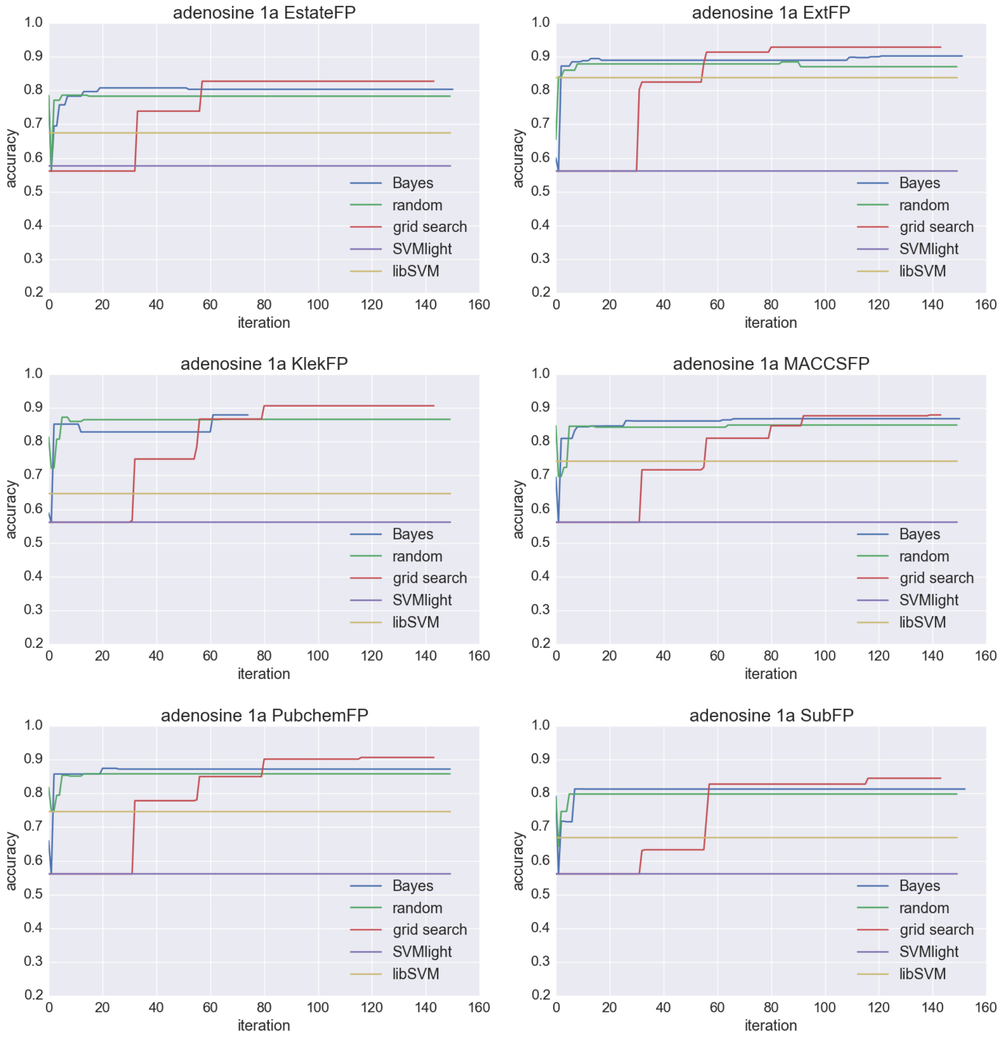

Figure 6: Analysis of the time course of accuracy values during execution of the SVM optimization procedure for adenosine 1a receptor.

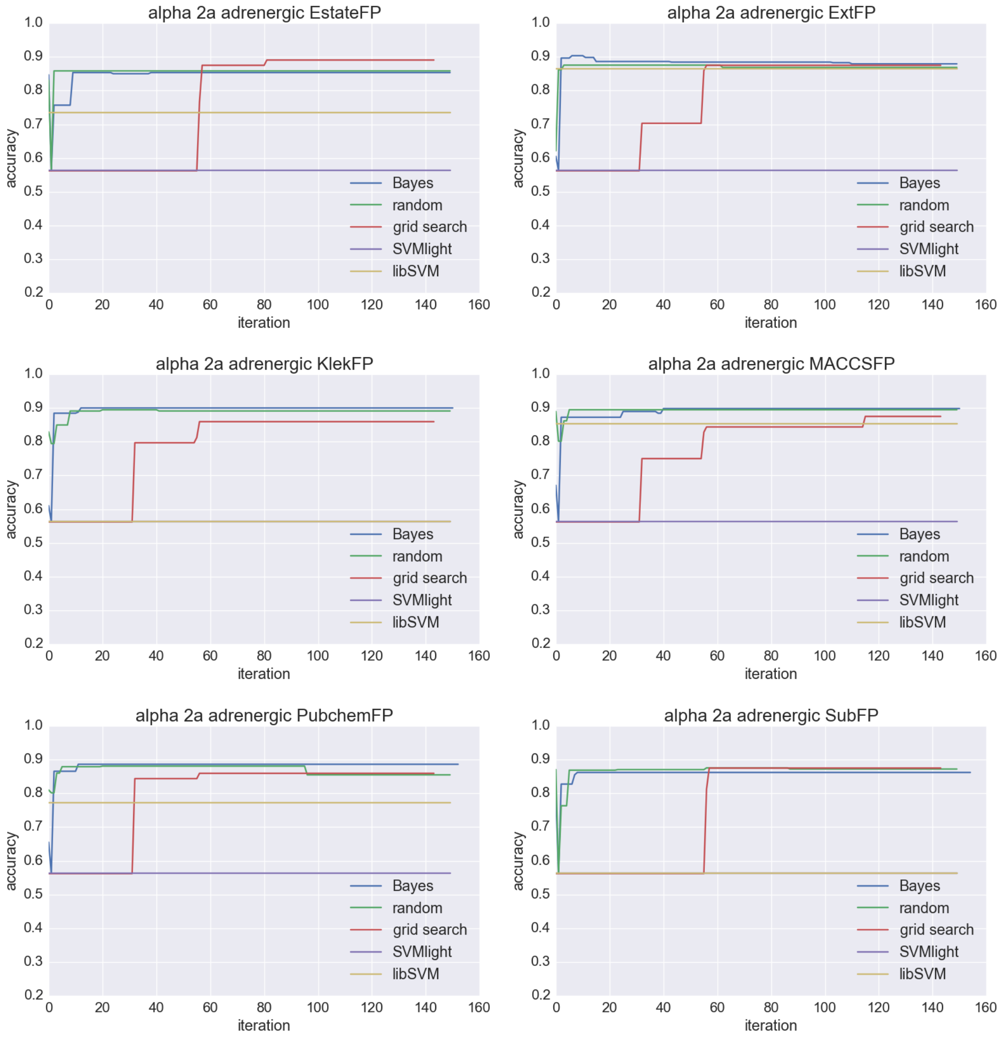

Figure 7: Analysis of the time course of accuracy values during execution of the SVM optimization procedure for alpha 2a AR.

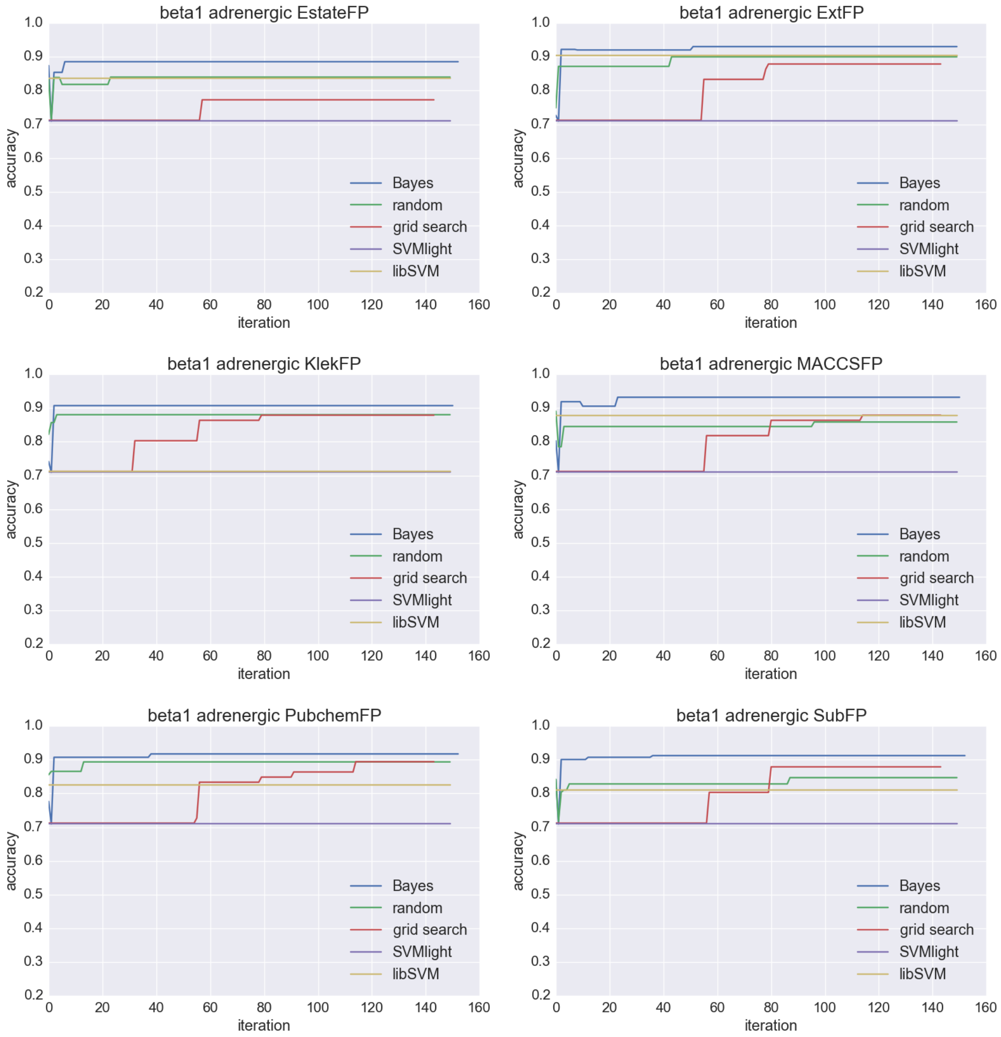

Figure 8: Analysis of the time course of accuracy values during execution of the SVM optimization procedure for beta1AR.

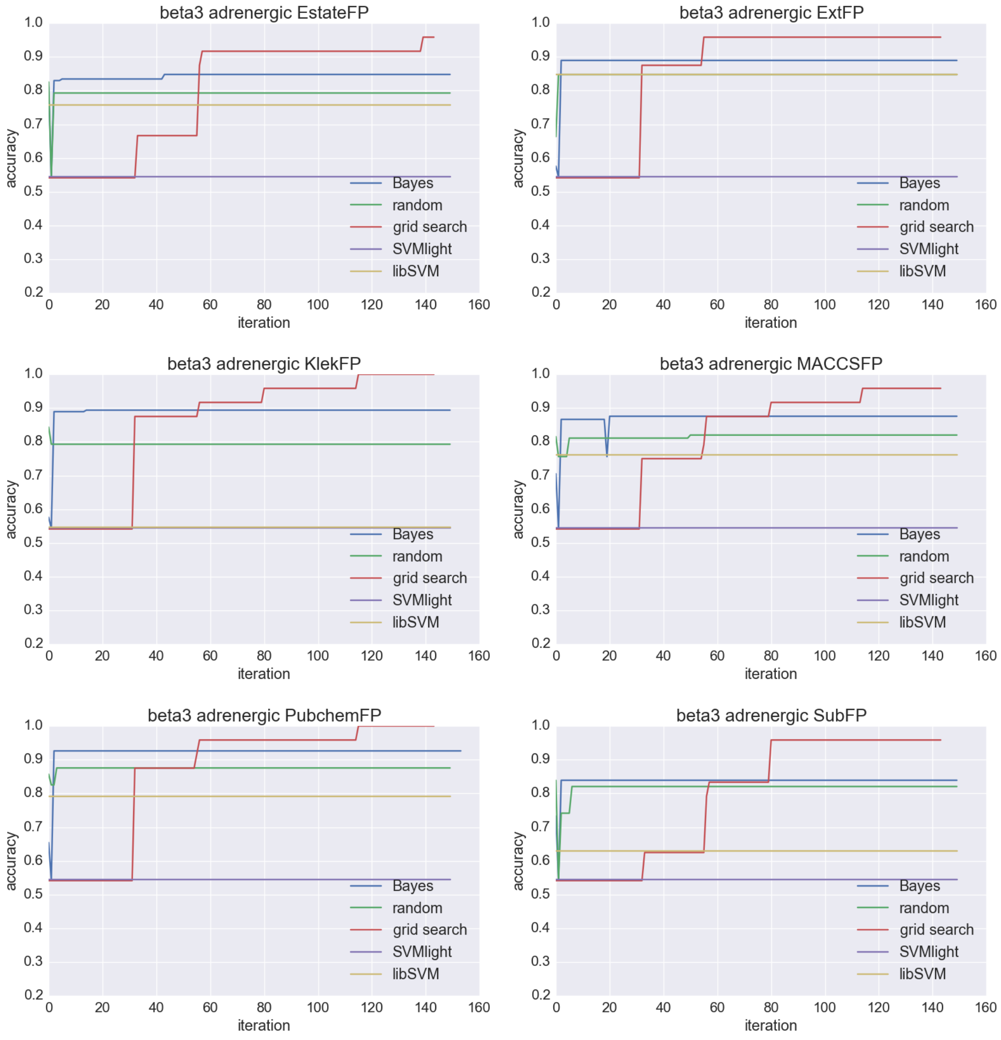

Figure 9: Analysis of the time course of accuracy values during execution of the SVM optimization procedure for beta3AR.

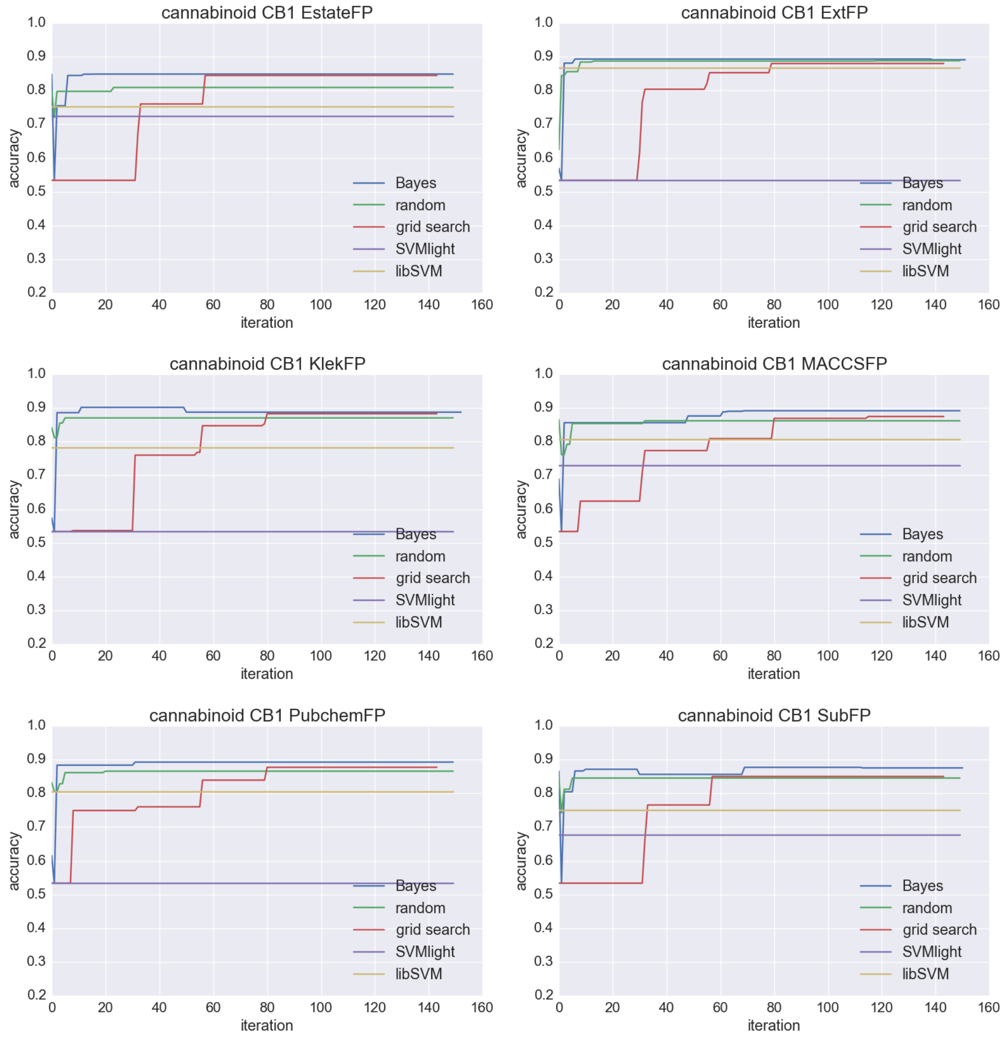

Figure 10: Analysis of the time course of accuracy values during execution of the SVM optimization procedure for cannabinoid CB1 receptor.

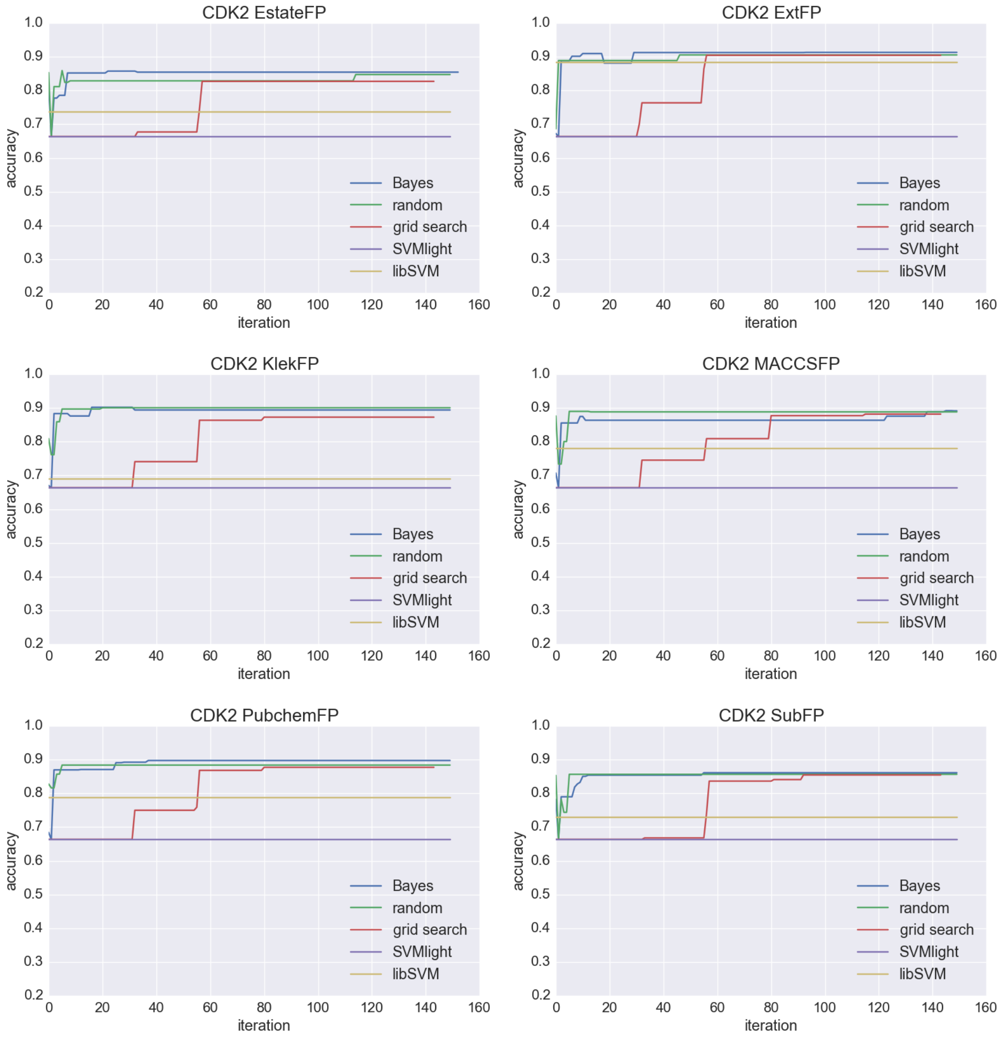

Figure 11: Analysis of the time course of accuracy values during execution of the SVM optimization procedure for CDK2.

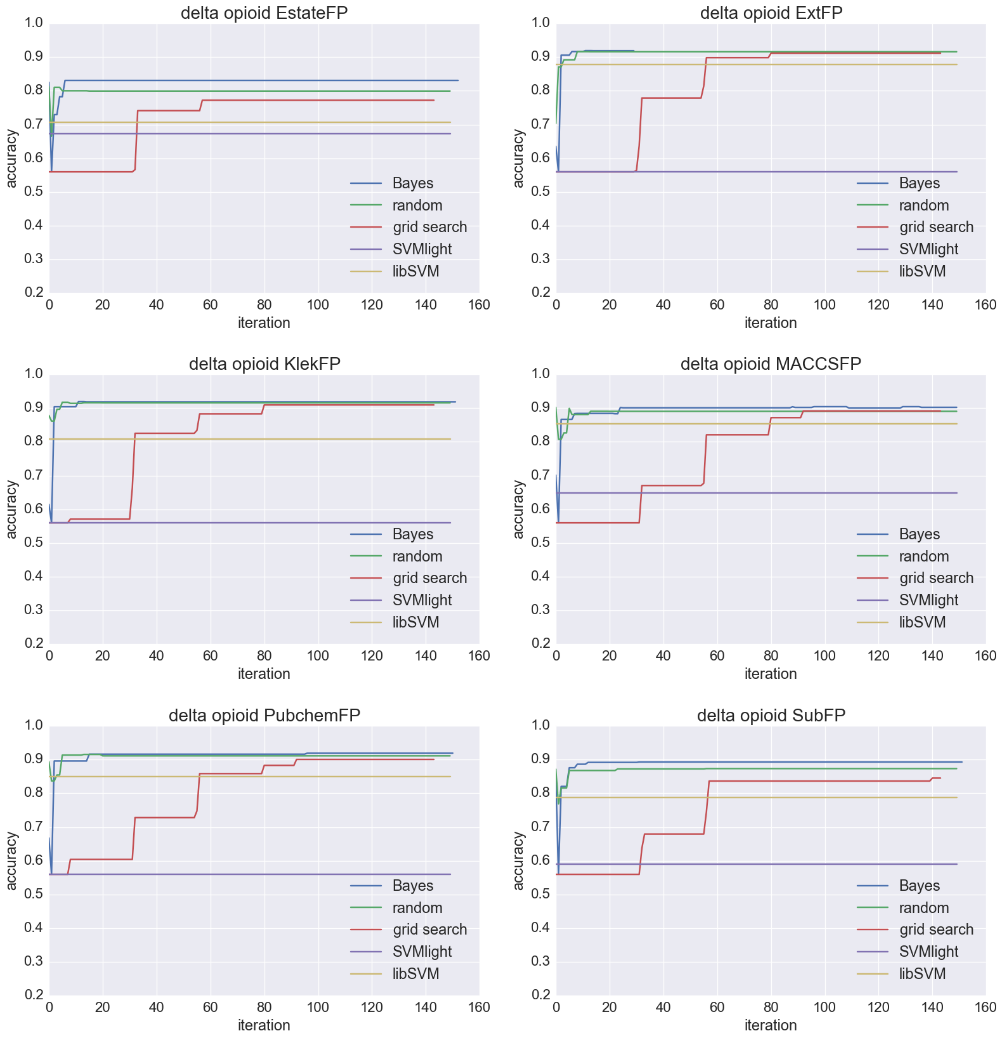

Figure 12: Analysis of the time course of accuracy values during execution of the SVM optimization procedure for delta opioid receptor.

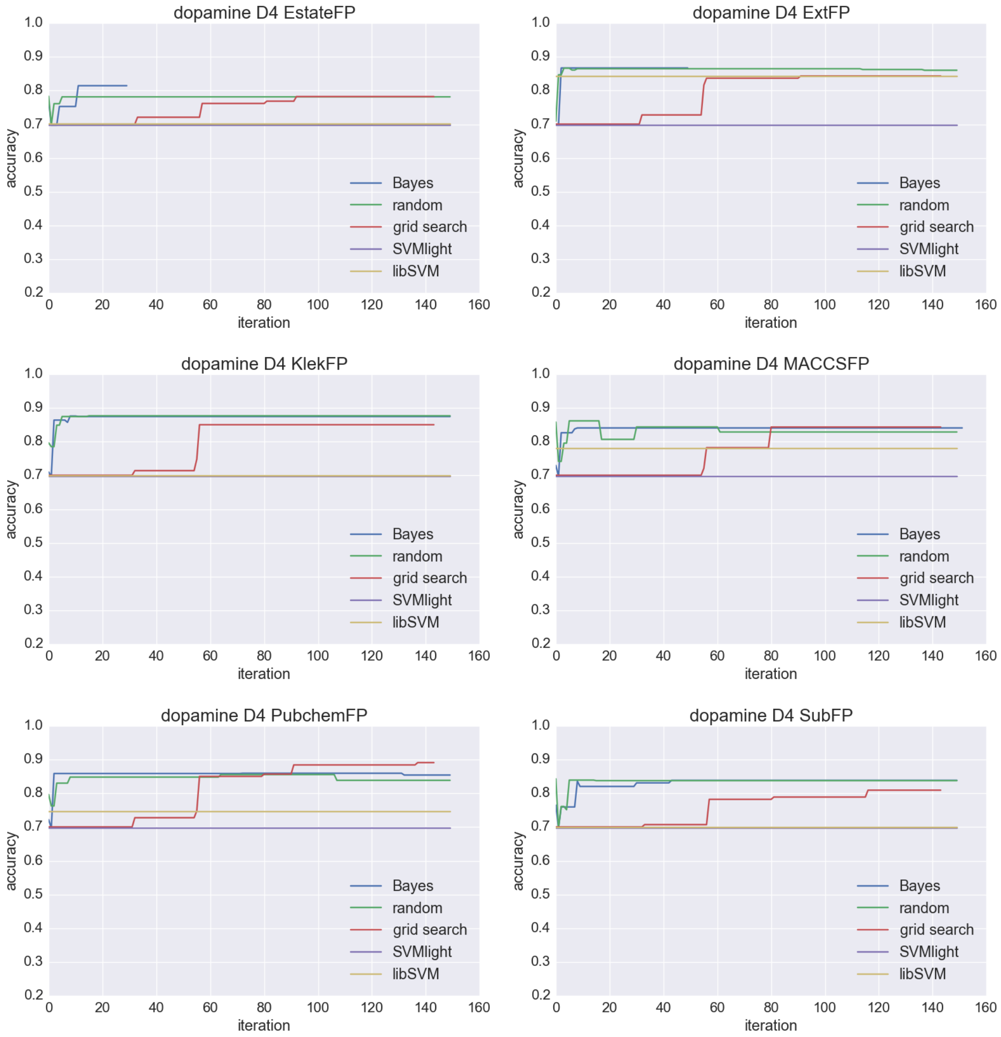

Figure 13: Analysis of the time course of accuracy values during execution of the SVM optimization procedure for dopamine D4 receptor.

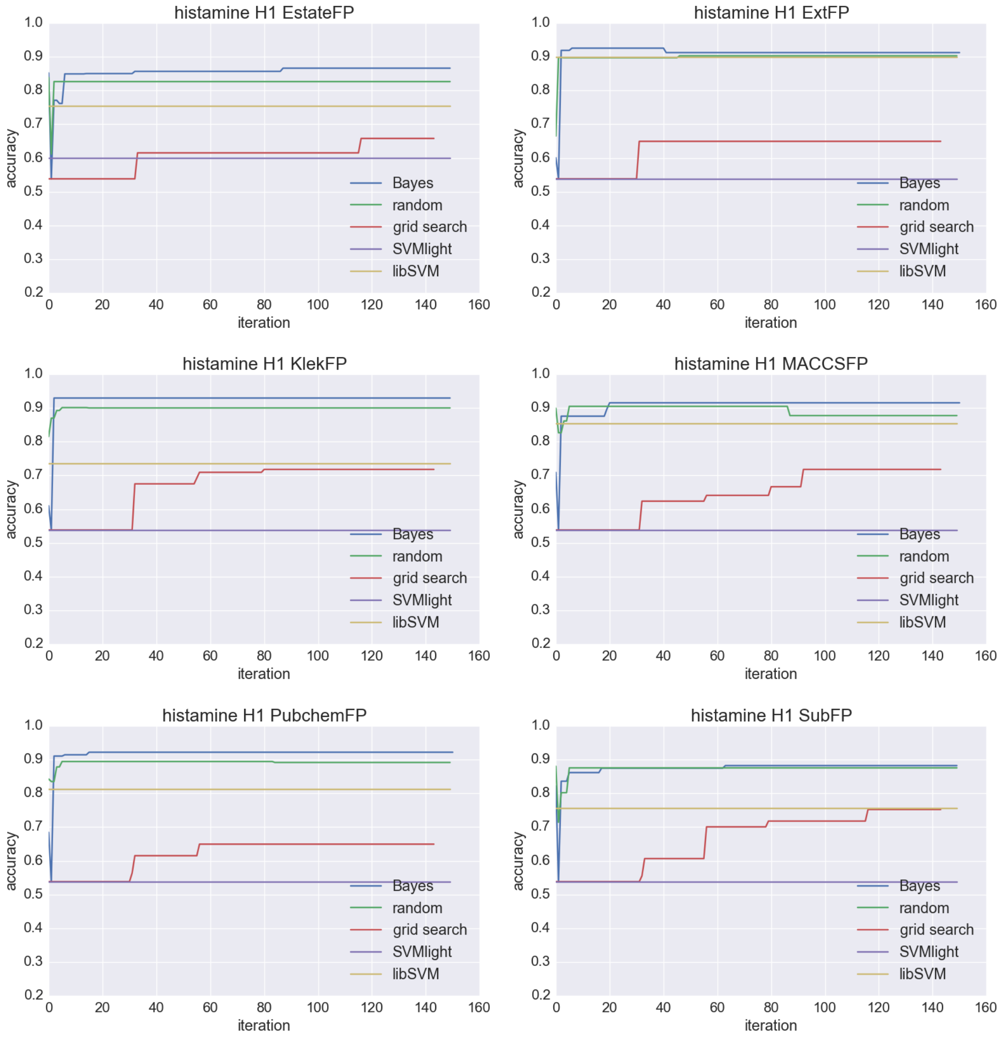

Figure 14: Analysis of the time course of accuracy values during execution of the SVM optimization procedure for H1 receptor.

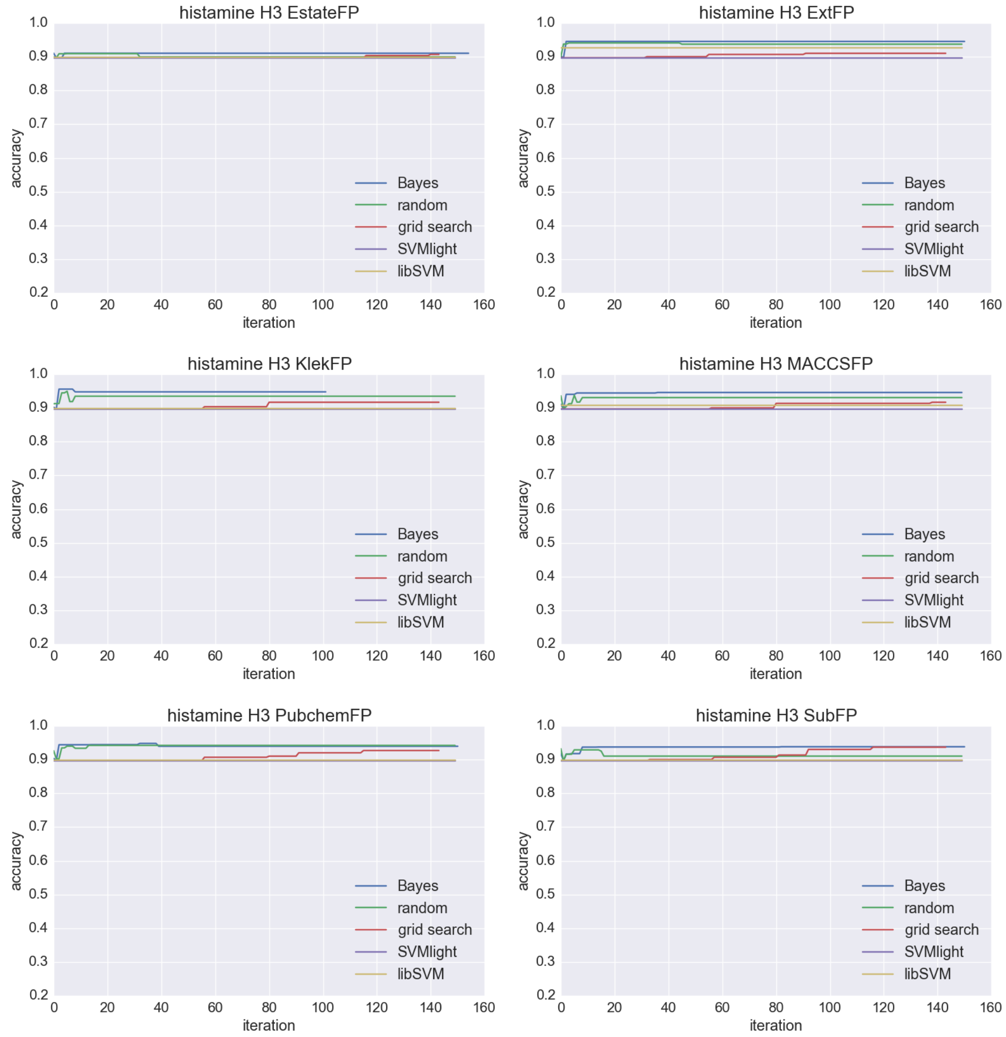

Figure 15: Analysis of the time course of accuracy values during execution of the SVM optimization procedure for histamine H3 receptor.

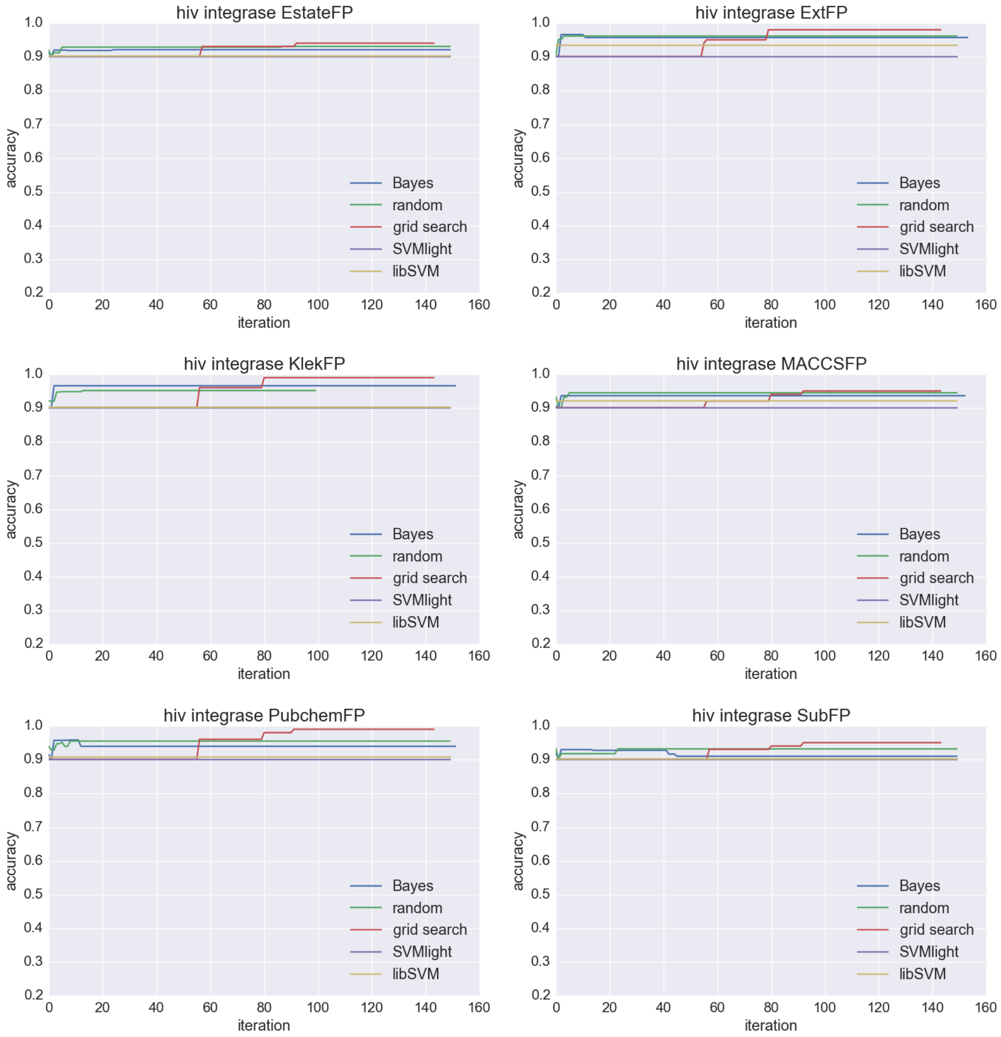

Figure 16: Analysis of the time course of accuracy values during execution of the SVM optimization procedure for HIV integrase.

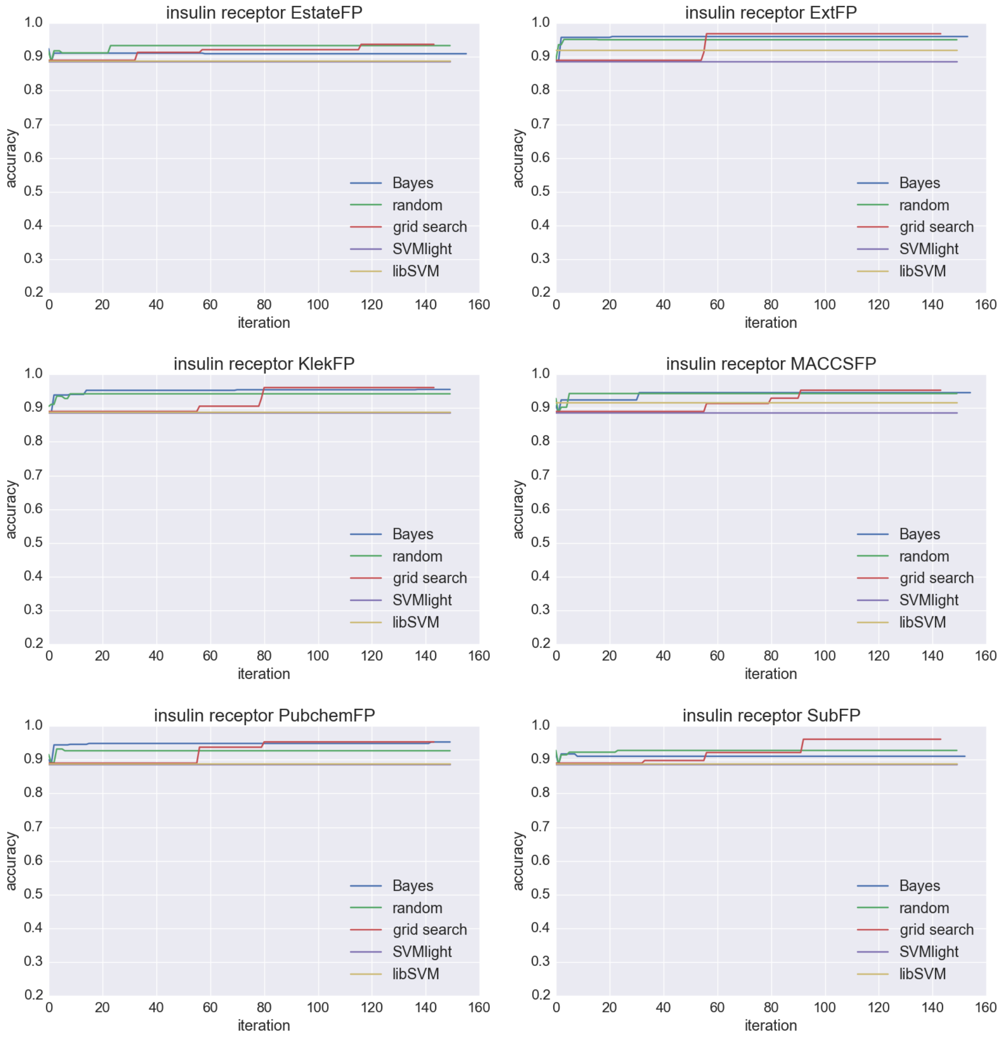

Figure 17: Analysis of the time course of accuracy values during execution of the SVM optimization procedure for insulin receptor.

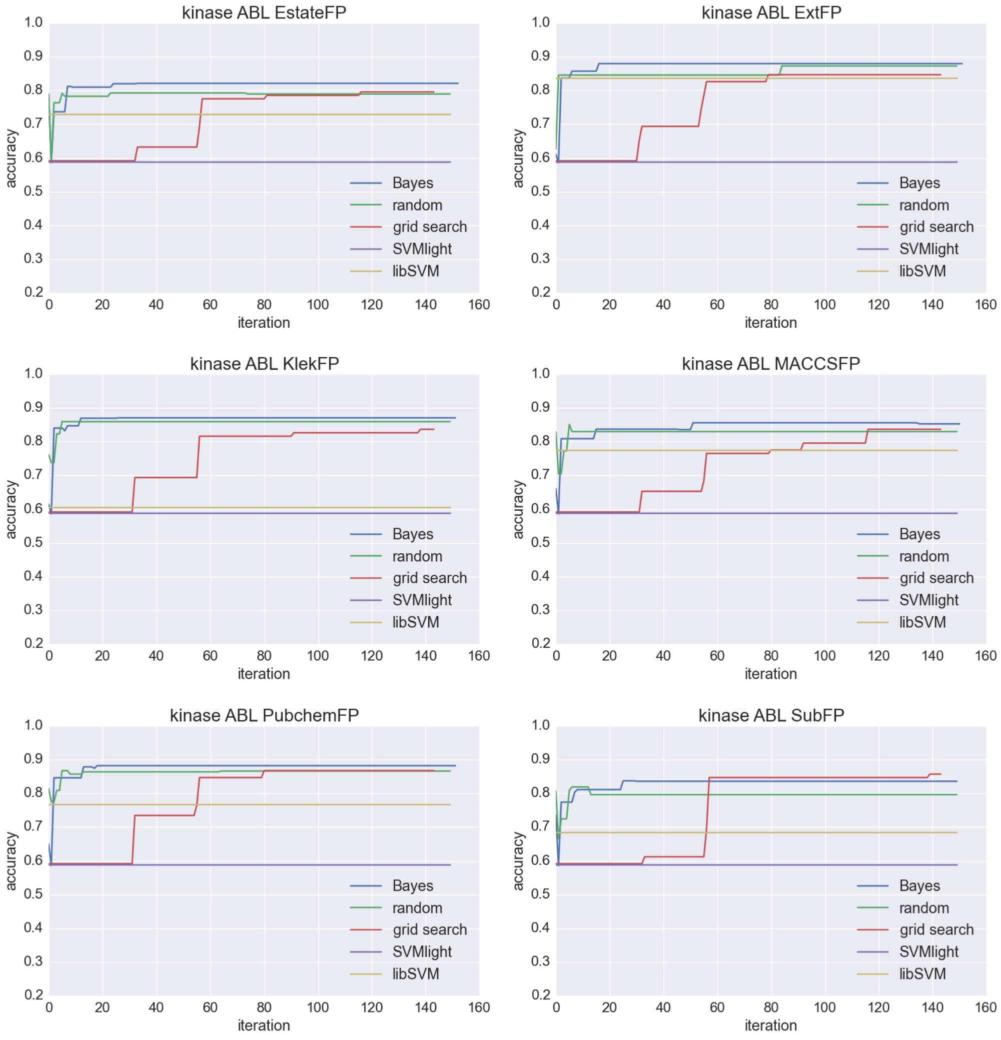

Figure 18: Analysis of the time course of accuracy values during execution of the SVM optimization procedure for kinase ABL.

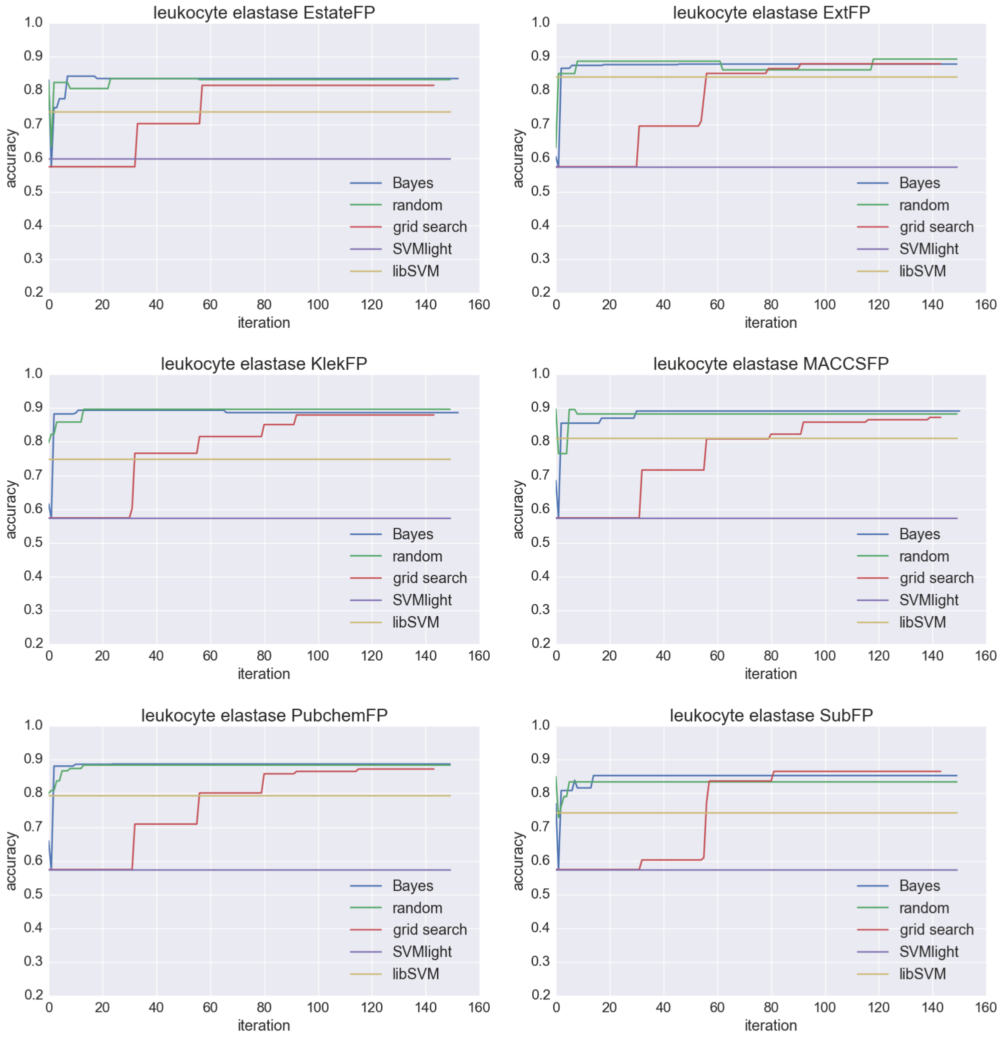

Figure 19: Analysis of the time course of accuracy values during execution of the SVM optimization procedure for leukocyte elastase.

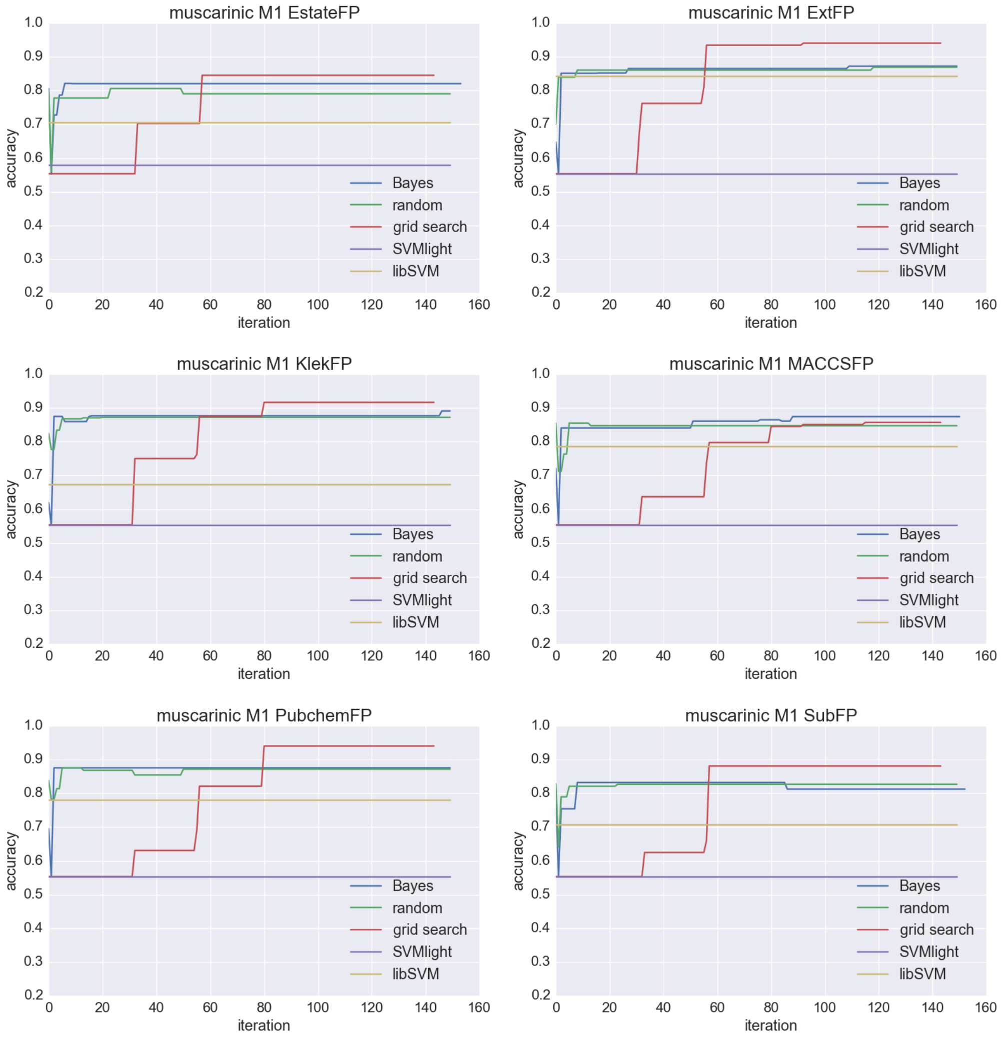

Figure 20: Analysis of the time course of accuracy values during execution of the SVM optimization procedure for muscarinic M1 receptor.

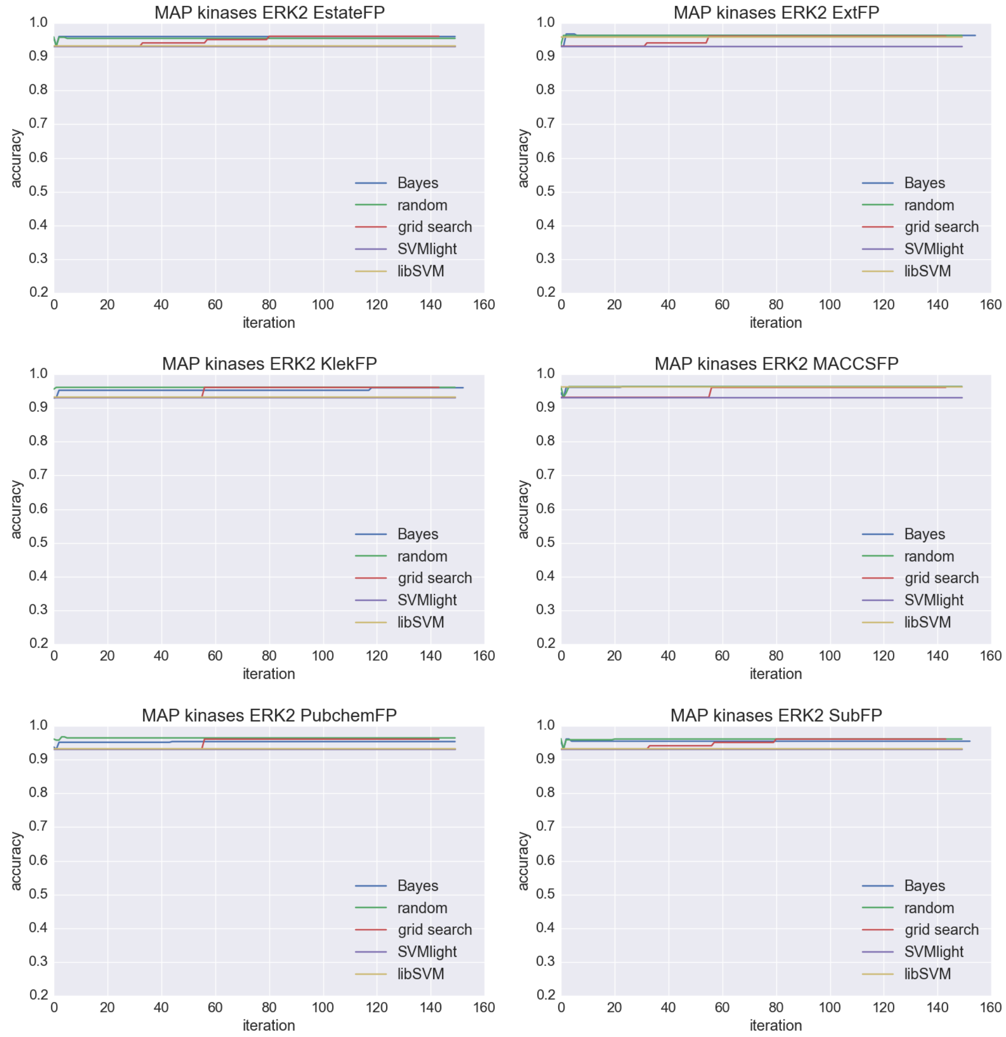

Figure 21: Analysis of the time course of accuracy values during execution of the SVM optimization procedure for MAP kinases ERK2.
