# Supplementary material for: Robust optimization of SVM hyperparameters in the classification of bioactive compounds
Source: J Cheminform. 2015 Aug 14;7:38. doi: 10.1186/s13321-015-0088-0 (PMC4534515; doi:10.1186/s13321-015-0088-0)
Supplement: Additional file 2: — AUC values obtained for all target/fingerprint pairs for curves illustrating changes in the accuracy with time and the optimal accuracy values obtained. The file contains the AUC and optimal accuracy values obtained in the experiments. The AUC values were calculated on the basis of curves illustrating changes in the accuracy values in time for different SVM optimization strategies. [file 13321_2015_88_MOESM2_ESM.pdf]

Table 1: AUC values obtained for all target/fingerprint pairs for curves illustrating changes in the accuracy with time and the optimal accuracy values obtained.

target	optimization method/fingerprint	EstateFP		ExtFP		KlekFP		MACCSFP		PubchemFP		SubFP	
		AUC	final accuracy	AUC	final accuracy	AUC	final accuracy	AUC	final accuracy	AUC	final accuracy	AUC	final accuracy
5-HT$_{2A}$	Bayes	0.801	0.806	0.892	0.896	0.874	0.897	0.872	0.88	0.883	0.89	0.835	0.857
	random	0.787	0.793	0.885	0.887	0.88	0.859	0.858	0.861	0.867	0.87	0.822	0.82
	grid search	0.758	0.799	0.802	0.881	0.789	0.869	0.766	0.866	0.798	0.888	0.769	0.854
	SVMlight	0.683	0.683	0.683	0.683	0.683	0.683	0.683	0.683	0.683	0.683	0.683	0.683
	libSVM	0.684	0.684	0.847	0.847	0.684	0.684	0.789	0.789	0.769	0.769	0.684	0.684
5-HT$_{2C}$	Bayes	0.792	0.807	0.884	0.89	0.87	0.876	0.862	0.874	0.865	0.87	0.814	0.814
	random	0.767	0.773	0.852	0.856	0.836	0.838	0.836	0.84	0.832	0.834	0.803	0.806
	grid search	0.629	0.648	0.747	0.831	0.716	0.803	0.743	0.812	0.727	0.831	0.652	0.709
	SVMlight	0.577	0.577	0.566	0.566	0.566	0.566	0.566	0.566	0.566	0.566	0.566	0.566
	libSVM	0.668	0.668	0.82	0.82	0.591	0.591	0.788	0.788	0.752	0.752	0.684	0.684
5-HT$_6$	Bayes	0.89	0.893	0.928	0.929	0.925	0.928	0.913	0.917	0.917	0.919	0.907	0.909
	random	0.891	0.897	0.937	0.939	0.923	0.927	0.922	0.926	0.906	0.907	0.911	0.913
	grid search	0.874	0.913	0.889	0.934	0.897	0.951	0.901	0.951	0.894	0.951	0.861	0.902
	SVMlight	0.814	0.814	0.814	0.814	0.814	0.814	0.814	0.814	0.814	0.814	0.814	0.814
	libSVM	0.863	0.863	0.924	0.924	0.815	0.815	0.888	0.888	0.872	0.872	0.815	0.815
5-HT$_7$	Bayes	0.776	0.78	0.864	0.864	0.864	0.87	0.848	0.858	0.841	0.834	0.788	0.791
	random	0.765	0.77	0.852	0.854	0.851	0.856	0.825	0.828	0.828	0.828	0.775	0.78
	grid search	0.722	0.757	0.776	0.845	0.771	0.864	0.745	0.835	0.769	0.874	0.707	0.738
	SVMlight	0.675	0.675	0.675	0.675	0.675	0.675	0.675	0.675	0.675	0.675	0.675	0.675
	libSVM	0.676	0.676	0.828	0.828	0.676	0.676	0.734	0.734	0.691	0.691	0.677	0.677
AchE	Bayes	0.821	0.826	0.904	0.909	0.907	0.911	0.899	0.904	0.897	0.901	0.873	0.883
	random	0.773	0.774	0.867	0.872	0.899	0.903	0.871	0.875	0.87	0.873	0.842	0.845
	grid search	0.773	0.847	0.831	0.915	0.803	0.881	0.761	0.857	0.785	0.864	0.774	0.867
	SVMlight	0.613	0.613	0.611	0.611	0.611	0.611	0.611	0.611	0.611	0.611	0.611	0.611
	libSVM	0.68	0.68	0.849	0.849	0.747	0.747	0.803	0.803	0.778	0.778	0.724	0.724
A$_1$	Bayes	0.799	0.804	0.888	0.902	0.835	0.879	0.858	0.868	0.867	0.872	0.808	0.813
	random	0.782	0.783	0.873	0.871	0.859	0.866	0.843	0.849	0.855	0.858	0.796	0.798
	grid search	0.752	0.828	0.829	0.929	0.699	0.906	0.767	0.879	0.798	0.906	0.739	0.845
	SVMlight	0.577	0.577	0.561	0.561	0.561	0.561	0.561	0.561	0.561	0.561	0.561	0.561
	libSVM	0.675	0.675	0.839	0.839	0.647	0.647	0.742	0.742	0.746	0.746	0.669	0.669
alpha2AR	Bayes	0.846	0.853	0.881	0.879	0.895	0.9	0.889	0.898	0.881	0.886	0.858	0.862
	random	0.856	0.858	0.87	0.869	0.888	0.891	0.893	0.895	0.87	0.855	0.868	0.872

	grid search	0.76	0.891	0.778	0.875	0.783	0.859	0.772	0.875	0.791	0.859	0.753	0.875
	SVMlight	0.563	0.563	0.563	0.563	0.563	0.563	0.563	0.563	0.563	0.563	0.563	0.563
	libSVM	0.734	0.734	0.865	0.865	0.564	0.564	0.852	0.852	0.773	0.773	0.564	0.564
beta1AR	Bayes	0.883	0.886	0.924	0.93	0.905	0.907	0.926	0.932	0.912	0.917	0.909	0.912
	random	0.836	0.84	0.891	0.9	0.879	0.88	0.849	0.859	0.891	0.893	0.835	0.847
	grid search	0.749	0.773	0.808	0.879	0.827	0.879	0.8	0.879	0.805	0.894	0.801	0.879
	SVMlight	0.710	0.710	0.710	0.710	0.710	0.710	0.710	0.710	0.710	0.710	0.710	0.710
	libSVM	0.837	0.837	0.904	0.904	0.711	0.711	0.879	0.879	0.825	0.825	0.81	0.81
beta3AR	Bayes	0.842	0.848	0.885	0.889	0.889	0.894	0.87	0.876	0.922	0.926	0.836	0.839
	random	0.791	0.793	0.846	0.848	0.793	0.793	0.815	0.82	0.875	0.876	0.817	0.821
	grid search	0.958	0.792	0.852	0.958	0.853	1	0.808	0.958	0.861	1	0.788	0.958
	SVMlight	0.545	0.545	0.545	0.545	0.545	0.545	0.545	0.545	0.545	0.545	0.545	0.545
	libSVM	0.758	0.758	0.848	0.848	0.546	0.546	0.762	0.762	0.791	0.791	0.629	0.629
CB1	Bayes	0.844	0.849	0.888	0.891	0.887	0.888	0.875	0.892	0.886	0.893	0.866	0.875
	random	0.807	0.809	0.884	0.888	0.869	0.871	0.858	0.862	0.864	0.866	0.844	0.845
	grid search	0.76	0.845	0.788	0.88	0.781	0.883	0.786	0.875	0.811	0.877	0.765	0.85
	SVMlight	0.724	0.724	0.534	0.534	0.534	0.534	0.729	0.729	0.534	0.534	0.676	0.676
	libSVM	0.751	0.751	0.867	0.867	0.782	0.782	0.805	0.805	0.804	0.804	0.75	0.75
CDK2	Bayes	0.85	0.855	0.906	0.913	0.89	0.894	0.863	0.891	0.89	0.897	0.854	0.861
	random	0.831	0.847	0.899	0.906	0.897	0.901	0.885	0.888	0.882	0.884	0.853	0.857
	grid search	0.765	0.827	0.828	0.905	0.803	0.873	0.797	0.882	0.807	0.877	0.776	0.855
	SVMlight	0.664	0.664	0.664	0.664	0.664	0.664	0.664	0.664	0.664	0.664	0.664	0.664
	libSVM	0.737	0.737	0.883	0.883	0.689	0.689	0.78	0.78	0.787	0.787	0.729	0.729
DOR	Bayes	0.827	0.831	0.895	0.919	0.913	0.918	0.894	0.902	0.911	0.919	0.888	0.893
	random	0.799	0.799	0.902	0.916	0.914	0.916	0.888	0.89	0.909	0.911	0.87	0.873
	grid search	0.718	0.772	0.56	0.912	0.816	0.909	0.768	0.892	0.795	0.9	0.748	0.845
	SVMlight	0.673	0.673	0.56	0.56	0.56	0.56	0.649	0.649	0.56	0.56	0.591	0.591
	libSVM	0.707	0.707	0.878	0.878	0.808	0.808	0.852	0.852	0.849	0.849	0.788	0.788
D_4	Bayes	0.787	0.815	0.86	0.867	0.872	0.875	0.839	0.841	0.857	0.854	0.831	0.839
	random	0.777	0.782	0.861	0.86	0.874	0.877	0.831	0.829	0.846	0.839	0.835	0.838
	grid search	0.701	0.782	0.71	0.844	0.795	0.85	0.778	0.844	0.81	0.891	0.758	0.81
	SVMlight	0.698	0.698	0.698	0.698	0.698	0.698	0.698	0.698	0.698	0.698	0.698	0.698
	libSVM	0.701	0.701	0.843	0.843	0.699	0.699	0.78	0.78	0.746	0.746	0.699	0.699
H_1	Bayes	0.855	0.866	0.911	0.912	0.924	0.929	0.906	0.915	0.917	0.922	0.874	0.882
	random	0.825	0.827	0.899	0.903	0.899	0.9	0.892	0.877	0.892	0.892	0.873	0.876
	grid search	0.606	0.658	0.626	0.65	0.67	0.718	0.645	0.718	0.619	0.65	0.663	0.752
	SVMlight	0.6	0.6	0.538	0.538	0.538	0.538	0.538	0.538	0.538	0.538	0.538	0.538

	libSVM	0.754	0.754	0.897	0.897	0.734	0.734	0.853	0.853	0.812	0.812	0.756	0.756
H_3	Bayes	0.91	0.91	0.945	0.945	0.947	0.948	0.945	0.946	0.94	0.94	0.936	0.938
	random	0.902	0.9	0.938	0.937	0.934	0.935	0.93	0.931	0.941	0.942	0.912	0.91
	grid search	0.898	0.907	0.905	0.91	0.903	0.917	0.905	0.917	0.91	0.927	0.914	0.937
	SVMlight	0.897	0.897	0.897	0.897	0.897	0.897	0.897	0.897	0.897	0.897	0.897	0.897
	libSVM	0.898	0.898	0.927	0.927	0.898	0.898	0.908	0.908	0.898	0.898	0.898	0.898
HIVi	Bayes	0.921	0.921	0.957	0.957	0.965	0.966	0.936	0.936	0.941	0.94	0.915	0.91
	random	0.929	0.931	0.961	0.962	0.95	0.952	0.944	0.945	0.954	0.955	0.93	0.932
	grid search	0.923	0.941	0.945	0.98	0.933	0.99	0.925	0.95	0.95	0.99	0.927	0.95
	SVMlight	0.901	0.901	0.901	0.901	0.901	0.901	0.901	0.901	0.901	0.901	0.901	0.901
	libSVM	0.902	0.902	0.934	0.934	0.902	0.902	0.921	0.921	0.907	0.907	0.902	0.902
IR	Bayes	0.91	0.909	0.959	0.96	0.951	0.955	0.941	0.946	0.947	0.952	0.91	0.91
	random	0.93	0.933	0.95	0.951	0.941	0.942	0.942	0.943	0.926	0.927	0.926	0.927
	grid search	0.916	0.937	0.938	0.969	0.924	0.961	0.92	0.953	0.926	0.953	0.924	0.961
	SVMlight	0.886	0.886	0.886	0.886	0.886	0.886	0.886	0.886	0.886	0.886	0.886	0.886
	libSVM	0.887	0.887	0.919	0.919	0.887	0.887	0.915	0.915	0.887	0.887	0.887	0.887
ABL	Bayes	0.815	0.821	0.873	0.88	0.865	0.871	0.844	0.853	0.875	0.882	0.828	0.836
	random	0.788	0.79	0.856	0.873	0.857	0.859	0.828	0.83	0.863	0.866	0.795	0.796
	grid search	0.716	0.796	0.763	0.847	0.75	0.837	0.728	0.837	0.781	0.867	0.75	0.857
	SVMlight	0.587	0.587	0.587	0.587	0.587	0.587	0.587	0.587	0.587	0.587	0.587	0.587
	libSVM	0.729	0.729	0.836	0.836	0.606	0.606	0.774	0.774	0.766	0.766	0.684	0.684
HLE	Bayes	0.832	0.836	0.874	0.879	0.885	0.886	0.882	0.891	0.883	0.887	0.847	0.853
	random	0.829	0.833	0.875	0.893	0.892	0.897	0.88	0.882	0.881	0.884	0.833	0.834
	grid search	0.741	0.816	0.777	0.879	0.78	0.879	0.762	0.872	0.765	0.872	0.752	0.865
	SVMlight	0.597	0.597	0.574	0.574	0.574	0.574	0.574	0.574	0.574	0.574	0.574	0.574
	libSVM	0.738	0.738	0.84	0.84	0.747	0.747	0.81	0.81	0.794	0.794	0.743	0.743
M_1	Bayes	0.817	0.821	0.861	0.872	0.872	0.891	0.856	0.874	0.872	0.876	0.819	0.813
	random	0.79	0.79	0.86	0.869	0.87	0.872	0.845	0.847	0.867	0.872	0.824	0.827
	grid search	0.755	0.845	0.824	0.94	0.801	0.917	0.741	0.857	0.783	0.94	0.764	0.881
	SVMlight	0.578	0.578	0.553	0.553	0.553	0.553	0.553	0.553	0.553	0.553	0.553	0.553
	libSVM	0.705	0.705	0.841	0.841	0.672	0.672	0.785	0.785	0.781	0.781	0.706	0.706
ERK2	Bayes	0.96	0.96	0.963	0.963	0.953	0.96	0.963	0.963	0.953	0.954	0.955	0.955
	random	0.955	0.955	0.963	0.963	0.961	0.961	0.963	0.963	0.964	0.964	0.961	0.961
	grid search	0.949	0.961	0.951	0.961	0.949	0.961	0.949	0.961	0.949	0.961	0.949	0.961
	SVMlight	0.931	0.931	0.931	0.931	0.931	0.931	0.931	0.931	0.931	0.931	0.931	0.931
	libSVM	0.932	0.932	0.958	0.958	0.932	0.932	0.963	0.963	0.932	0.932	0.932	0.932
