# Supplementary material for: Robust optimization of SVM hyperparameters in the classification of bioactive compounds
Source: J Cheminform. 2015 Aug 14;7:38. doi: 10.1186/s13321-015-0088-0 (PMC4534515; doi:10.1186/s13321-015-0088-0)
Supplement: Additional file 3: — Analysis of the number of iterations of the optimization procedure required for reaching the highest accuracy for all tested targets. The file presents the number of iterations after which the optimal accuracy values were reached for all targets tested. [file 13321_2015_88_MOESM3_ESM.pdf]

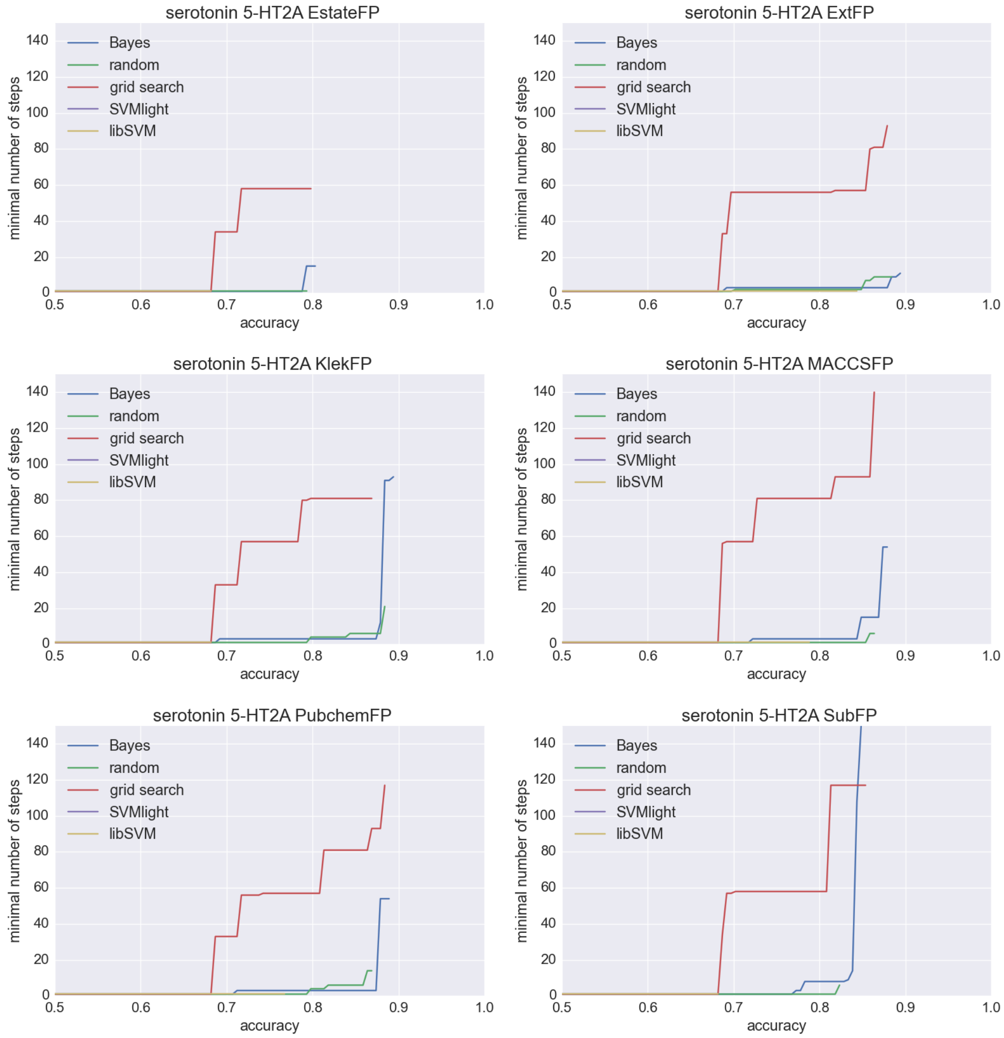

Figure 1: Analysis of the number of iterations of the optimization procedure required for reaching the highest accuracy for 5-HT2A receptor.

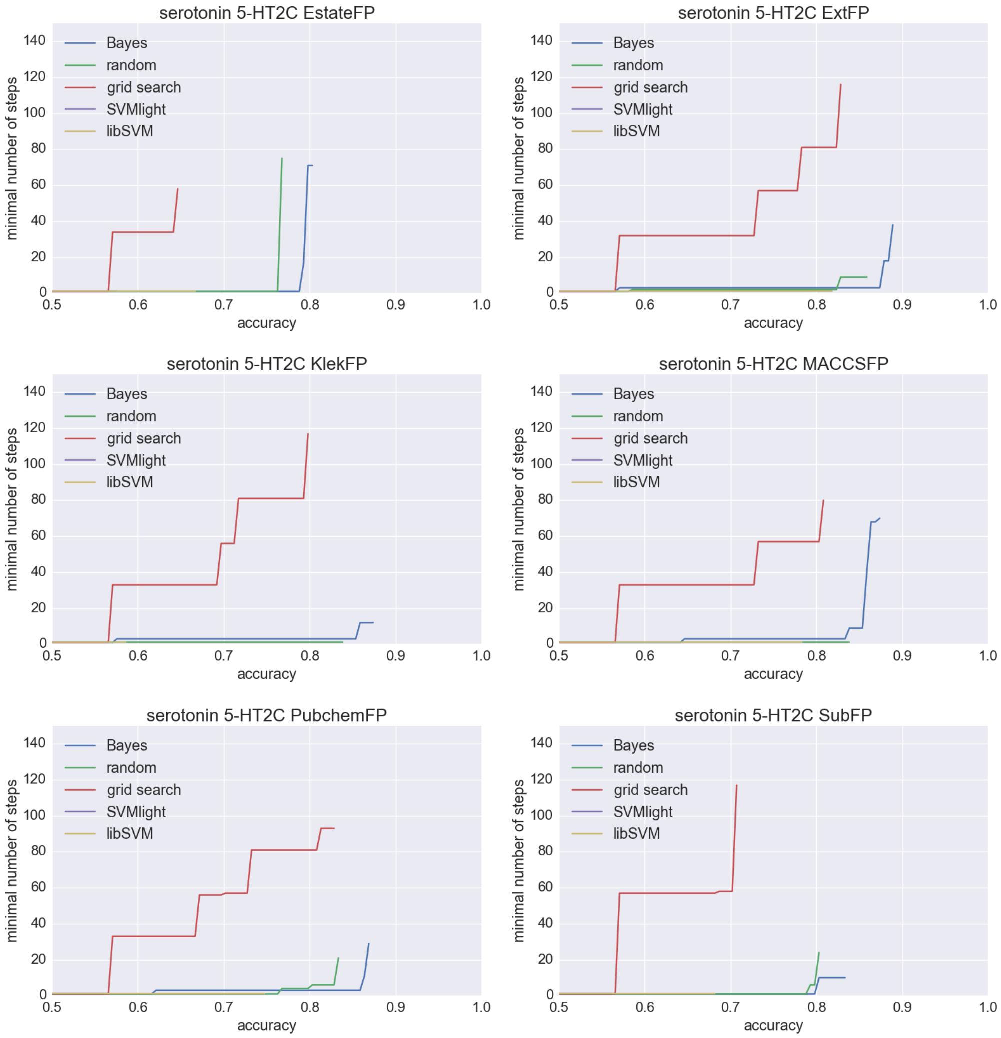

Figure 2: Analysis of the number of iterations of the optimization procedure required for reaching the highest accuracy for 5-HT2C receptor.

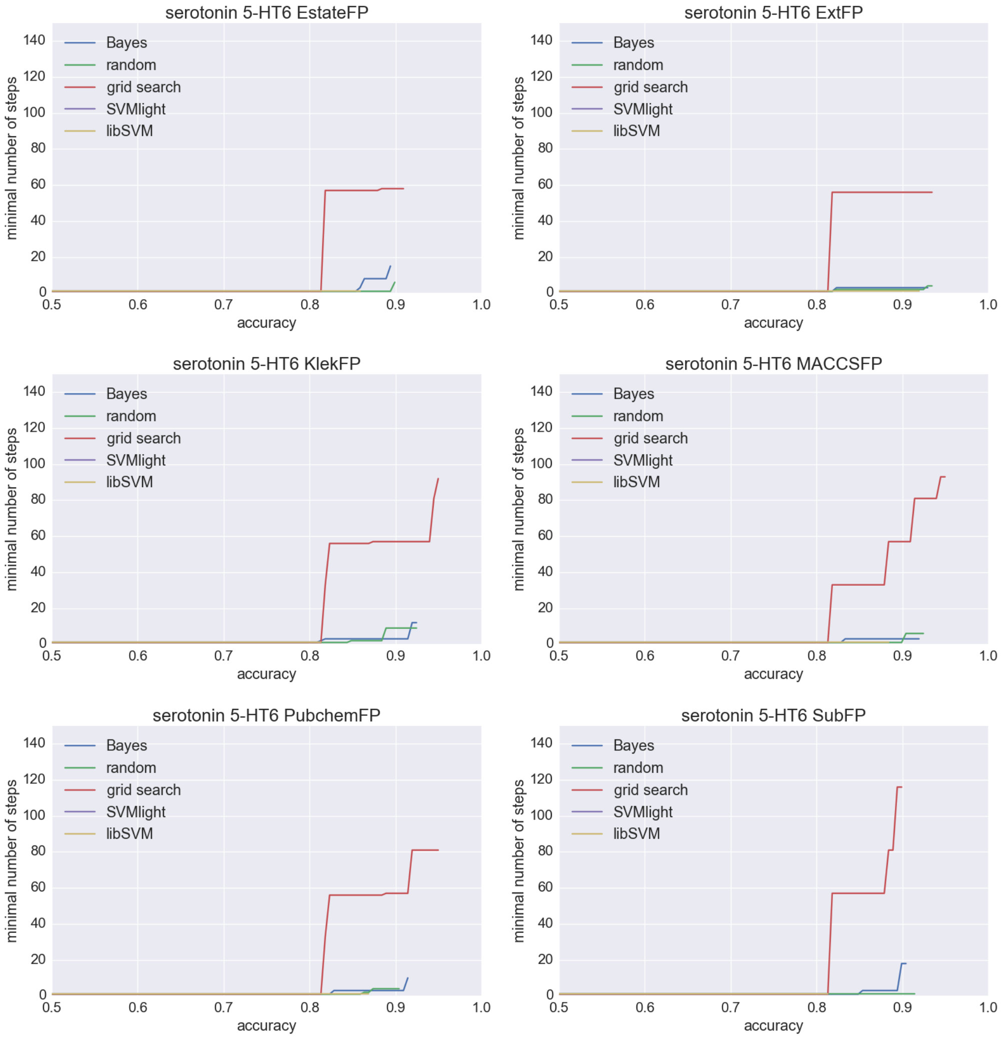

Figure 3: Analysis of the number of iterations of the optimization procedure required for reaching the highest accuracy for 5-HT6 receptor.

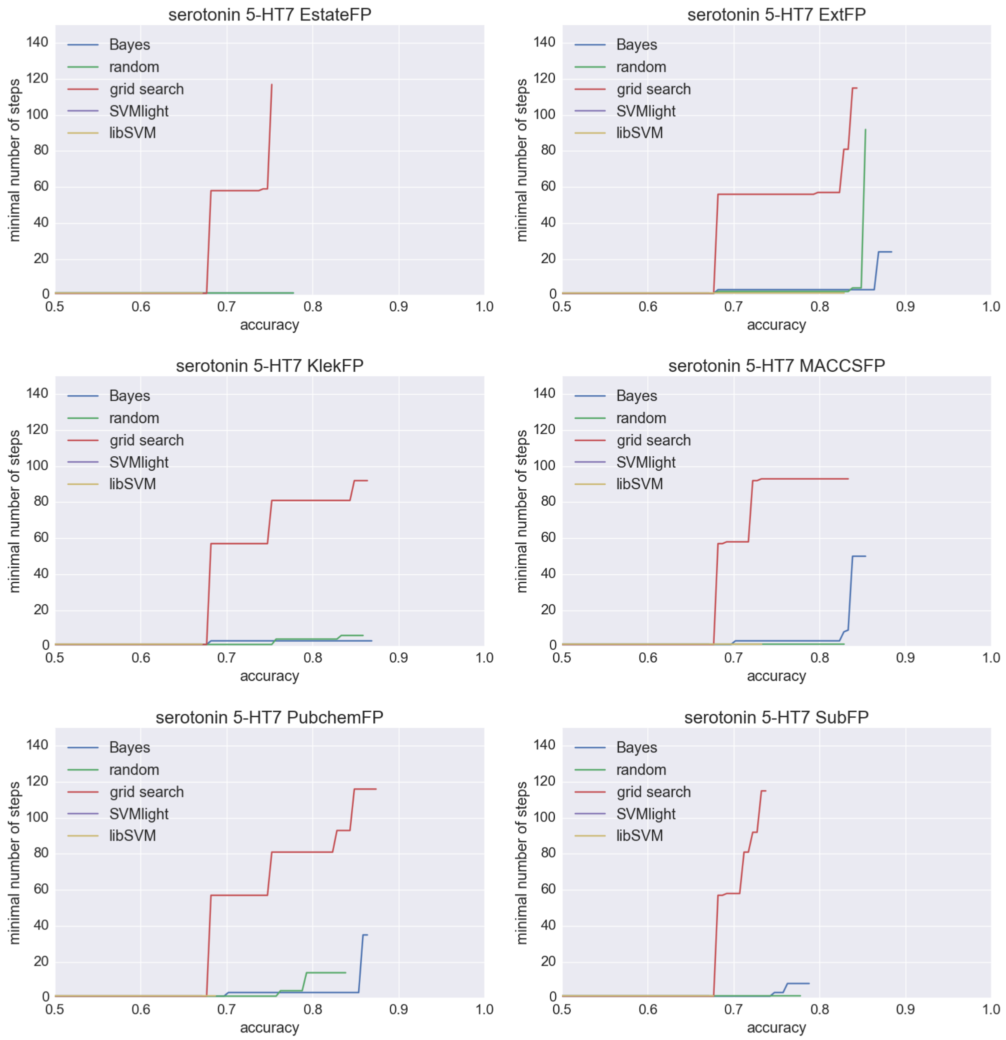

Figure 4: Analysis of the number of iterations of the optimization procedure required for reaching the highest accuracy for 5-HT7 receptor.

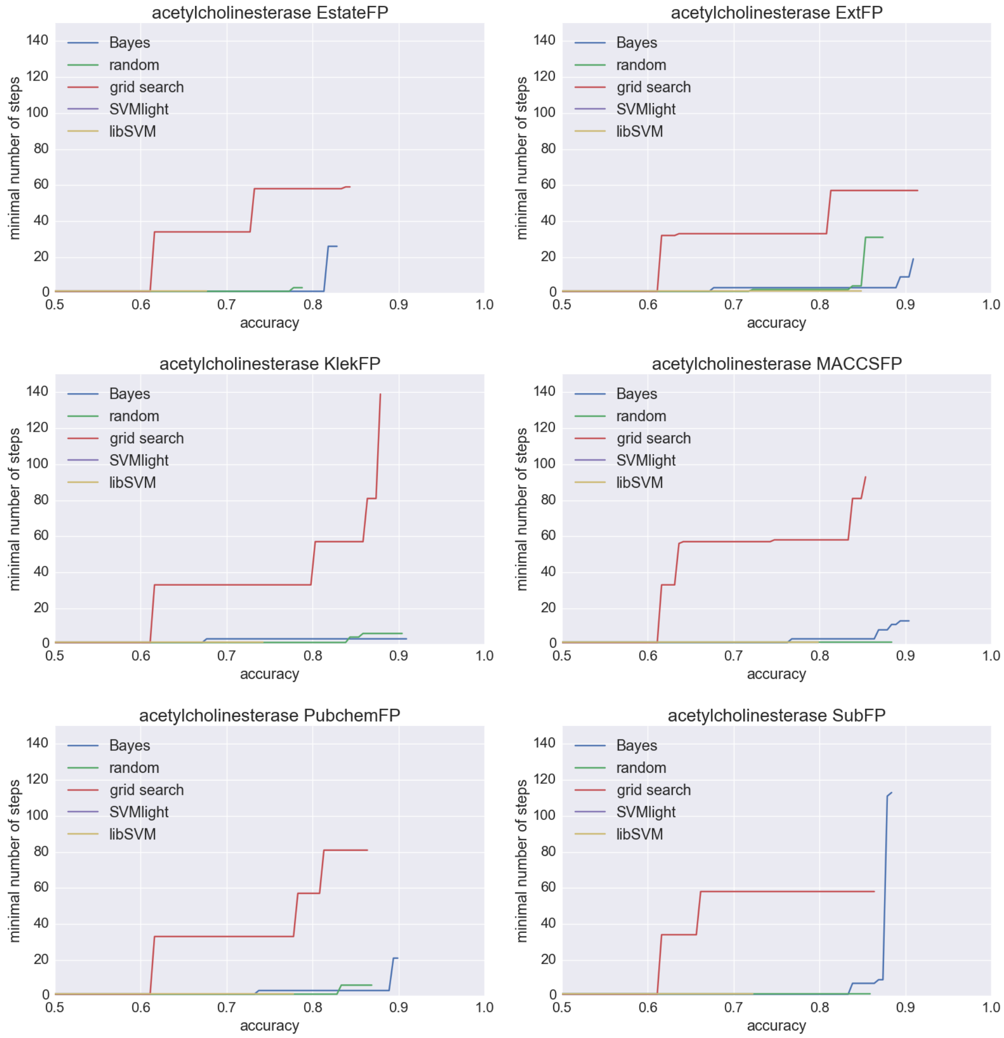

Figure 5: Analysis of the number of iterations of the optimization procedure required for reaching the highest accuracy for acetylcholinesterase.

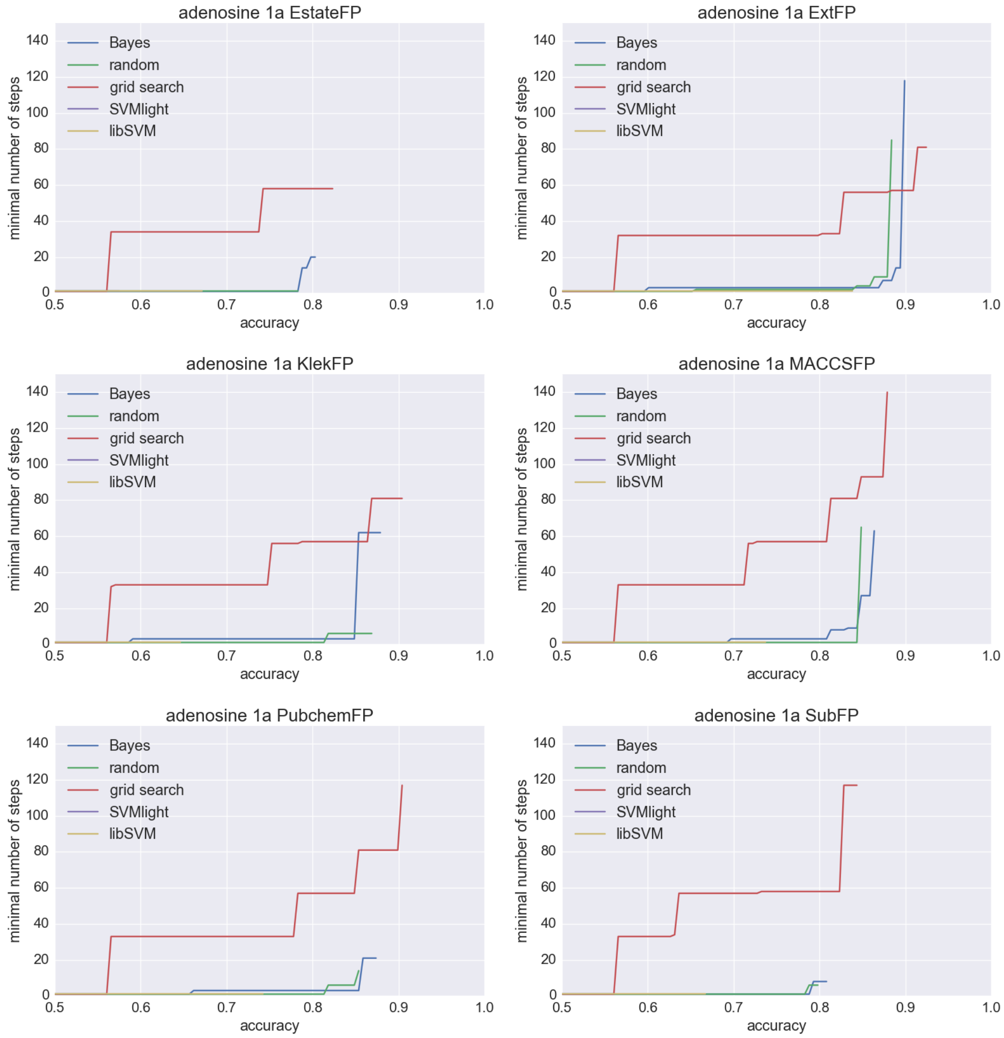

Figure 6: Analysis of the number of iterations of the optimization procedure required for reaching the highest accuracy for adenosine 1a receptor.

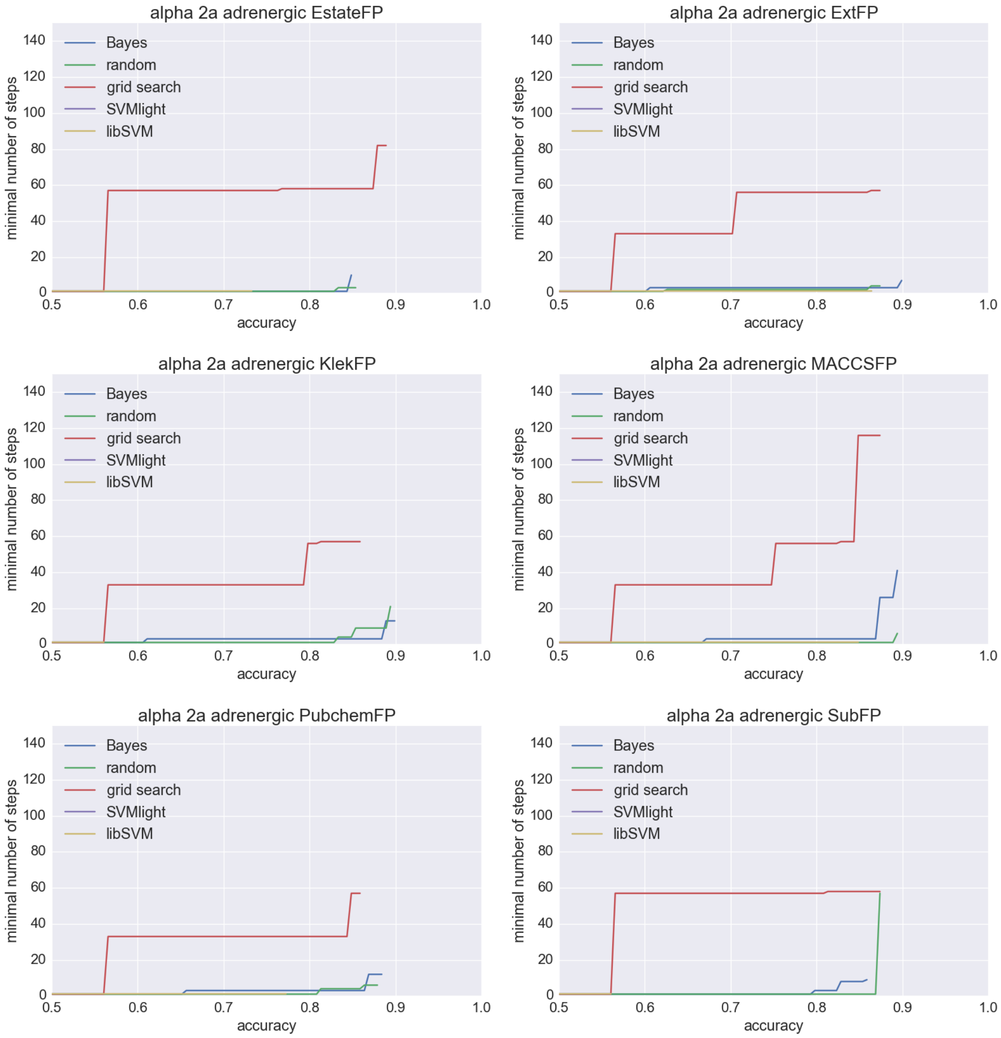

Figure 7: Analysis of the number of iterations of the optimization procedure required for reaching the highest accuracy for alpha 2a AR.

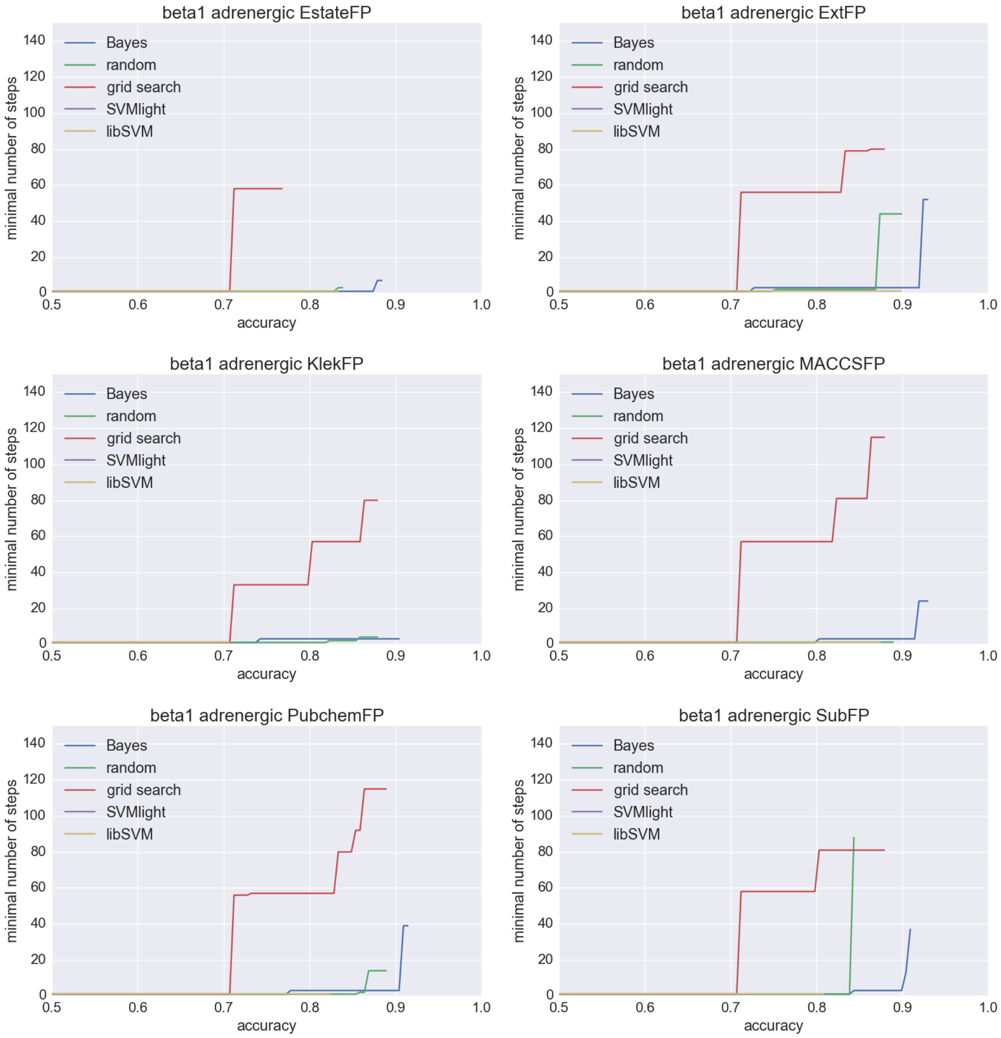

Figure 8: Analysis of the number of iterations of the optimization procedure required for reaching the highest accuracy for beta1AR.

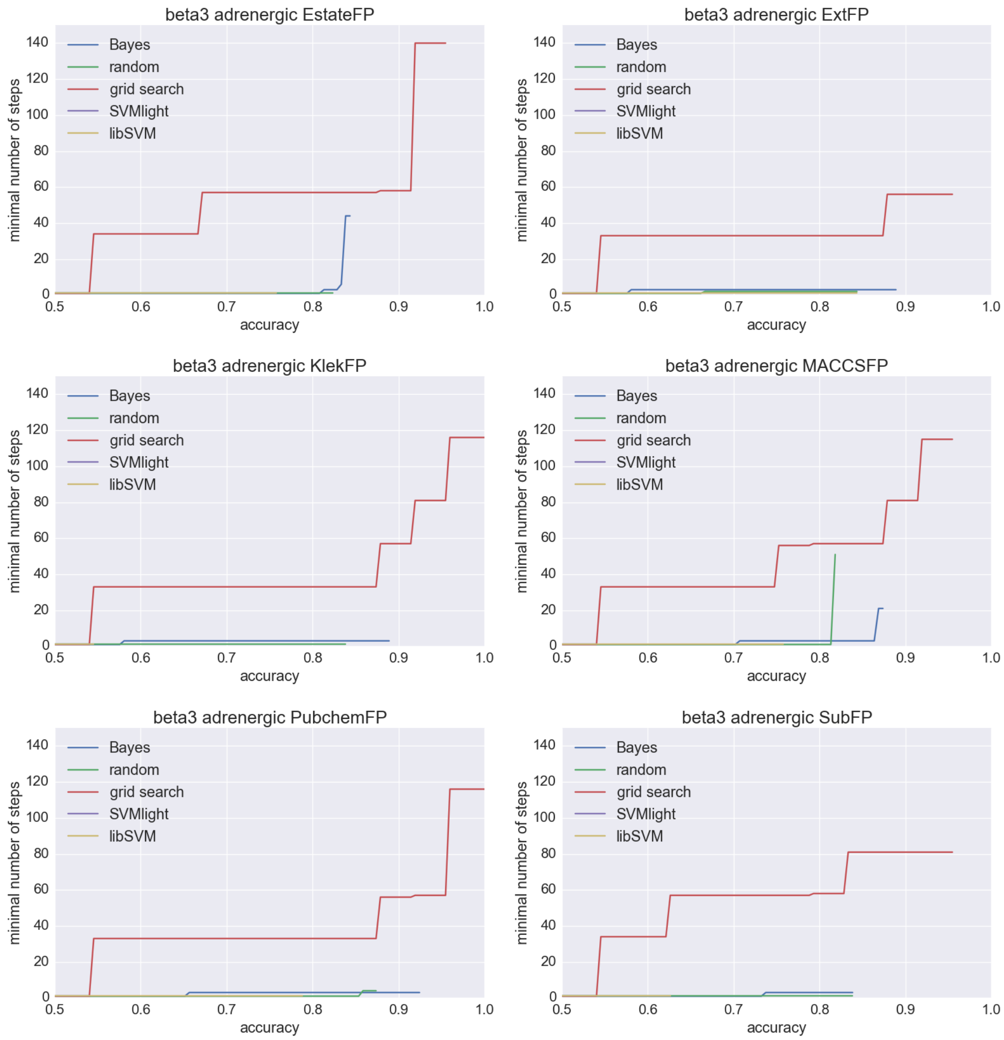

Figure 9: Analysis of the number of iterations of the optimization procedure required for reaching the highest accuracy for beta3AR.

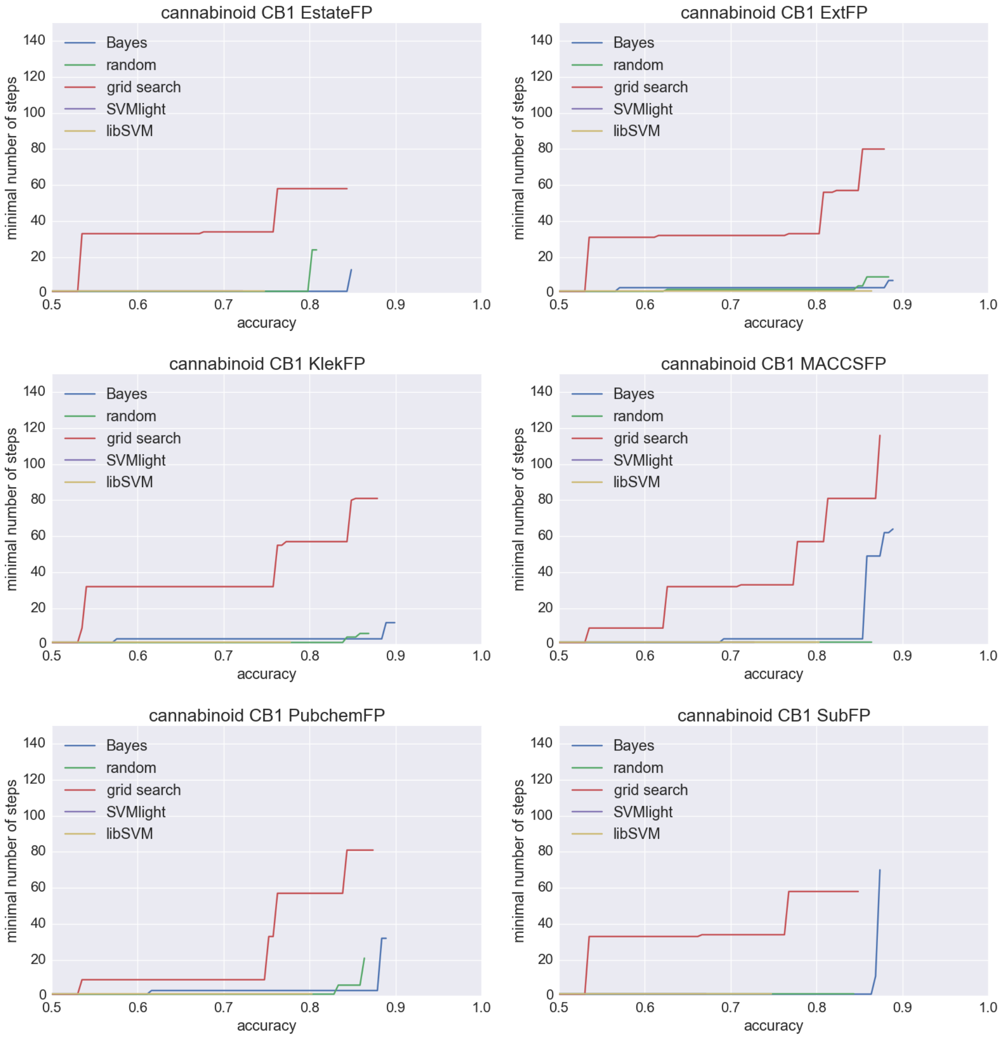

Figure 10: Analysis of the number of iterations of the optimization procedure required for reaching the highest accuracy for cannabinoid CB1 receptor.

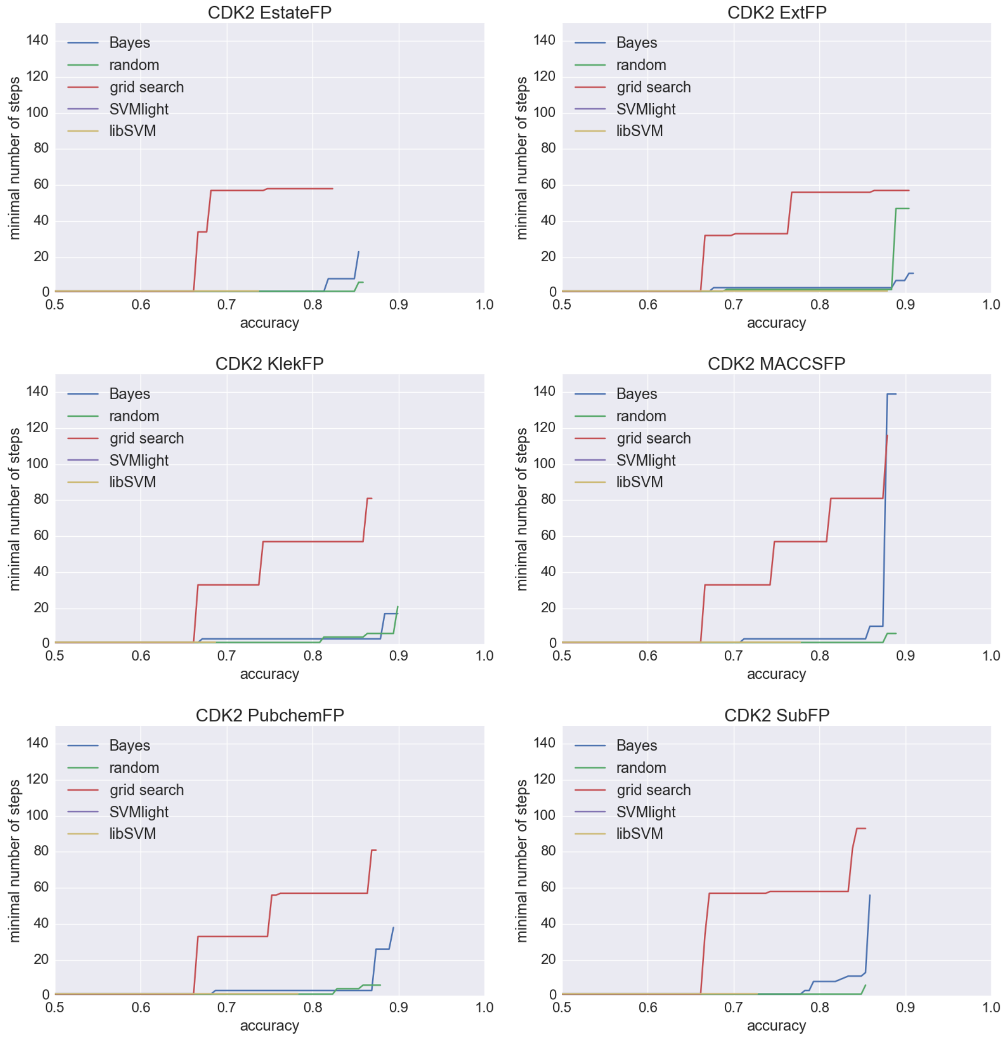

Figure 11: Analysis of the number of iterations of the optimization procedure required for reaching the highest accuracy for CDK2.

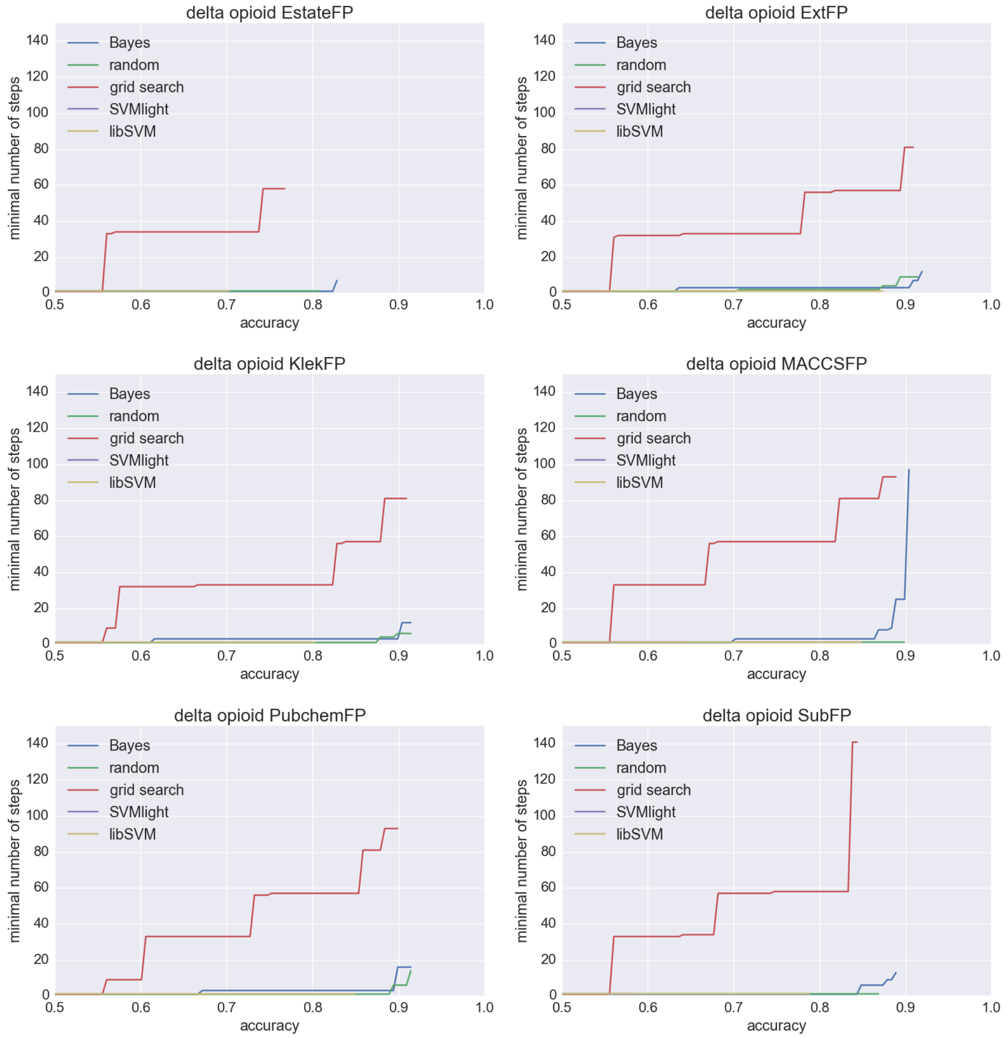

Figure 12: Analysis of the number of iterations of the optimization procedure required for reaching the highest accuracy for delta opioid receptor.

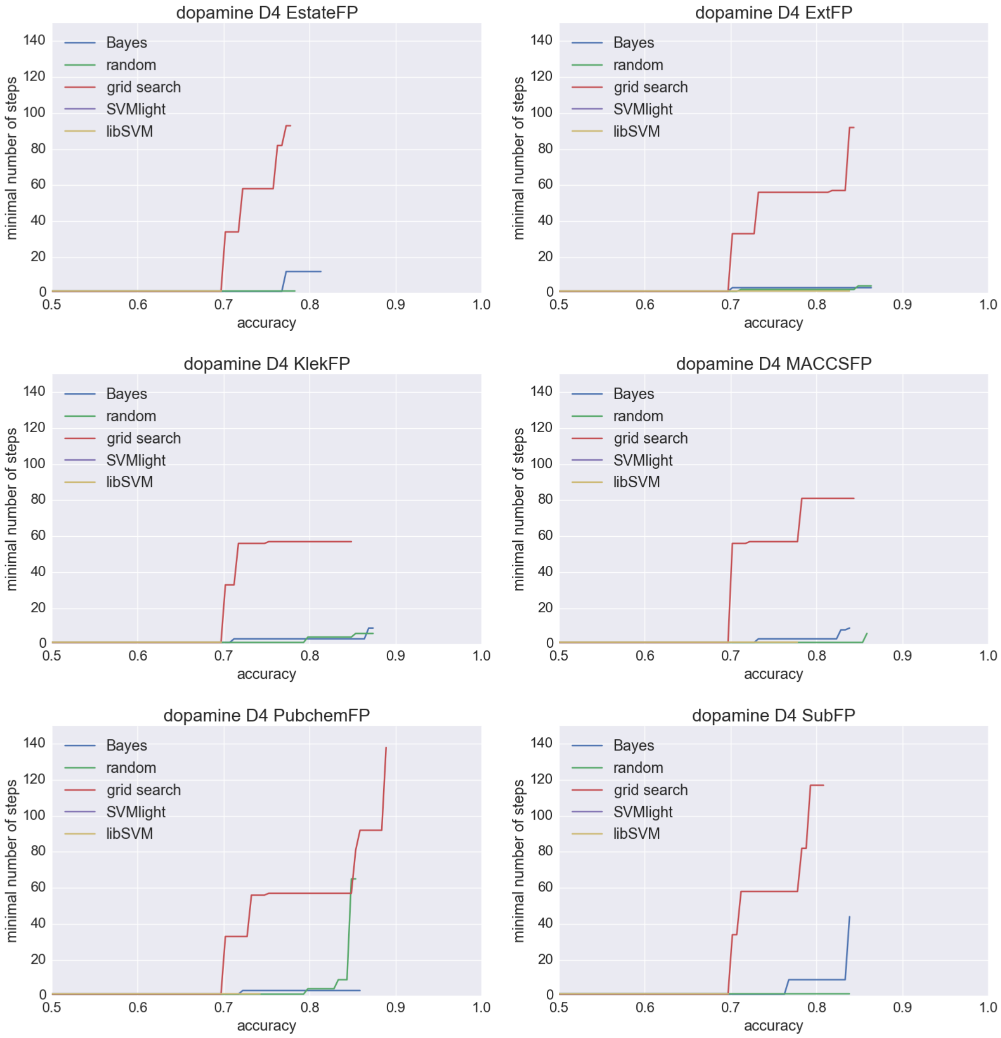

Figure 13: Analysis of the number of iterations of the optimization procedure required for reaching the highest accuracy for dopamine D4 receptor.

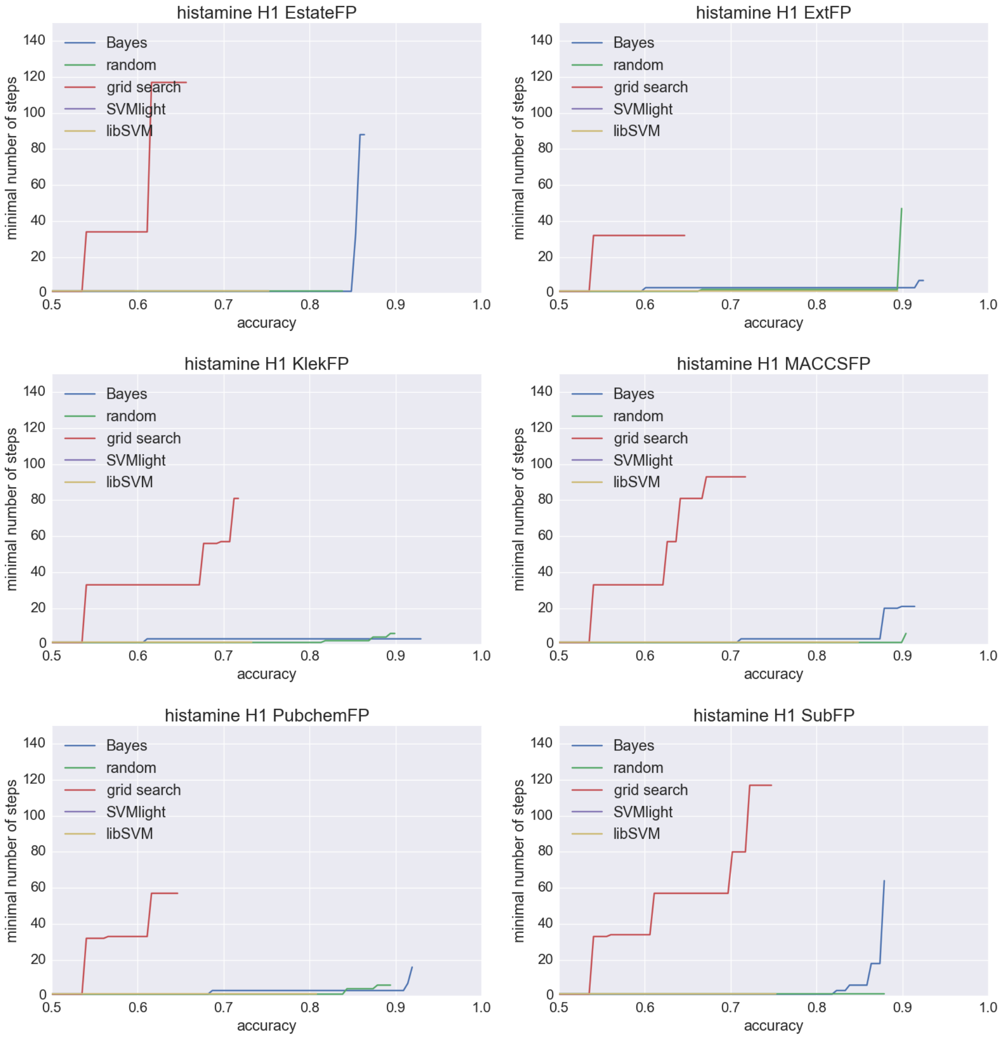

Figure 14: Analysis of the number of iterations of the optimization procedure required for reaching the highest accuracy for histamine H1 receptor.

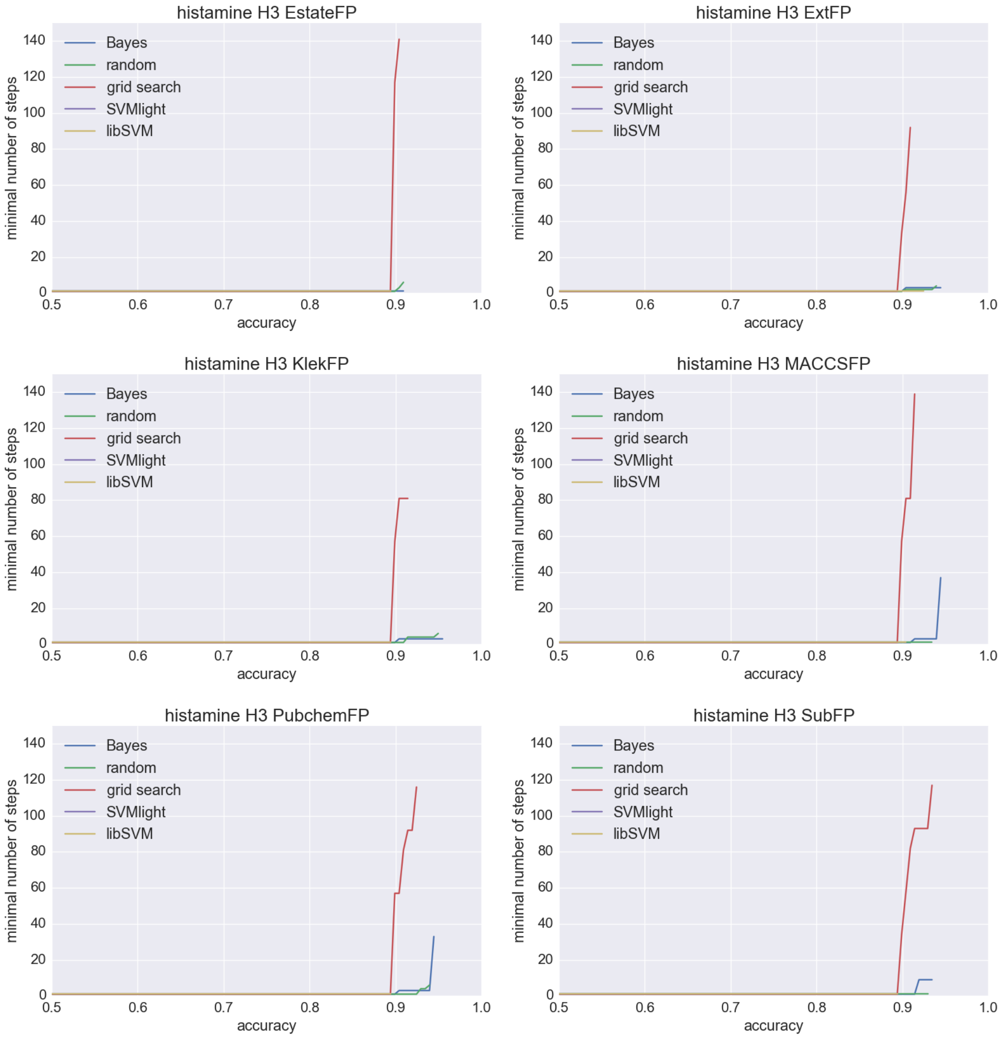

Figure 15: Analysis of the number of iterations of the optimization procedure required for reaching the highest accuracy for histamine H3 receptor.

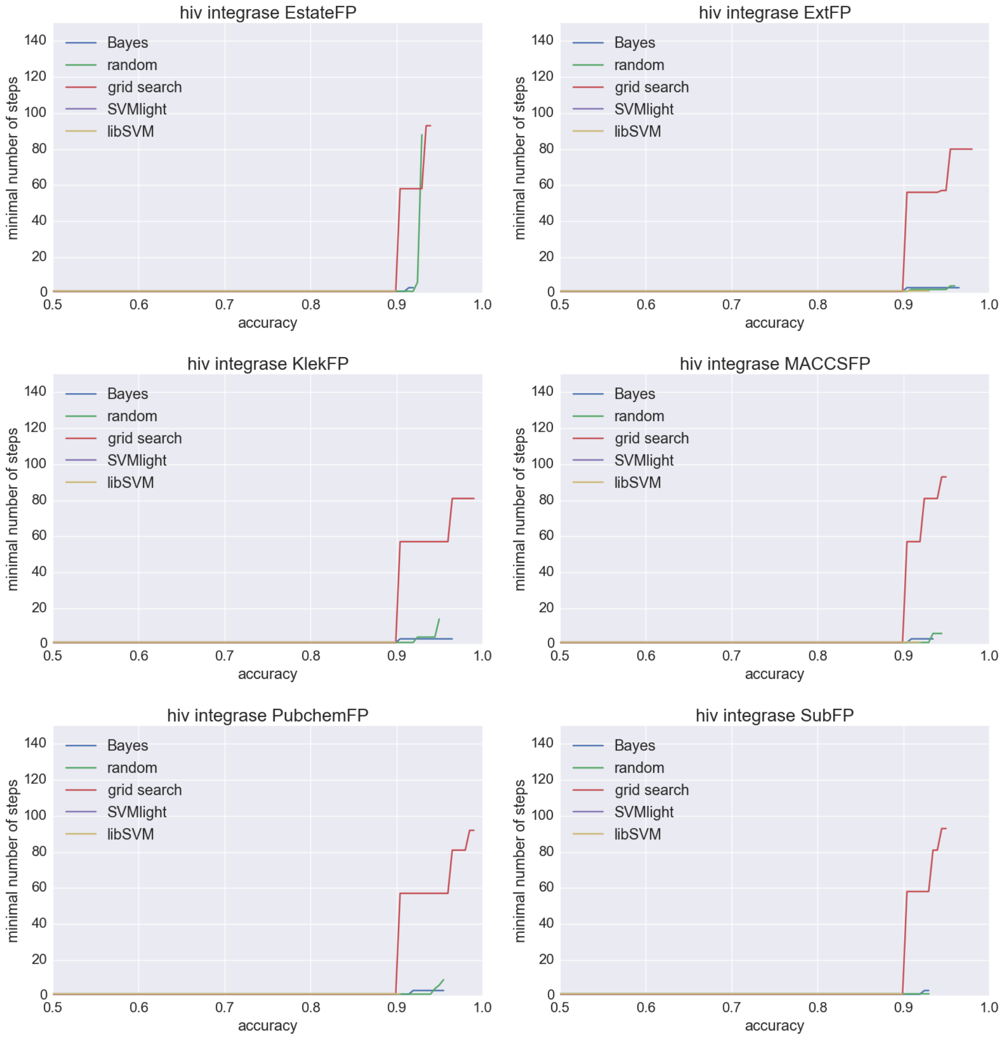

Figure 16: Analysis of the number of iterations of the optimization procedure required for reaching the highest accuracy for HIV integrase.

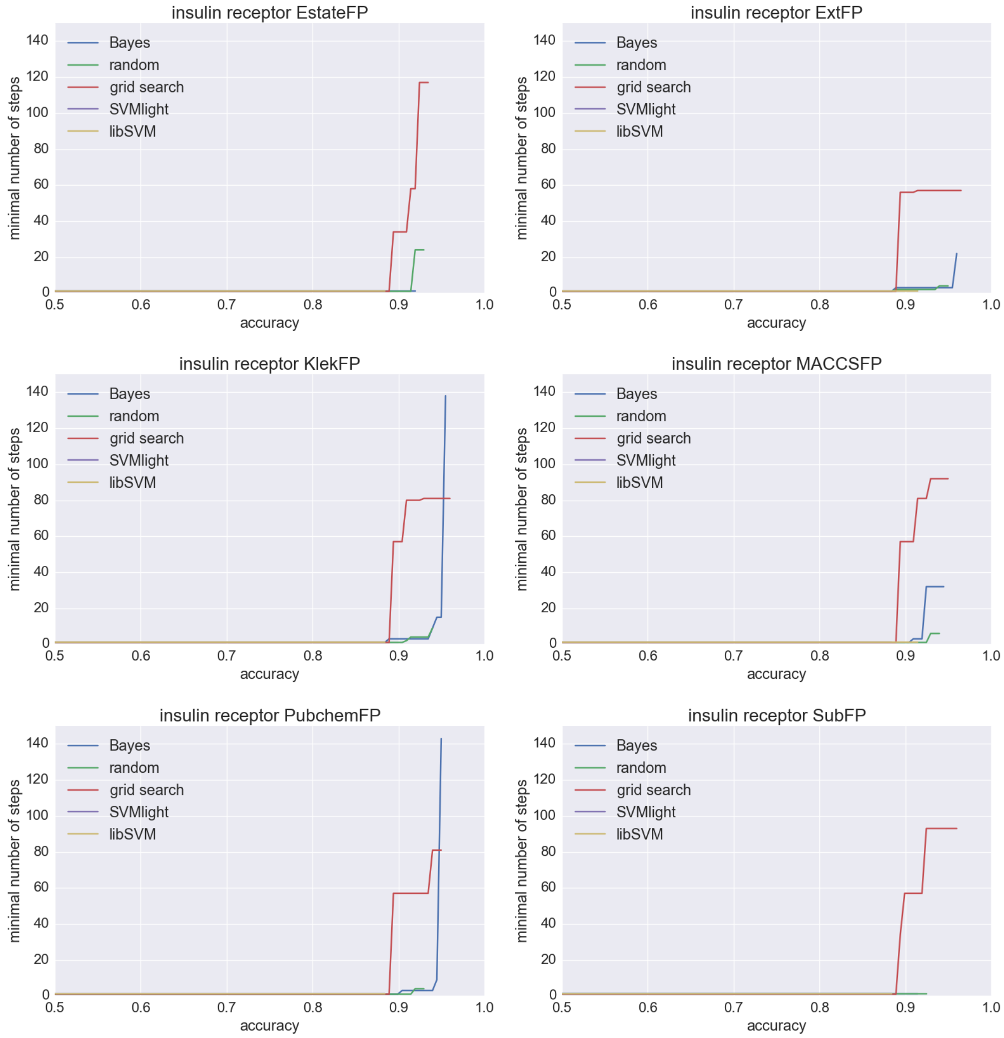

Figure 17: Analysis of the number of iterations of the optimization procedure required for reaching the highest accuracy for insulin receptor.

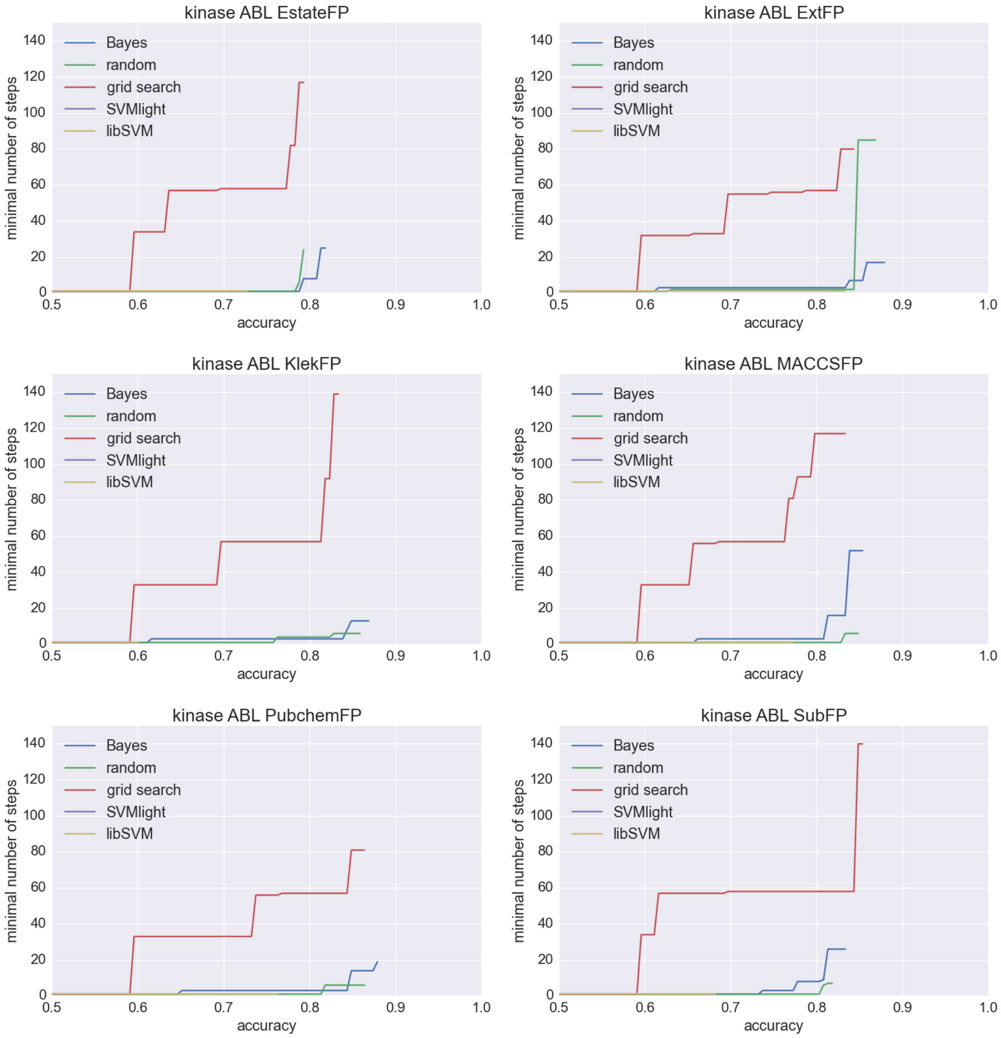

Figure 18: Analysis of the number of iterations of the optimization procedure required for reaching the highest accuracy for kinase ABL.

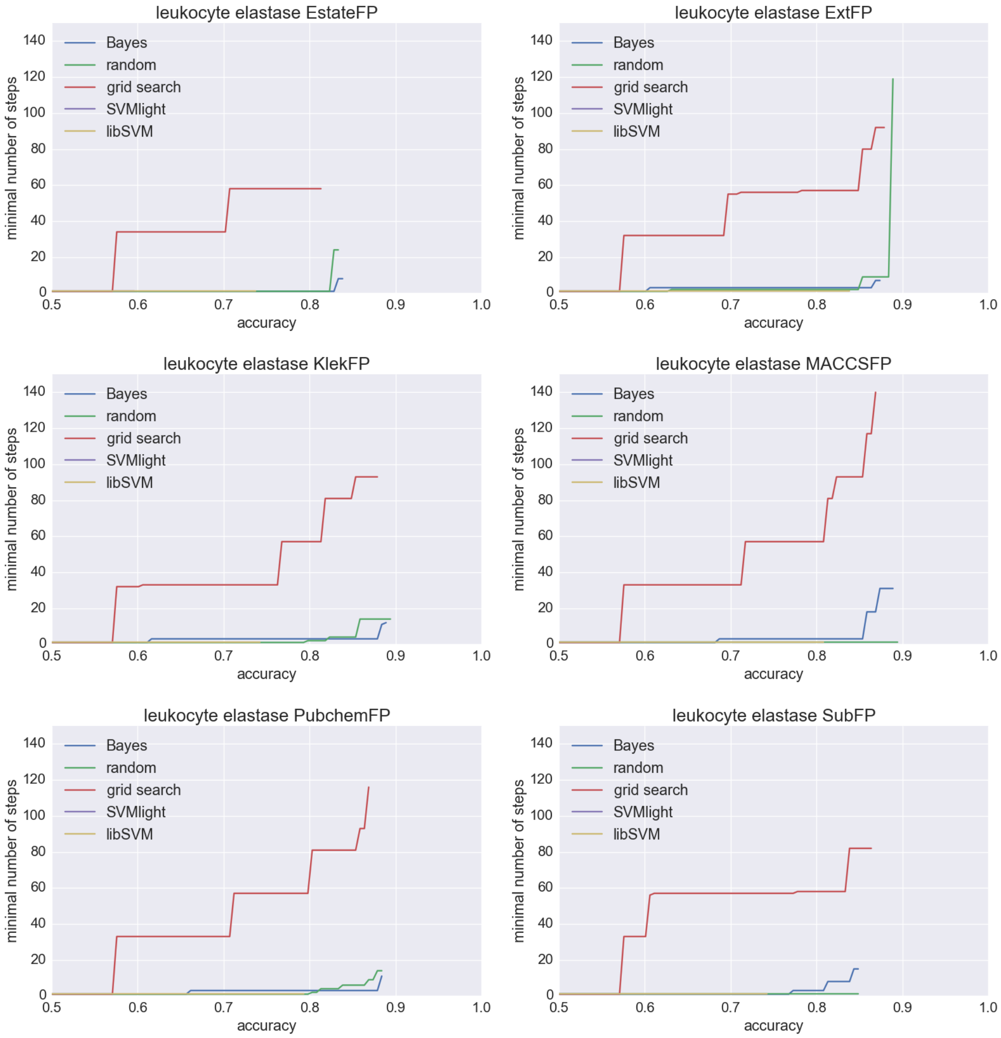

Figure 19: Analysis of the number of iterations of the optimization procedure required for reaching the highest accuracy for leukocyte elastase.

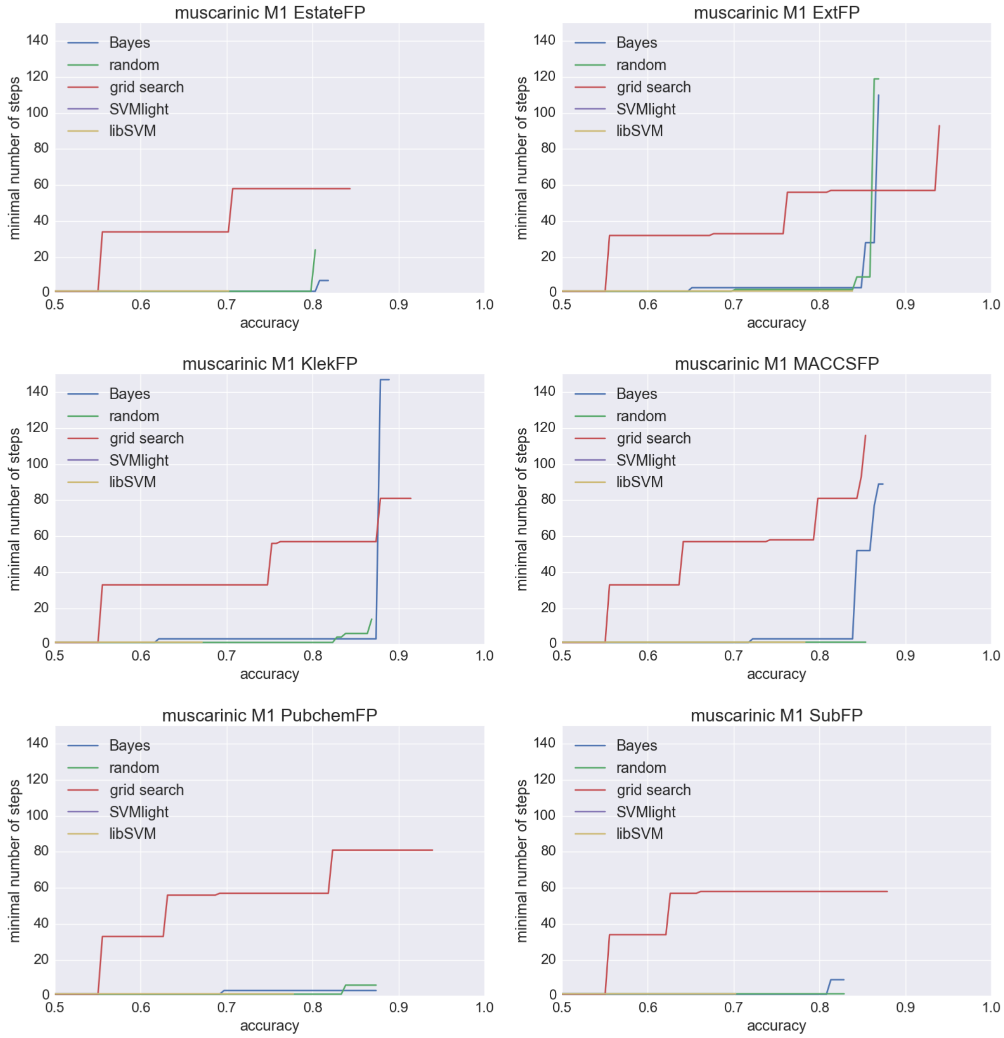

Figure 20: Analysis of the number of iterations of the optimization procedure required for reaching the highest accuracy for muscarininc M1 receptor.

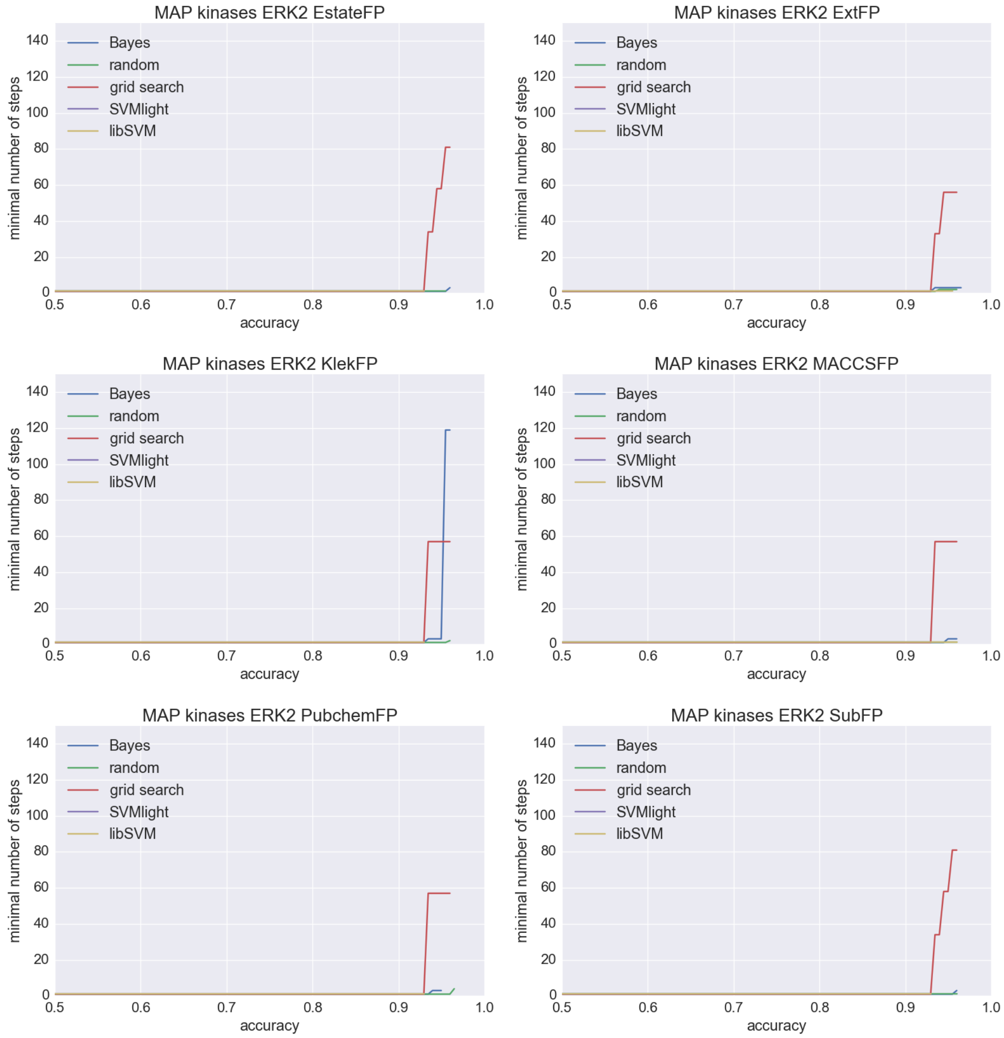

Figure 21: Analysis of the number of iterations of the optimization procedure required for reaching the highest accuracy for MAP kinases ERK2.
