# Supplementary material for: Robust optimization of SVM hyperparameters in the classification of bioactive compounds
Source: J Cheminform. 2015 Aug 14;7:38. doi: 10.1186/s13321-015-0088-0 (PMC4534515; doi:10.1186/s13321-015-0088-0)
Supplement: Additional file 4: — Analysis of the changes of accuracy for different steps for all tested targets. The file presents the set of parameters tested in the subsequent iterations of the optimization procedure with the analysis of classification accuracy values that they were providing. [file 13321_2015_88_MOESM4_ESM.pdf]

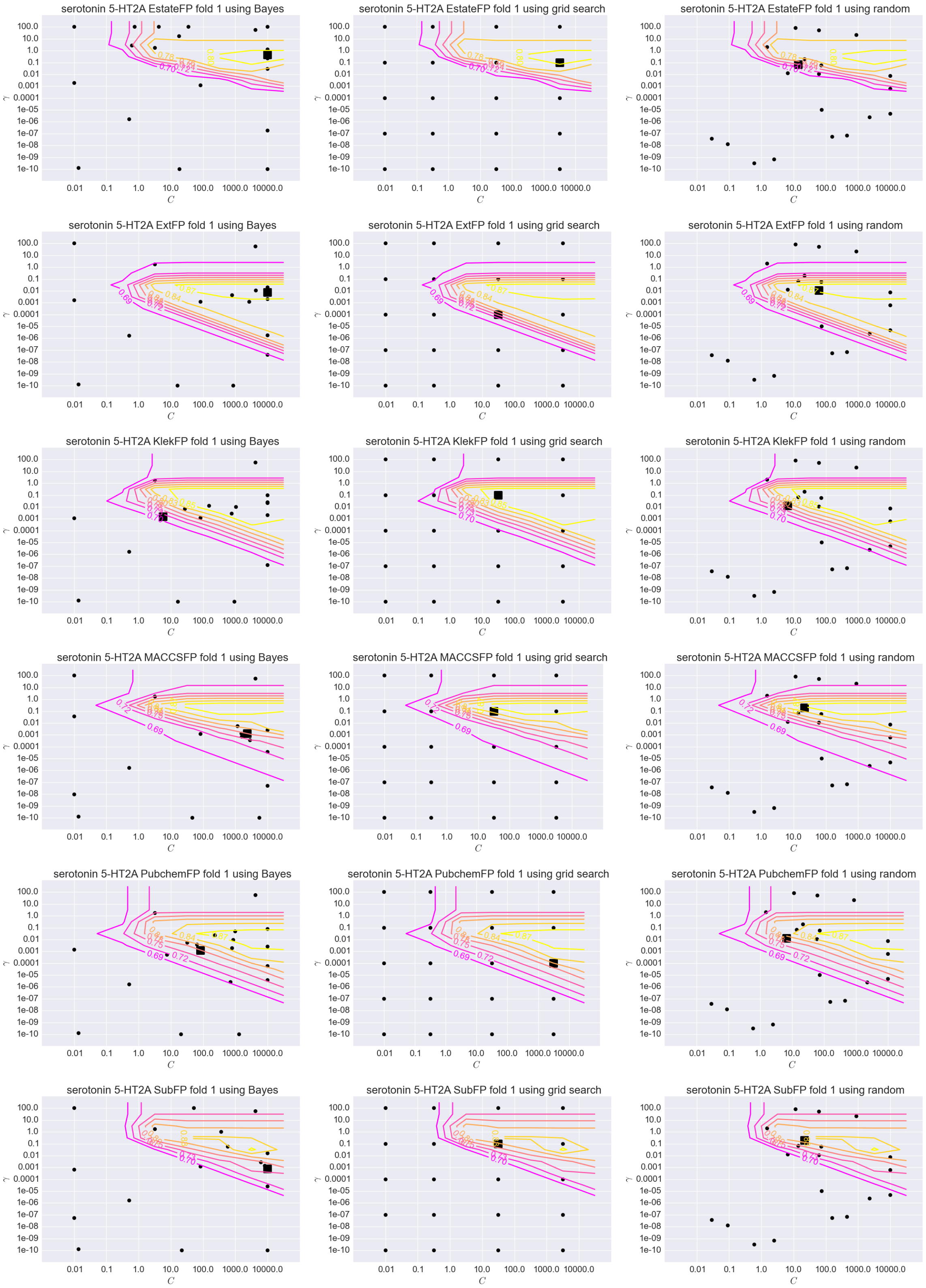

Figure 1: Analysis of the changes in accuracy for different steps for serotonin 5-HT2A receptor.

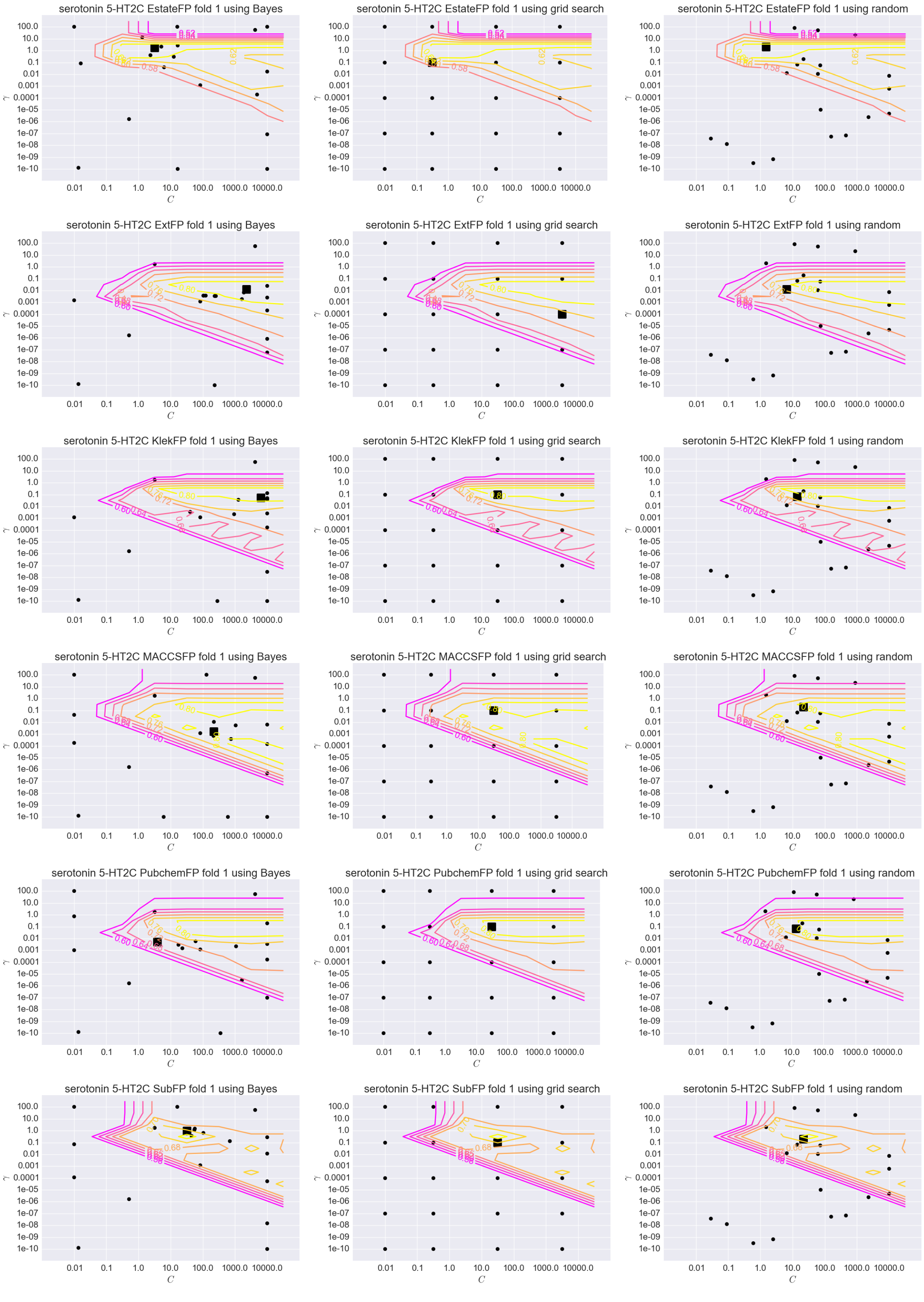

Figure 2: Analysis of the changes in accuracy for different steps for serotonin 5-HT2C receptor.

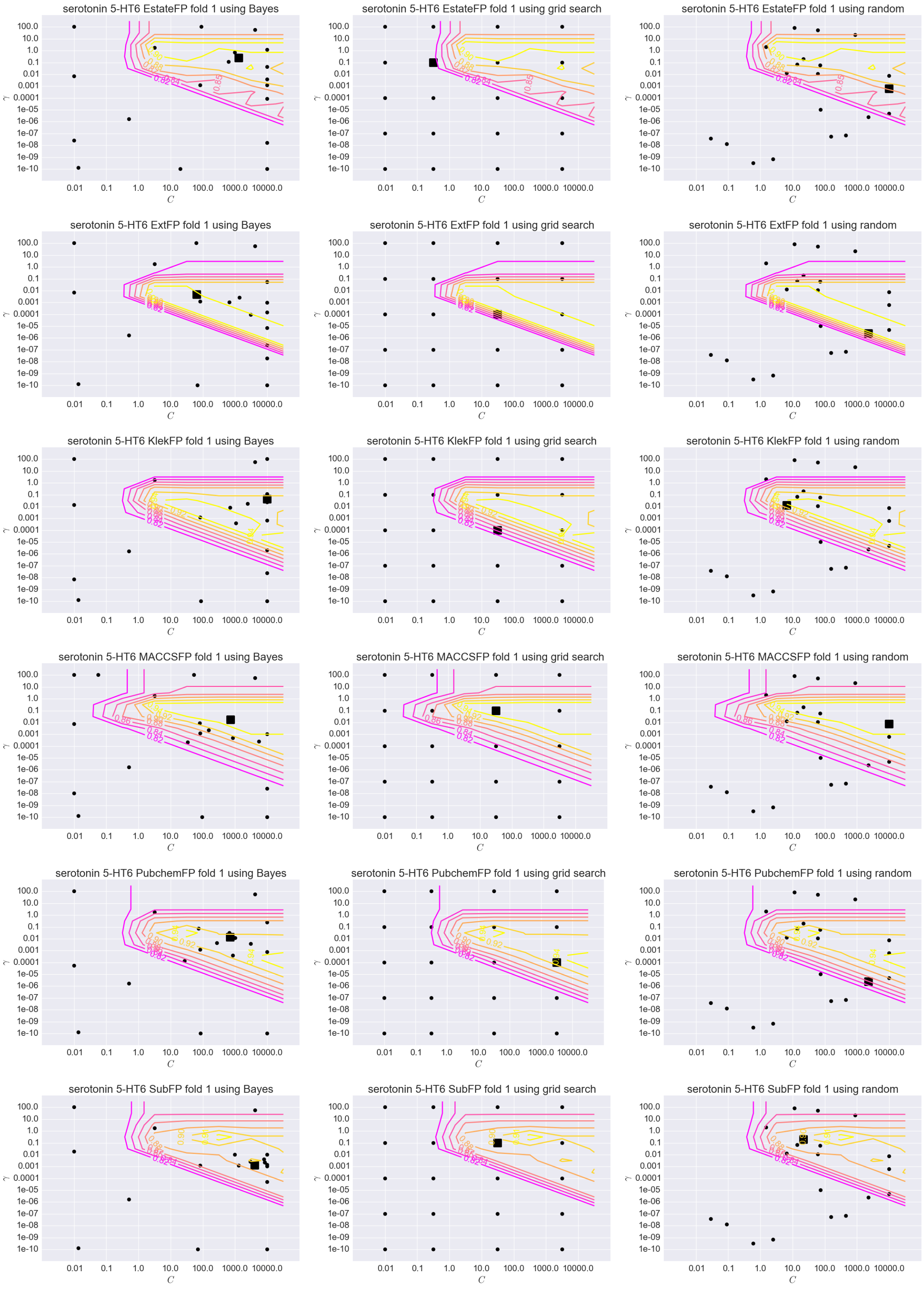

Figure 3: Analysis of the changes in accuracy for different steps for serotonin 5-HT6 receptor.

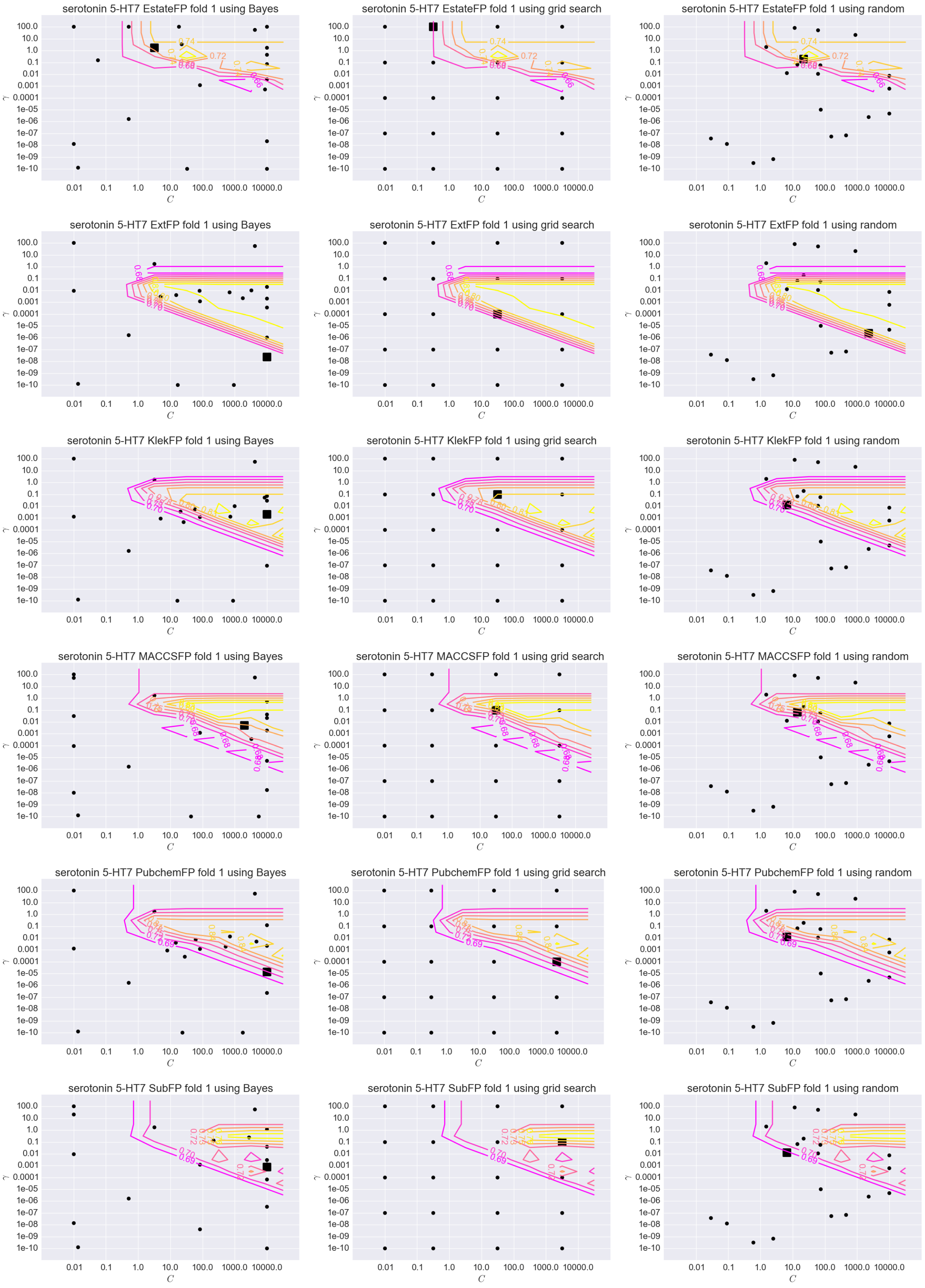

Figure 4: Analysis of the changes in accuracy for different steps for serotonin 5-HT7 receptor.

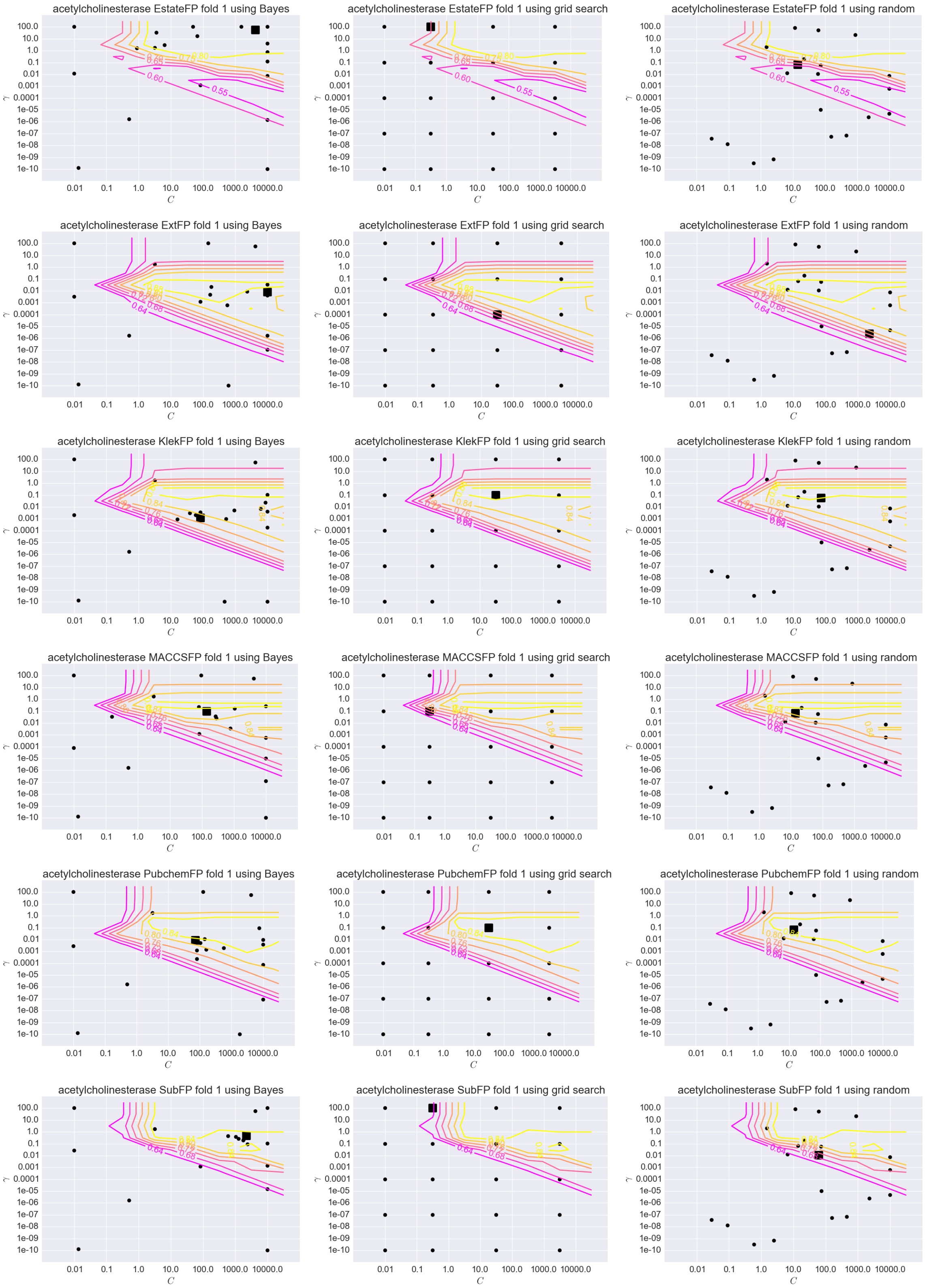

Figure 5: Analysis of the changes in accuracy for different steps for acetylcholinesterase.

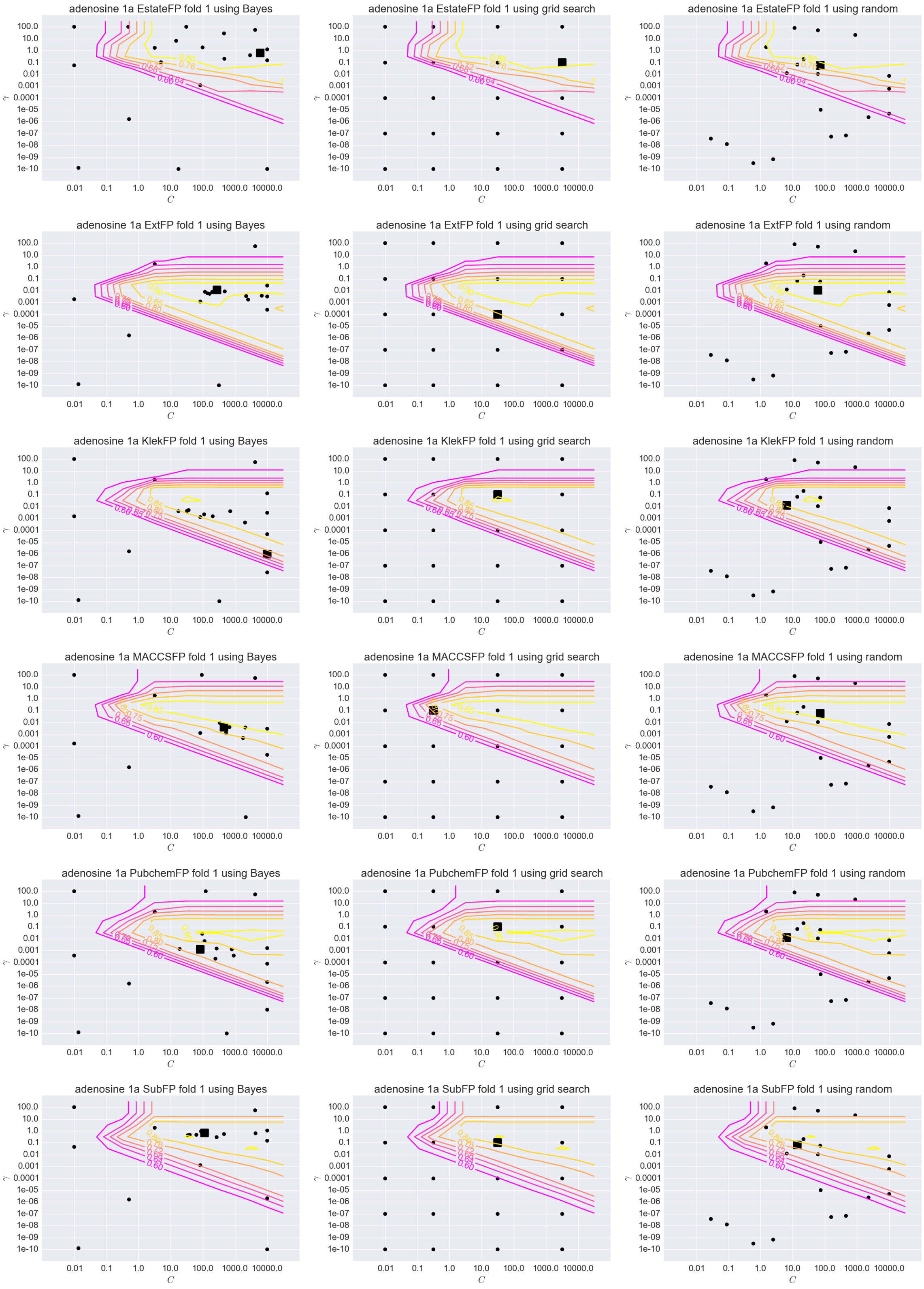

Figure 6: Analysis of the changes in accuracy for different steps for adenosine 1a receptor.

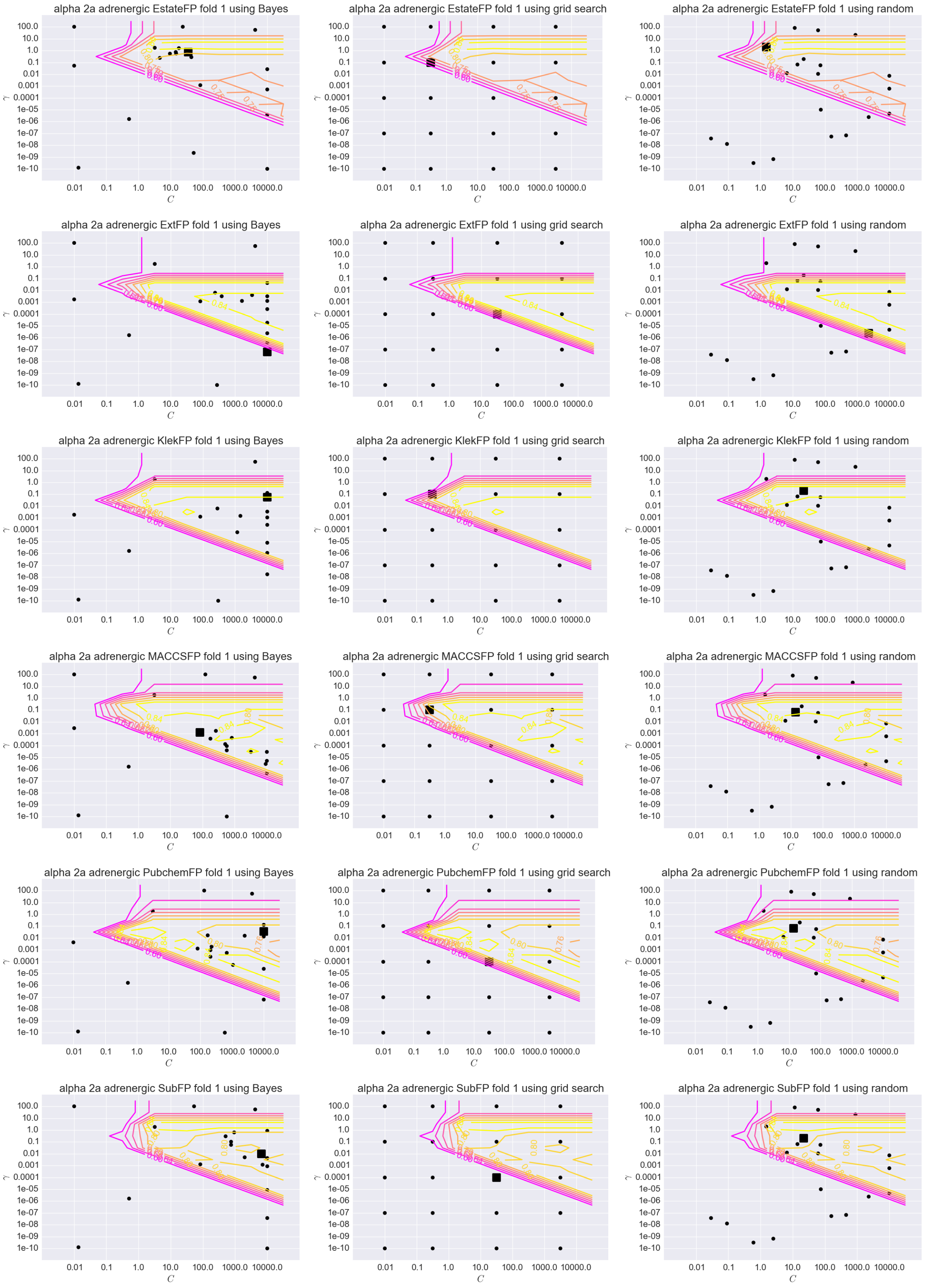

Figure 7: Analysis of the changes in accuracy for different steps for alpha 2a AR.

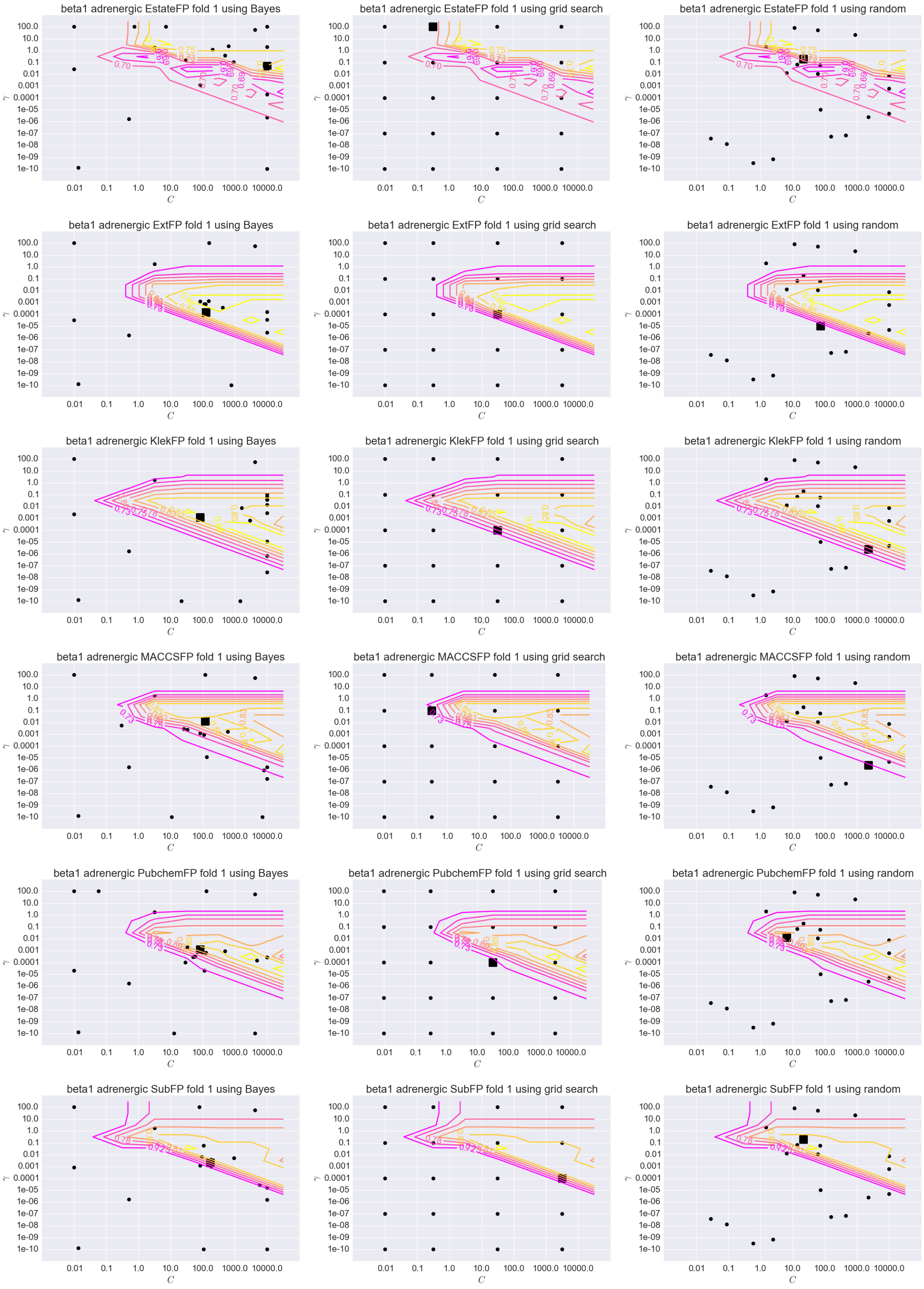

Figure 8: Analysis of the changes in accuracy for different steps for beta1 AR.

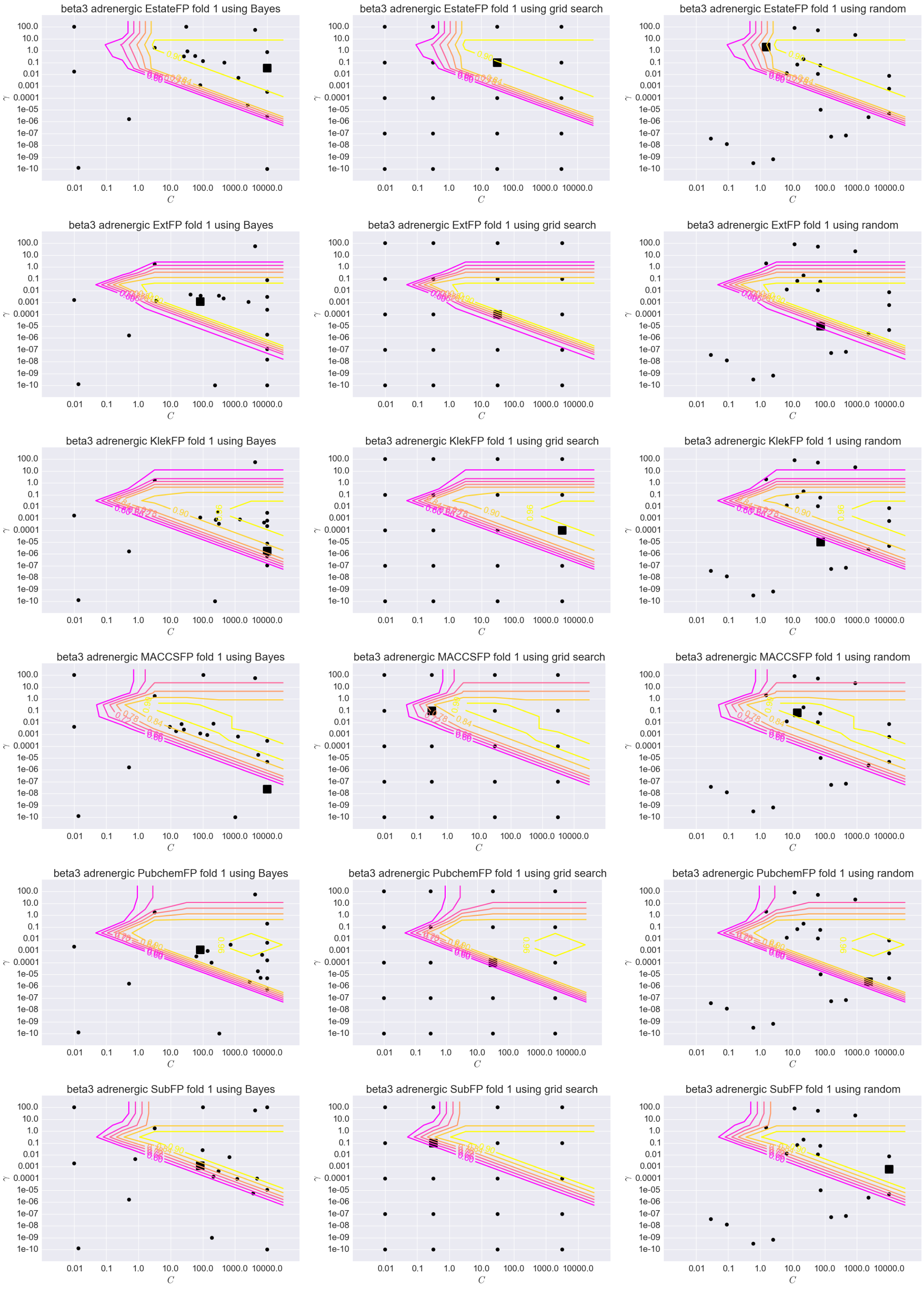

Figure 9: Analysis of the changes in accuracy for different steps for beta3 AR.

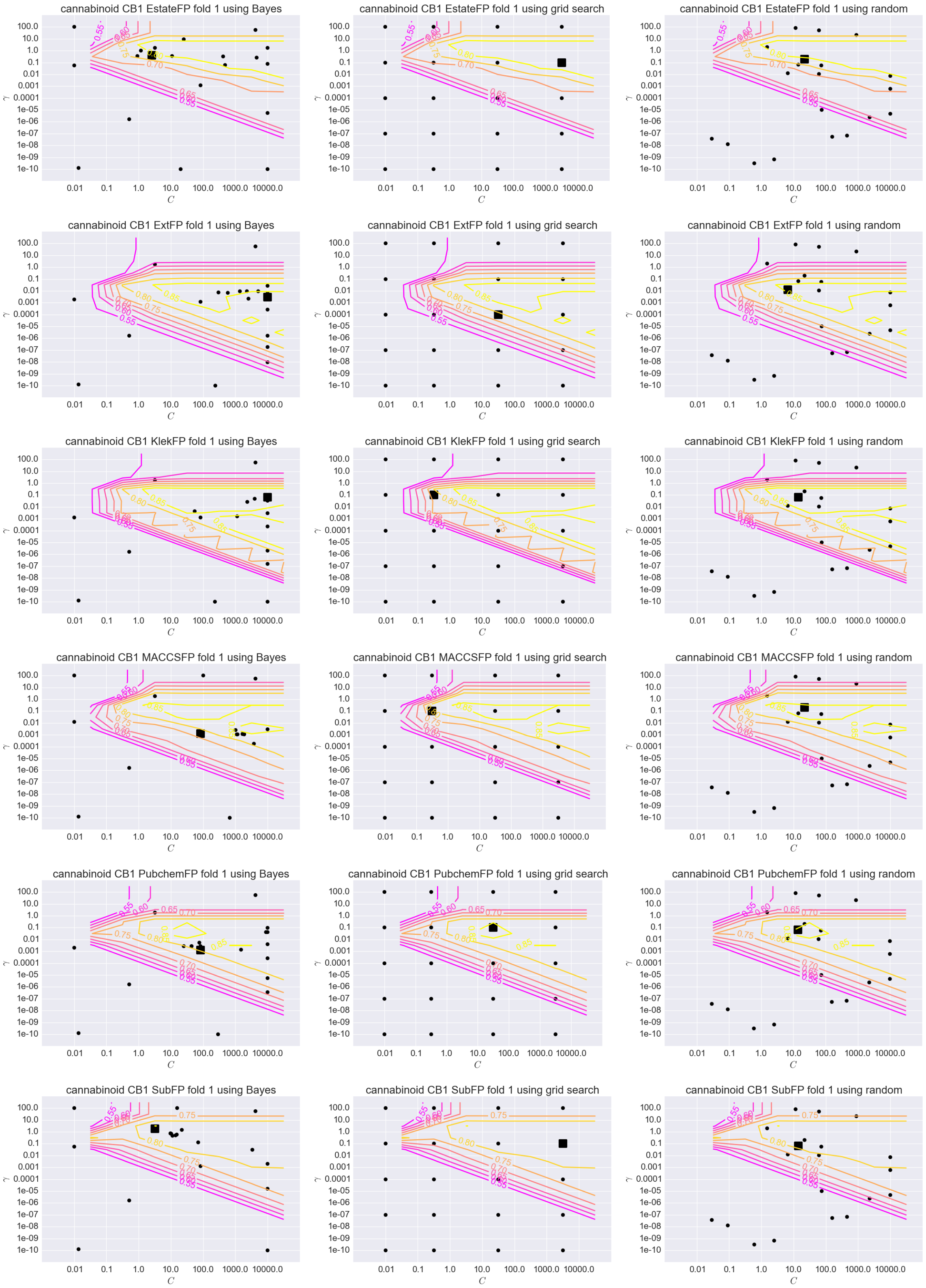

Figure 10: Analysis of the changes in accuracy for different steps for cannabinoid CB1 receptor.

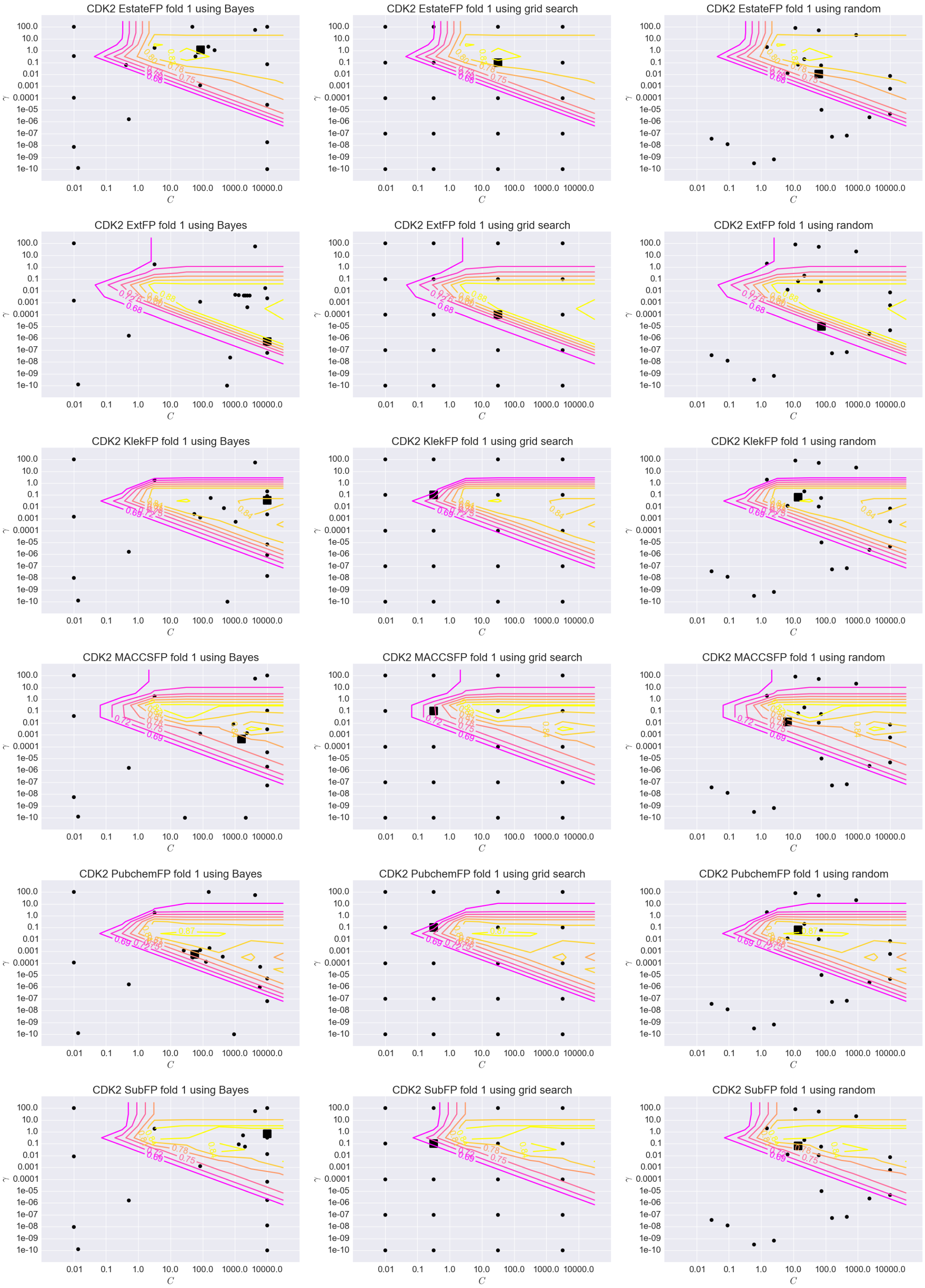

Figure 11: Analysis of the changes in accuracy for different steps for CDK2.

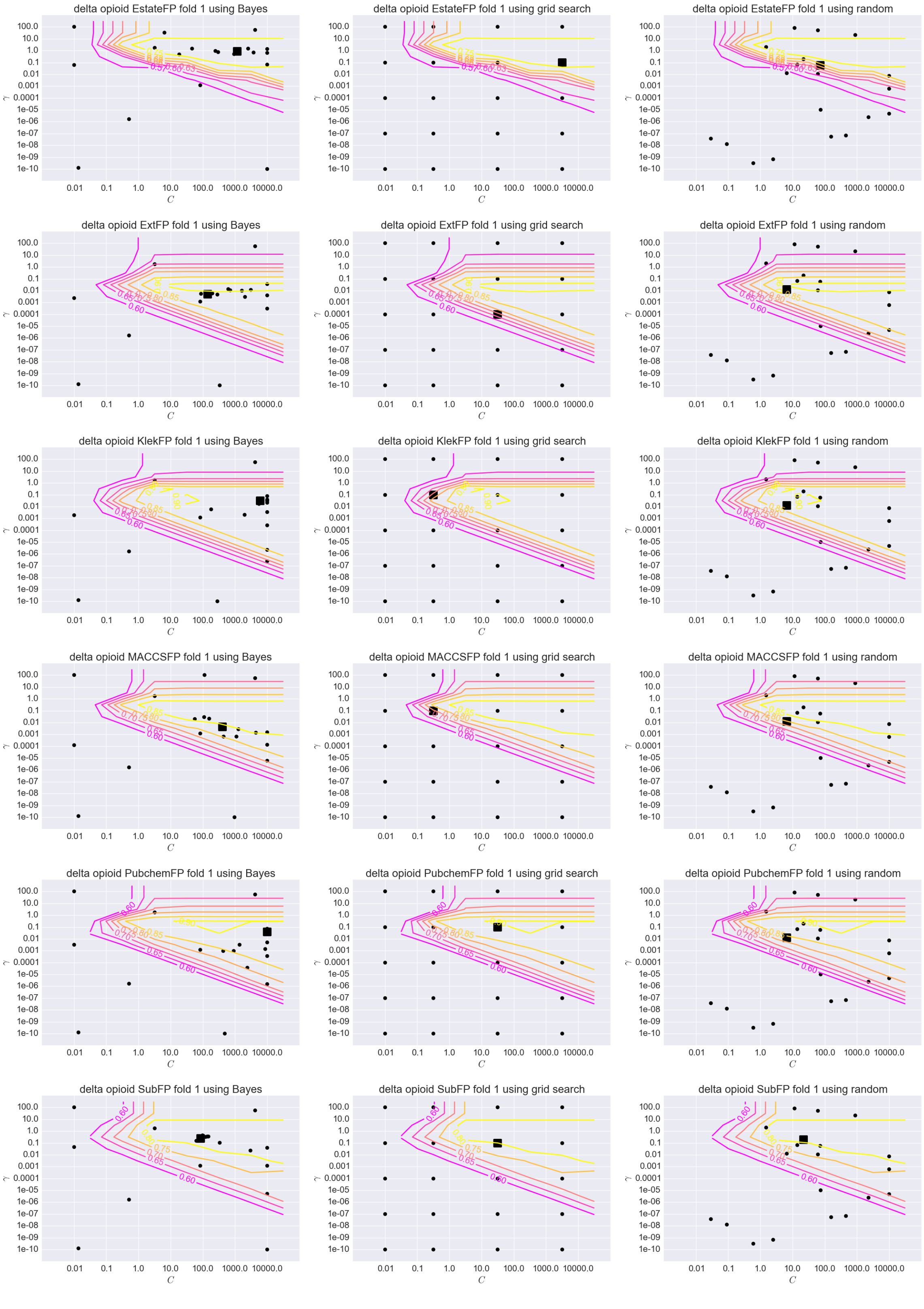

Figure 12: Analysis of the changes in accuracy for different steps for delta opioid receptor.

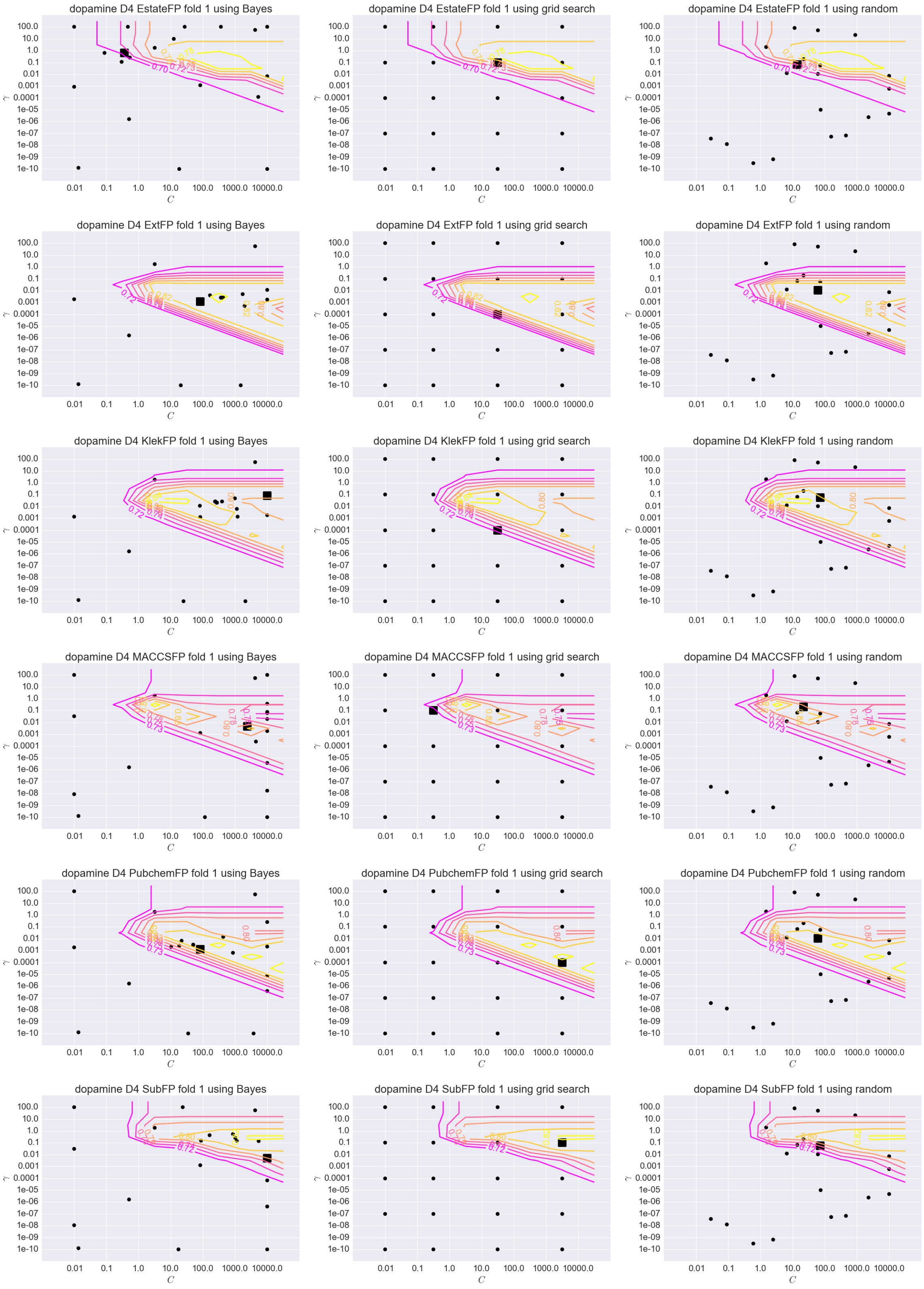

Figure 13: Analysis of the changes in accuracy for different steps for dopamine D4 receptor.

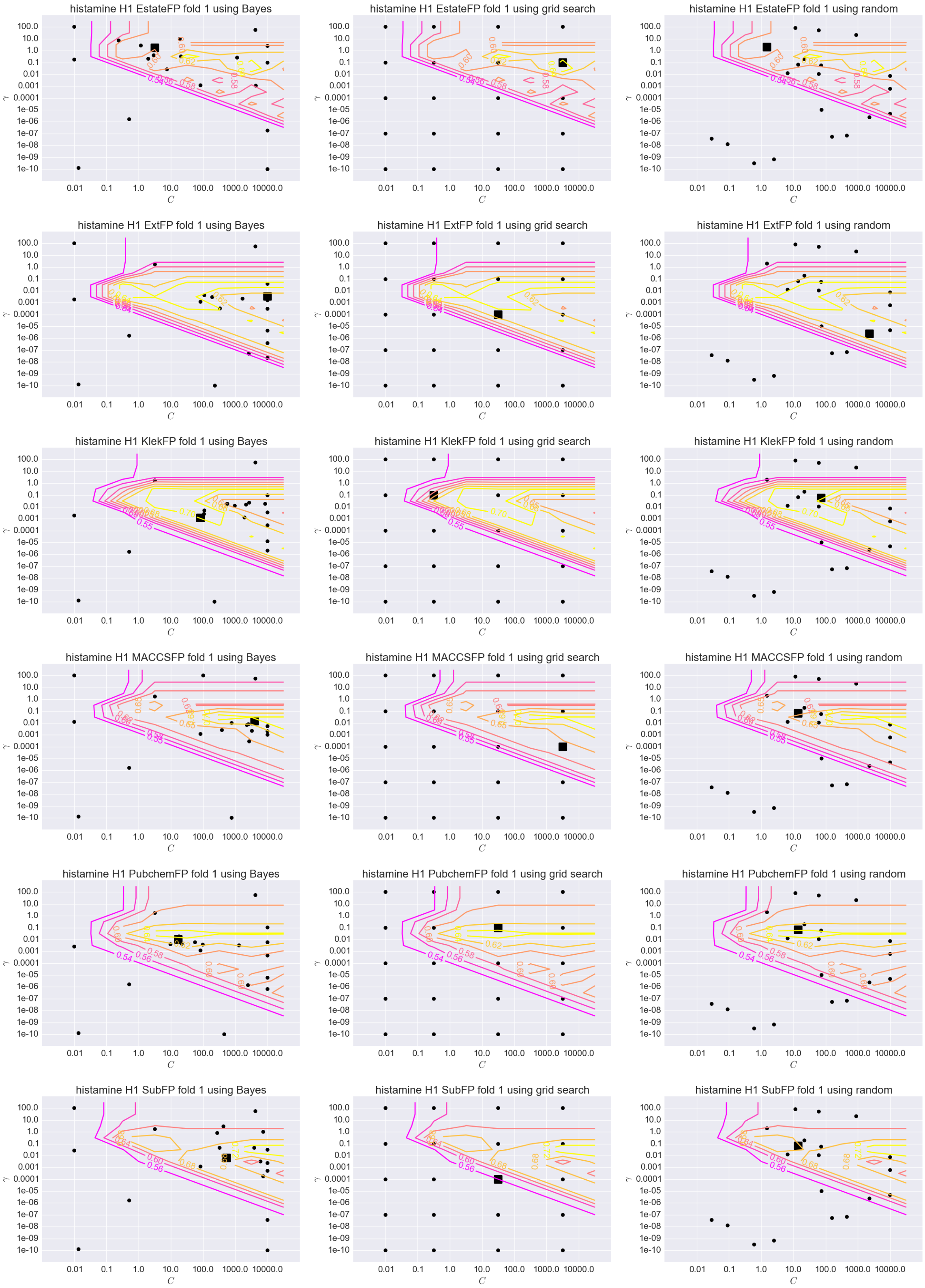

Figure 14: Analysis of the changes in accuracy for different steps for histamine H1 receptor.

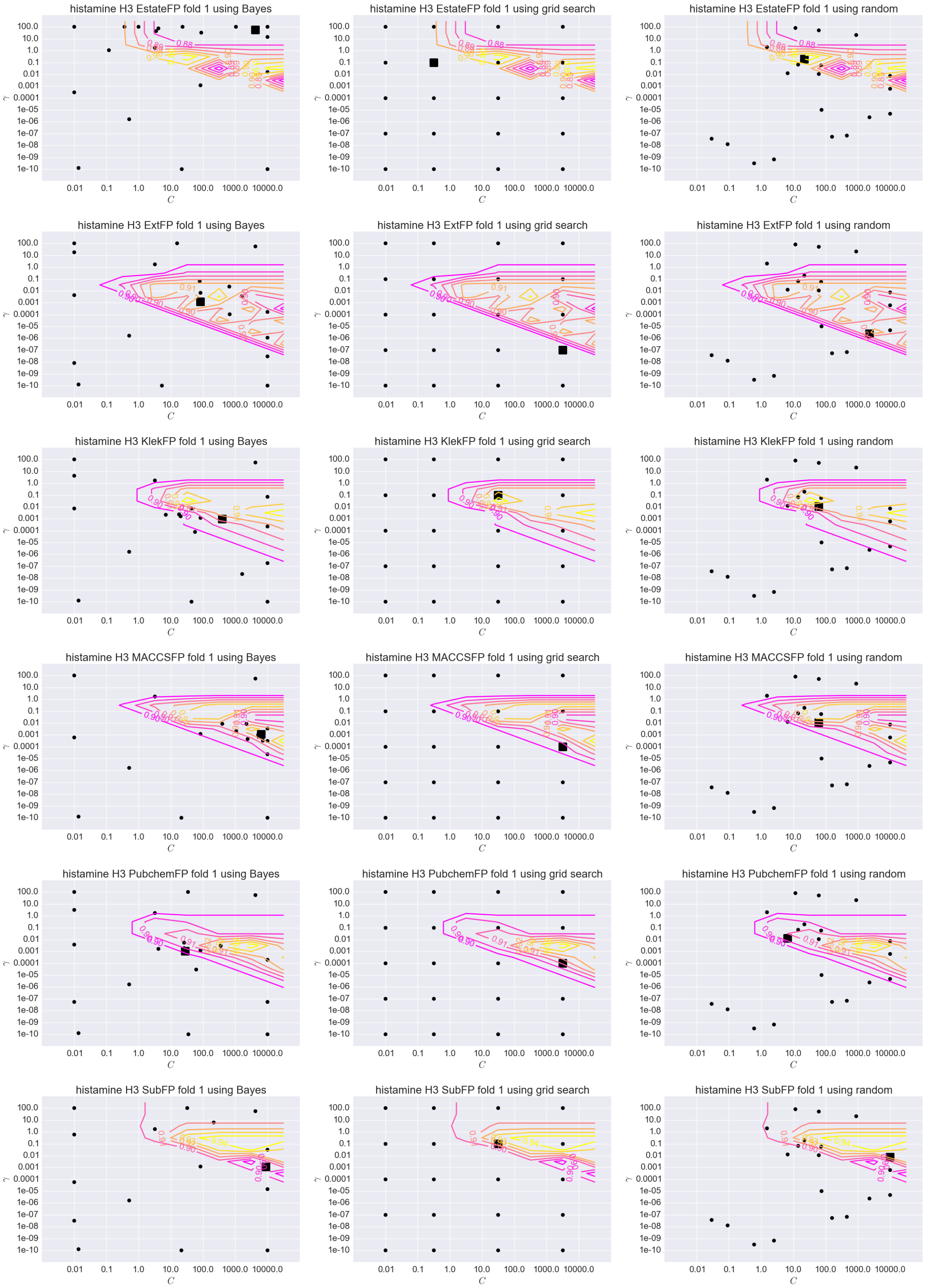

Figure 15: Analysis of the changes in accuracy for different steps for histamine H3 receptor.

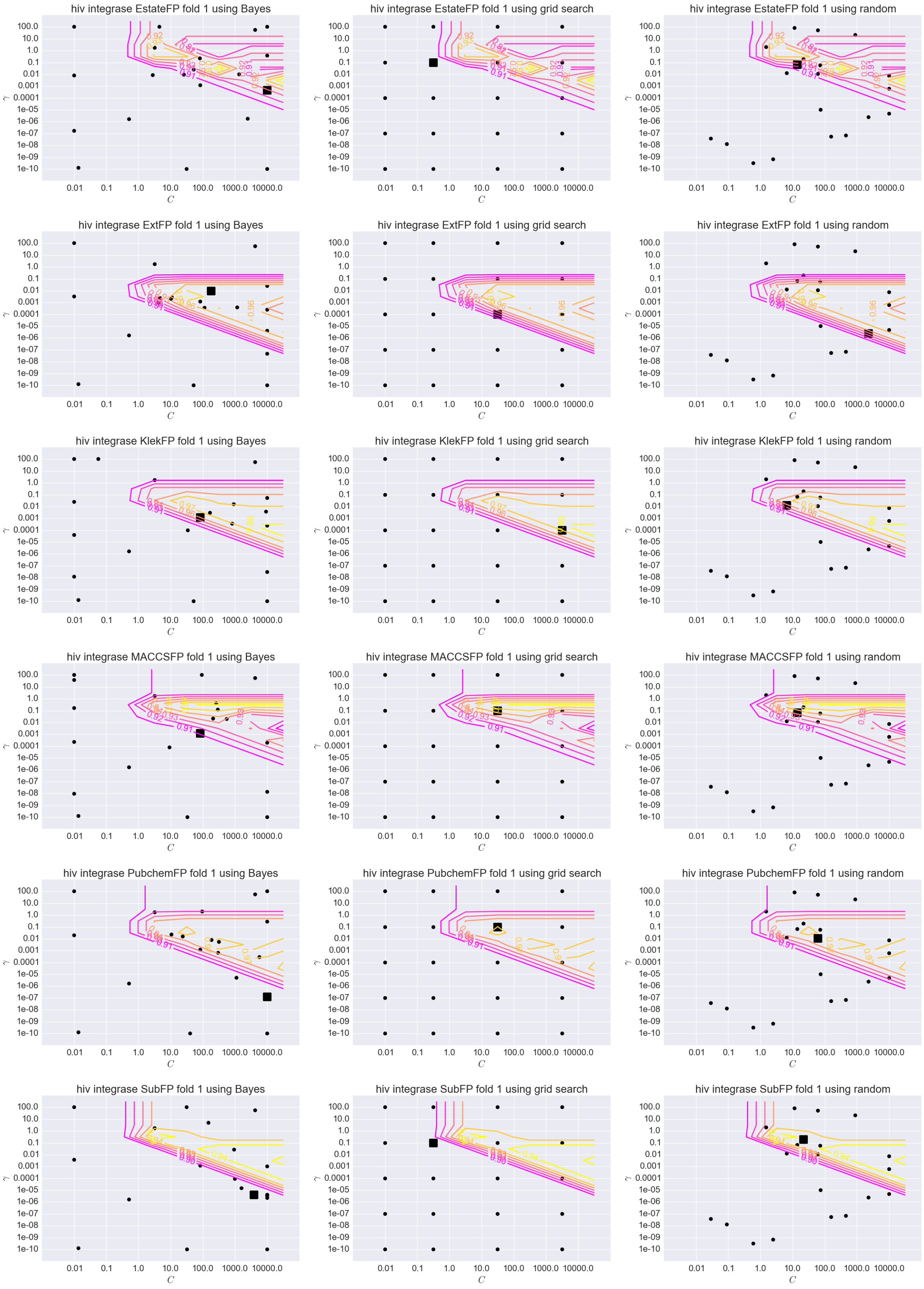

Figure 16: Analysis of the changes in accuracy for different steps for HIV integrase.

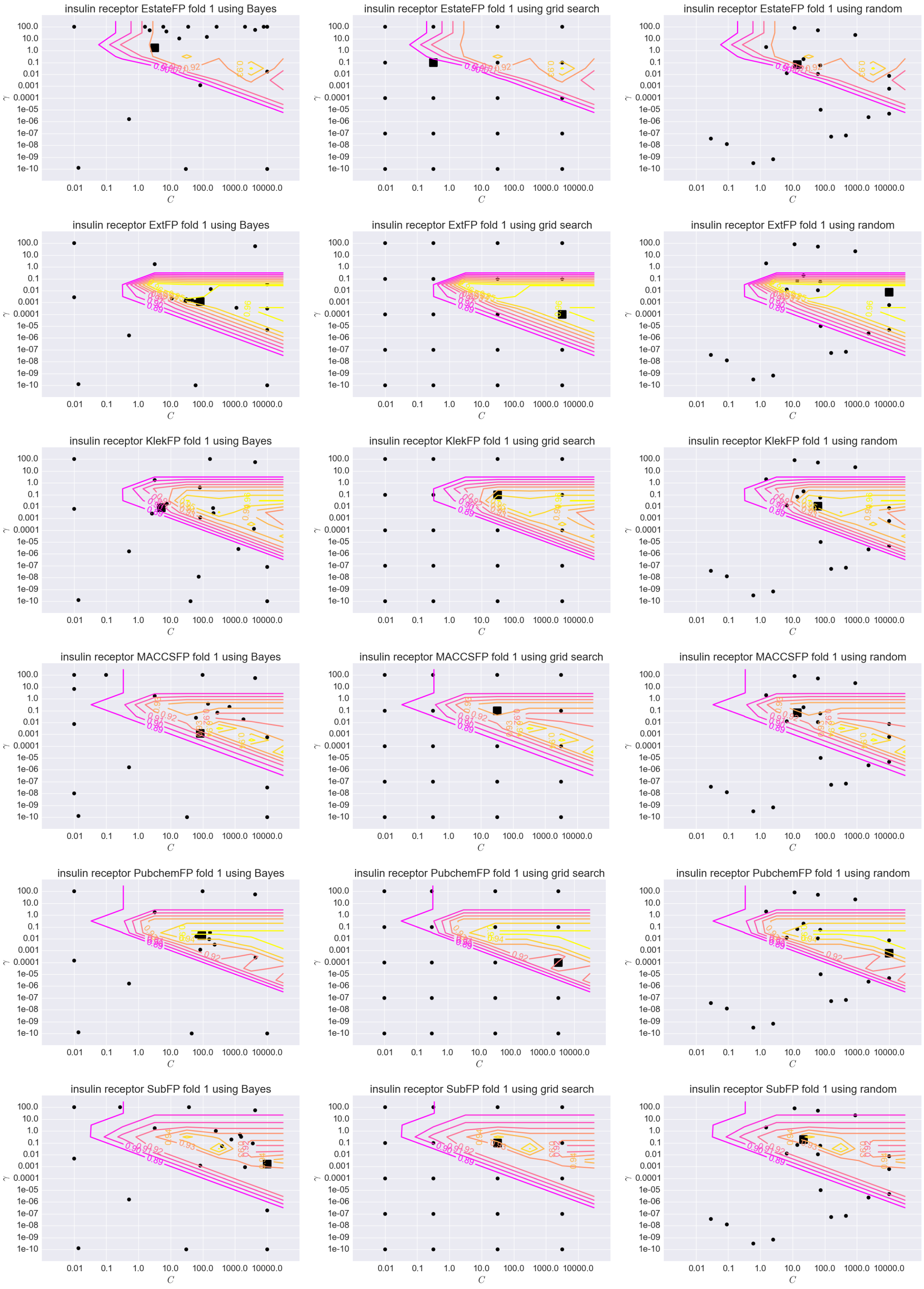

Figure 17: Analysis of the changes in accuracy for different steps for insulin receptor.

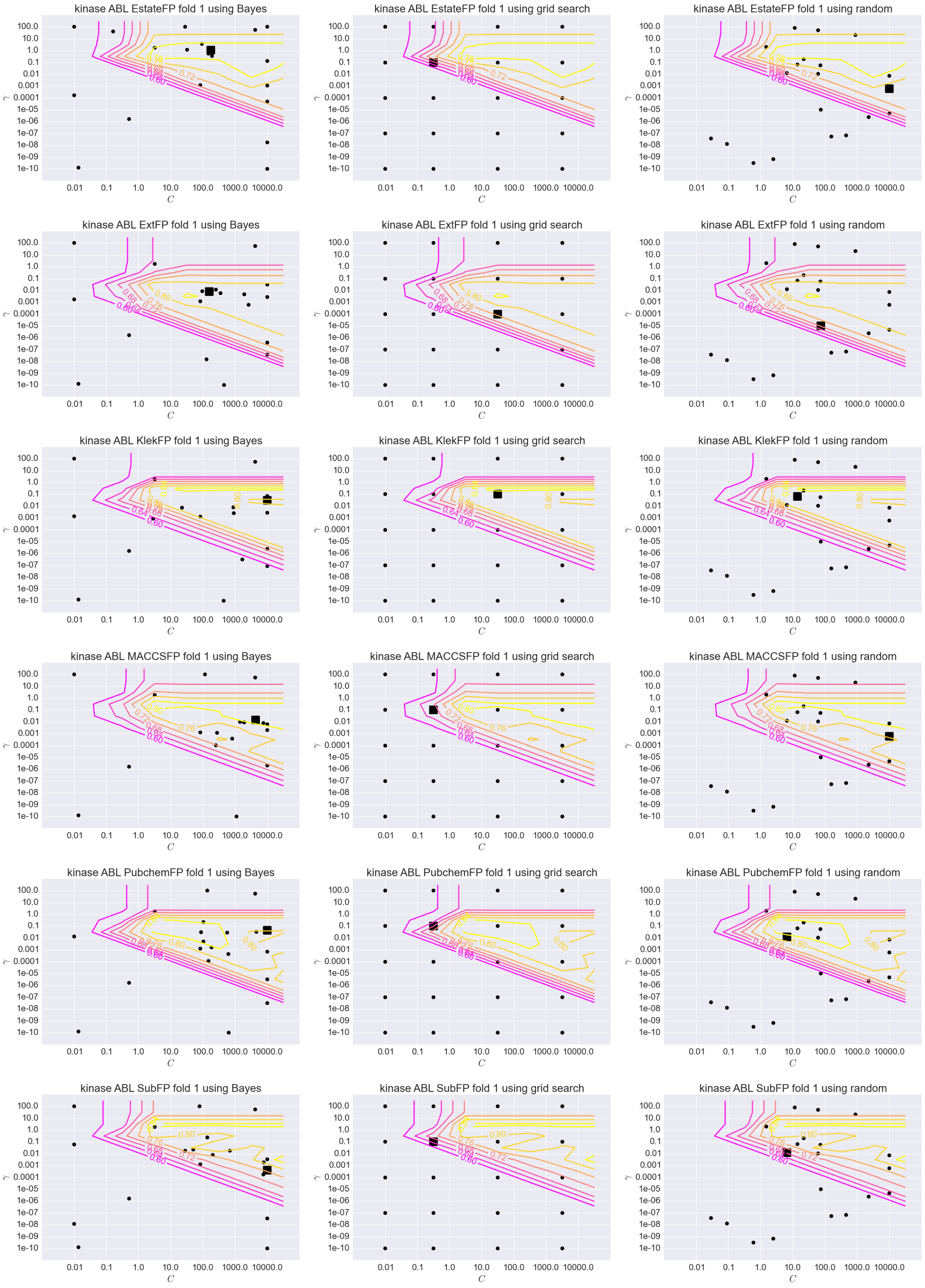

Figure 18: Analysis of the changes in accuracy for different steps for kinase ABL.

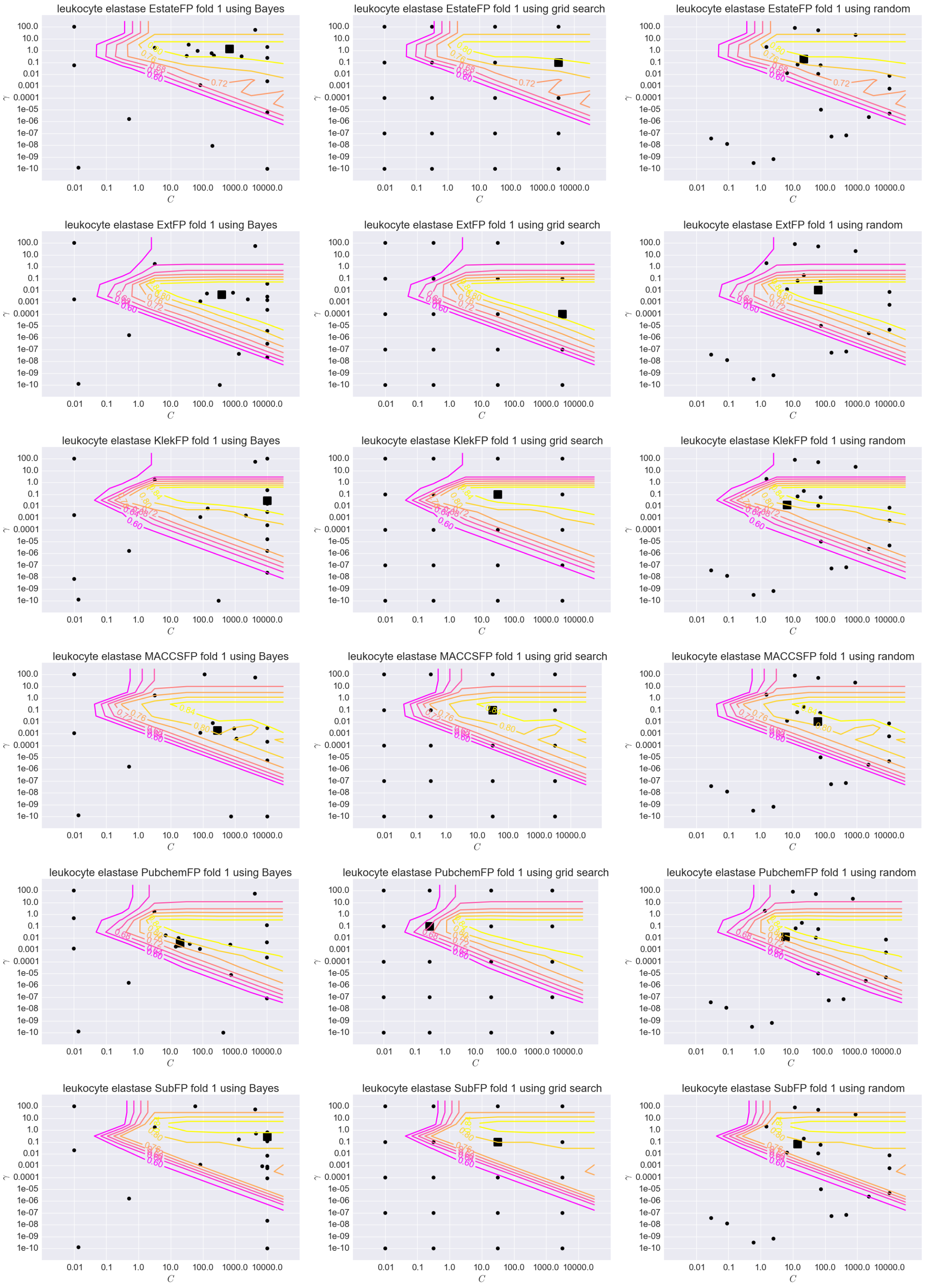

Figure 19: Analysis of the changes in accuracy for different steps for human leukocyte elastase.

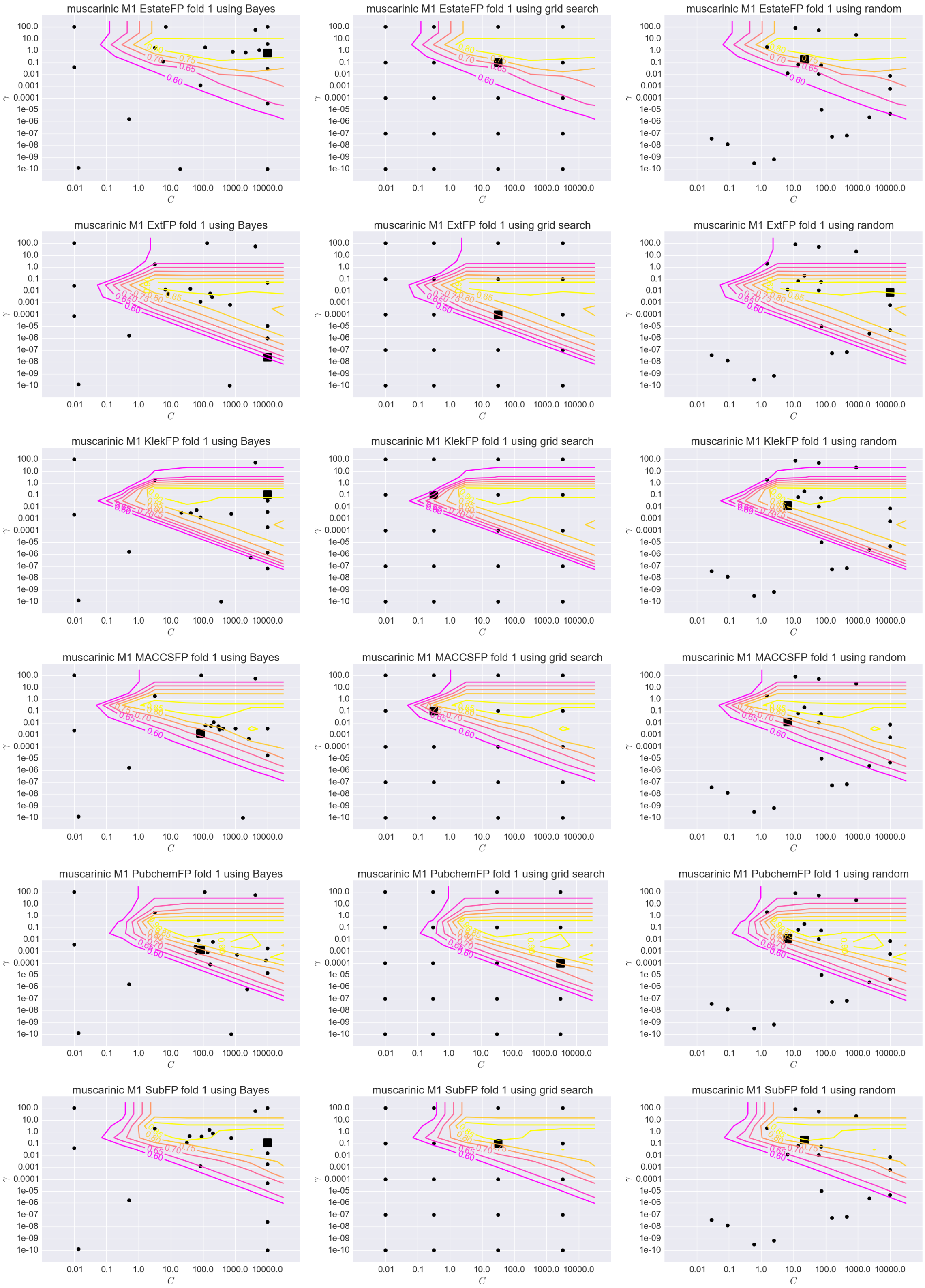

Figure 20: Analysis of the changes in accuracy for different steps for muscarinic M1 receptor.

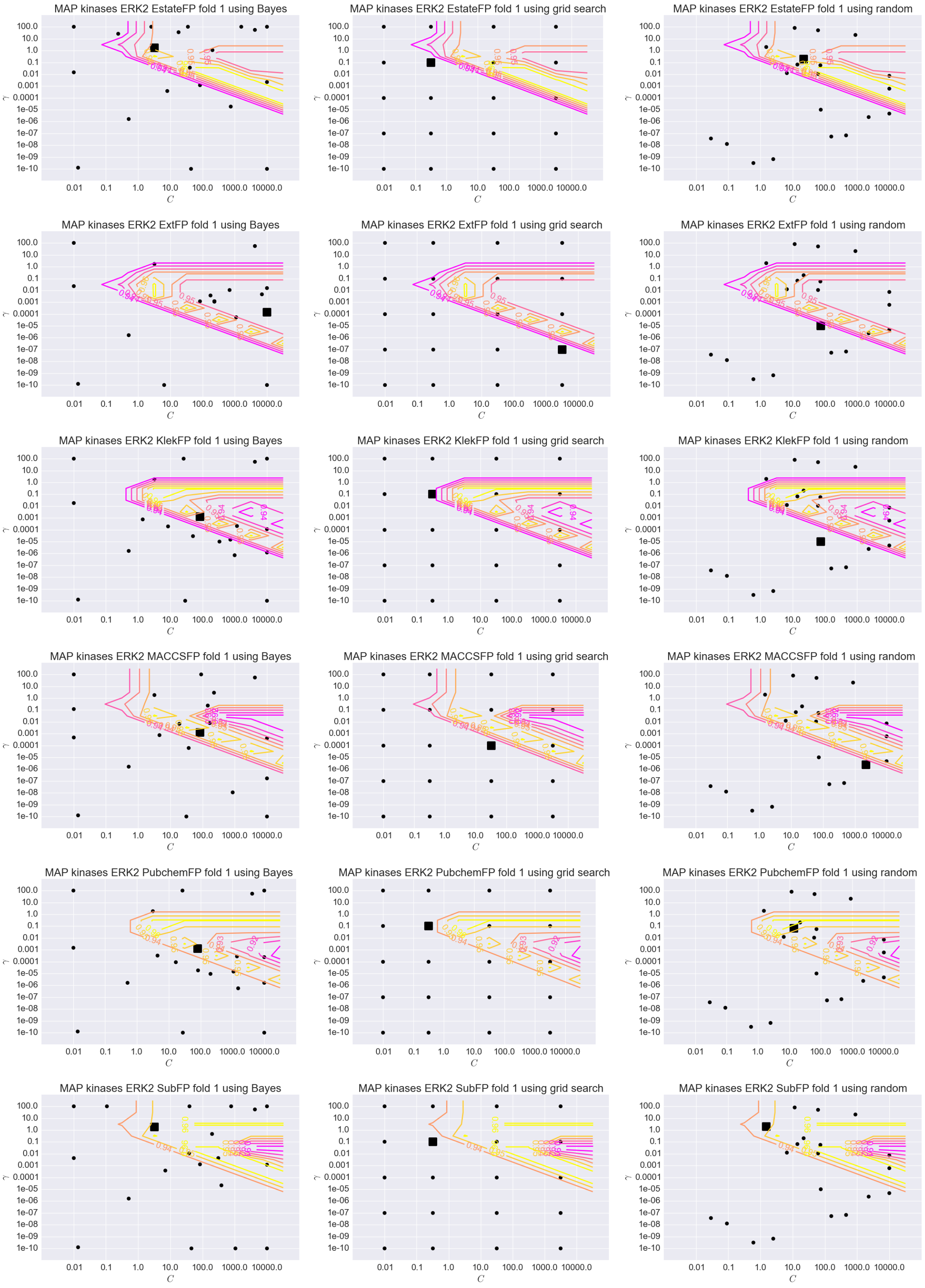

Figure 21: Analysis of the changes in accuracy for different steps for MAP kinases ERK2.
